# Repositioned Drugs for COVID-19—the Impact on Multiple Organs

**DOI:** 10.1007/s42399-021-00874-8

**Published:** 2021-04-21

**Authors:** Christina Chan, Sean T. Foster, Kayla G. Chan, Matthew J. Cacace, Shay L. Ladd, Caleb T. Sandum, Paul T. Wright, Brett Volmert, Weiyang Yang, Aitor Aguirre, Wen Li, Neil T. Wright

**Affiliations:** 1 Department of Chemical Engineering and Materials Sciences, Michigan State University, 428 S. Shaw Lane, Room 2100 EB, East Lansing, MI 48824 USA; 2 Department of Biochemistry and Molecular Biology, Michigan State University, East Lansing, MI USA; 3 Department of Biomedical Engineering, Michigan State University, East Lansing, MI USA; 4 Institute for Quantitative Health Science and Engineering, Michigan State University, East Lansing, MI USA; 5 Integrative Neuroscience Program, Binghamton University, Binghamton, NY USA; 6 Department of Mechanical Engineering, The Pennsylvania State University, University Park, PA USA; 7 Department of Mechanical Engineering, Virginia Polytechnic Institute and State University, Blacksburg, VA USA; 8 Department of Electrical and Computer Engineering, Michigan State University, East Lansing, MI USA; 9 Department of Mechanical Engineering, Michigan State University, East Lansing, MI USA

**Keywords:** COVID-19, Drug repositioning, Immune system, Lungs, Heart, Kidneys, Liver, Central nervous system

## Abstract

This review summarizes published findings of the beneficial and harmful effects on the heart, lungs, immune system, kidney, liver, and central nervous system of 47 drugs that have been proposed to treat COVID-19. Many of the repurposed drugs were chosen for their benefits to the pulmonary system, as well as immunosuppressive and anti-inflammatory effects. However, these drugs have mixed effects on the heart, liver, kidney, and central nervous system. Drug treatments are critical in the fight against COVID-19, along with vaccines and public health protocols. Drug treatments are particularly needed as variants of the SARS-Cov-2 virus emerge with some mutations that could diminish the efficacy of the vaccines. Patients with comorbidities are more likely to require hospitalization and greater interventions. The combination of treating severe COVID-19 symptoms in the presence of comorbidities underscores the importance of understanding the effects of potential COVID-19 treatments on other organs.

**Supplementary Information:**

The online version contains supplementary material available at 10.1007/s42399-021-00874-8.

## Introduction

Infection with the severe acute respiratory syndrome coronavirus 2 (SARS-CoV-2), an enveloped RNA virus and the cause of the coronavirus (CoV) disease 2019 (COVID-19) pandemic, manifests in a range of symptoms, predominantly fever, dry cough, and breathing difficulties, as well as fatigue, pneumonia, dizziness, and gastrointestinal tract (GI) symptoms [1, 2]. There is an overrepresentation of patients with severe COVID-19 illness who have underlying medical conditions, such as hypertension, cardiovascular disease, and diabetes [3–5]. COVID-19 can lead to acute respiratory distress syndrome (ARDS), acute respiratory failure, and significant rise in cytokine and chemokine levels, including cytokine storm syndrome [6], septic shock, and multiple organ dysfunction (MOD) [7–12]. These conditions are involved in the higher mortality of the most severe cases of COVID-19 and this increased risk of mortality is greater in older patients and patients with comorbidities, i.e., hypertension, diabetes, chronic obstructive pulmonary disease (COPD), cardiovascular disease, and cerebrovascular disease [7, 8, 13–15].

Beyond pneumonia, there are heart, liver, kidney, and neurological complications [1, 16]. With many patients, COVID-19 manifests functional damage to several organs, including acute kidney injury, cardiac and myocardial injury, liver dysfunction, and neurological disorders [7, 17]. Patients with pre-existing kidney, heart, or liver disease are more likely to suffer severe kidney, cardiac, or liver symptoms and complications. The involvement of these organs in COVID-19 is multifaceted, related to direct effects of the virus [17, 18] or to host immune response (inflammatory infiltrates), and in particular with the liver where there is also the potential of drug -induced liver injury [19].

The rapid growth of COVID-19 into an extraordinary pandemic has forced physicians and other health care providers to improvise in treating the most severe symptoms. This has included the off-label use of many drugs. While much is being learned about the unexpected effects of COVID-19, including uncommon effects, there has been the real time need to treat patients. These efforts have led to decreased morbidity in those presenting with COVID-19. Yet, as the pandemic continues, there is need for a review of the available drugs and their beneficial and adverse effects on systems in the body.

Additionally, we examine some of the effects of remdesivir (RDV) and chloroquine (CQ) on cardiac response in induced pluripotent stem cell (iPSC)–derived human heart organoids. Similarly, a recent study assessed the cardiotoxicity and QT prolongation of CQ-treated and RDV-treated human iPSC-derived cardiomyocytes [20] and found that RDV is associated with both cardiotoxicity and arrhythmogenic risk.

## SARS-CoV-2 Susceptibility and Disease Manifestation

### Viral Susceptibility

To assess the viral susceptibility of the various organs, researchers have measured the expression levels of the SARS-CoV-2, ACE2, and type II transmembrane serine protease 2 (TMPRSS2). SARS-CoV-2 spike (S) protein binds angiotensin-converting enzyme 2 (ACE2), which is an enzyme attached to the cell membranes of many cell types, and along with TMPRSS2, which primes the S protein by proteolytic cleavage of the S protein, promotes entry of the virus into host cells [21]. ACE2 and TMPRSS2 are expressed at varying degrees in numerous tissues, including the lungs, kidney, liver, heart, and brain [22–26] (https://www.proteinatlas.org/). Interestingly, ACE2 and TMPRSS2 have lower expression in airways of children as compared to adults [27, 28], while they are upregulated in cardiomyocytes of older adults [29]. This could contribute to the milder manifestation of the disease in children, but nonetheless the children show complement-mediated thrombotic microangiopathy levels indicative of blood vessel damage [30]. ACE2 is abundantly expressed in the lungs, small intestines [31], kidney, liver, and brain-endothelium and brain-vascular smooth muscle cells [31, 32]. ACE2 and TMPRSS2 are co-expressed in the respiratory tract (oral cavity and lungs) and highly co-expressed in the GI tract [27, 33–36]. They are also both expressed in the kidney [37] and were found in kidneys of patients with COVID-19 [38, 39]. ACE2 and TMPRSS2 levels were found to be expressed in liver progenitors cells with a cholangiocyte fate bias [40], which has been posited to compromise the regenerative capabilities of the liver [41]. While ACE2 is highly expressed in human heart, TMPRSS2 is less expressed there, although other proteases (e.g., cathepsin L and furin) are more highly expressed in the heart [42].

### Viral Infection

SARS-CoV-2 infection has been reported in the lungs [43–46] and other organs, including the kidneys, heart, liver, and brain [38, 39, 45, 47–49]. The viral load detected in the respiratory tract of COVID-19 patients has been positively associated with severity of the lung disease [50]. This is less clear with the other organs. SARS-CoV-2 has been found to directly infect engineered human kidney organoids, human-induced pluripotent stem cell–derived cardiomyocytes, cholangiocytes in human liver ductal organoids, and neuronal cells in human brain organoids (with a concomitant increase in cell death) [51–55]. However, the mechanism of viral entry is unclear.

There is evidence of direct viral infection of endothelial cells, including viral particles in the endothelial cells of the glomerular capillary loops of kidney tissues [18]; however, it is unclear whether SARS-CoV-2 infection of the kidneys leads to acute kidney injury (AKI) [56]. Nonetheless, AKI is observed frequently in COVID-19 patients and is associated with respiratory failure and poor prognosis [57]. A high prevalence of kidney disease on admission and development of AKI in hospitalized patients with COVID-19 were associated with in-hospital mortality [58].

The SAR-CoV-2 virus has been isolated from mycocardial and liver tissues [38, 49, 59], and SAR-CoV-2 infection has been detected in endomyocardial biopsies (EMBs) from a few patients [60]. Cardiac injury is a common condition among hospitalized COVID-19 patients and underlying cardiovascular disease (CVD) with myocardial injury is linked to increase mortality with COVID-19 [61, 62]. Whether viral entry into the heart leads to the cardiac complications and dysfunction observed in COVID-19 is actively being studied. A study of iPSC-derived cardiac cells exposed to SARS-CoV-2 virus led to cytopathic changes in the cells supporting that viral infection could lead to cardiac damage [63].

Likewise with the liver, hypoalbuminemia and abnormal liver biochemistries are associated with higher rates of complications and mortality and worse recovery [64, 65]. It also has been suggested that injury to the liver could be due more to the immune response than virus itself [66]. However, an ultrastructural analysis of livers of COVID-19 patients with abnormal liver enzymes suggests that the SARS-CoV-2 infection contributes to cytopathic lesions, lending support that infection in the liver contributes to hepatic impairment [67].

Understanding viral infection of the brain by SARS-CoV-2 is actively being investigated. Meningitis, encephalitis, encephalopathy, loss of smell, altered taste, headache, and dizziness are all suggestive of potential neural involvement and have been reported in COVID-19 patients [68–70]. The SARS-CoV-2 virus has been detected in neural and capillary endothelial cells in the frontal lobe tissue [71] and in the cerebrospinal fluid (CSF) of some COVID-19 patients [69], albeit not necessarily in the cases with severe neurological complications [72]. Evidence of neuroinvasion was found with SARS-COV and MERS-CoV lending support to a potential of viral entry into the brain in contributing the neurological manifestations of COVID-19 [73]. Along these lines, a study found that the spike protein of SARS-CoV-2 can cross the blood brain barrier [74]. Given that the loss of taste and smell is one of the common first symptoms of COVID-19, it is unsurprising that SARS-CoV-2 has been found to enter the CNS via the olfactory system, which is likely due to the proximity of neurons to the thin submucosal lining [75]. Indeed, a recent study found evidence of neuroinvasion of SARS-CoV-2 in brains of humans and mice and administering ACE2 antibody mitigated neuronal infection [76].

### Role of Inflammation

In addition to viral infection, host inflammatory responses contribute to the disease manifestation of COVID-19. Accumulation of inflammatory cells has been found in the lungs, heart, kidneys, and liver [18, 39]. The lungs are targeted by SARS-CoV-2, with both immediate and potentially lasting consequences. COVID-19 is characterized by respiratory symptoms that can lead to respiratory failure [77]. ARDS is a common cause of death in critically ill COVID-19 patients [78]. ARDS, sepsis, and MOD all contribute to lung complications in COVID-19 patients. SARS-CoV-2 infection could have both direct cardiovascular and indirect cardiovascular consequences, including myocardial injury, acute coronary syndromes, cardiomyopathy, arrhythmias, and inflammation [17, 79]. Additionally, inflammation is a potential contributor to myocarditis in COVID-19 [80, 81]. Patients with COVID-19 are known to have increased inflammatory responses [82]. In the heart, this increase in cytokines modifies K^+^ channels, which can disrupt cardiac action potentials, resulting in lethal cardiac arrhythmias [79, 83]. Older patients with cardiovascular comorbidities and diabetes that contract COVID-19 are at higher risk of myocardial injury and mortality [61, 62]. Athletes are not immune to cardiovascular manifestation of COVID-19, with evidence by reports of myocardial injury (including myocarditis) [84]. Additionally, previously infected individuals can have ongoing myocardial inflammation after recovering from COVID-19 [85]. It has not been conclusively determined if kidney injury in COVID-19 patients are from direct viral involvement in the tissue or the accumulation of inflammatory cells and the host inflammatory response (cytokine storm). Factors that contribute to AKI include systemic hypoxia, infiltration of inflammatory cells, abnormal coagulation, and possible drug effects [39]. Similarly, it remains unclear if liver damage is caused by direct viral infection or immune system activation and the resulting cytokine storm or drug effects [86]. The effects on liver observed in patients include elevated enzyme levels, hepatocellular necrosis, and moderate microvesicular steatosis [86, 87]. Additionally, in a retrospective patient study, therapeutic use of lopinavir/ritonavir for COVID-19 was associated with liver damage [87]. Increases in systemic cytokine levels due to SARS-CoV-2 infection could contribute to central nervous system (CNS) dysfunction [88, 89]. Neurological damage resulting from COVID-19 infection includes anosmia, encephalopathy, inflammatory CNS syndromes, ischemic strokes, and peripheral neurological disorders. In particular, there was a high incidence of acute disseminated encephalomyelitis with hemorrhagic change, which was not associated with severity of the disease [88, 90]. Therefore, both the viral infection and host inflammatory responses could contribute to the lung-, heart-, liver-, and kidney-related complications in COVID-19.

## Drug Repositioning

Although innovations in care have improved survival rates of severe cases, there remains no definitive treatment or cure for COVID-19. Given the urgency of this pandemic, multiple pharmaceutical treatment modalities are being pursued in the form of vaccine and repurposing of existing drug therapies. Given the lengthy process involved in vaccine development, drug therapies are vital in mitigating the more severe responses of COVID-19, thereby necessitating viable strategies to search for new classes of medicines. Repositioning or repurposing FDA-approved drugs is a feasible approach to discover new therapies to respond quickly to COVID-19 than de novo drug development which takes many years. Drug repositioning offers shorter route to the clinic and have well-known safety concerns since they have been through several stages of clinical development [91]. The range of drugs that have been proposed for treating COVID-19 includes (1) anti-virals that interfere with viral entry, synthesis, or replication (including protease inhibitors), (2) immunomodulatory agents that affect cytokine levels and host inflammatory responses (e.g., antibiotics, antihistamines, steroids, and monoclonal antibodies) that could affect respiratory distress syndrome or cytokine storm, (3) antiparasitic drugs (antihelmintic), and (4) vasodilators. Table 1 summarizes this range of drugs and Supplementary Table 1 provides their classification. Most of the drugs are beneficial to the immune system, reducing inflammation and attenuating viral infection in the lungs, while their effects across the other organs (heart, kidneys, liver, and CNS) are not uniformly beneficial.

**Table 1:**
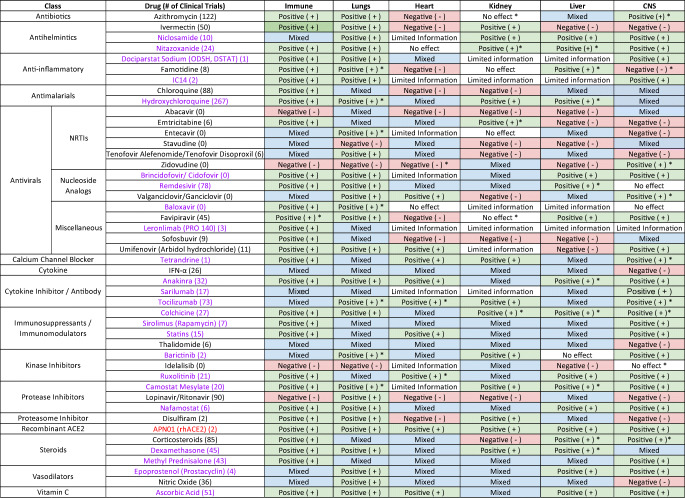
Summary of drugs’ effects on diverse organs

*A case study, instance, or minor effect that is opposite as noted. When indicated with “No effect”, it denotes a case study or instance of either positive or negative results. Drugs in purple do not have (only) negative results but mixed ratings of both positive and negative results reported for the organ. Red indicates positive results across all the organs for the drug

### The Effects of Drugs on Various Organs

**Fig. 1:**
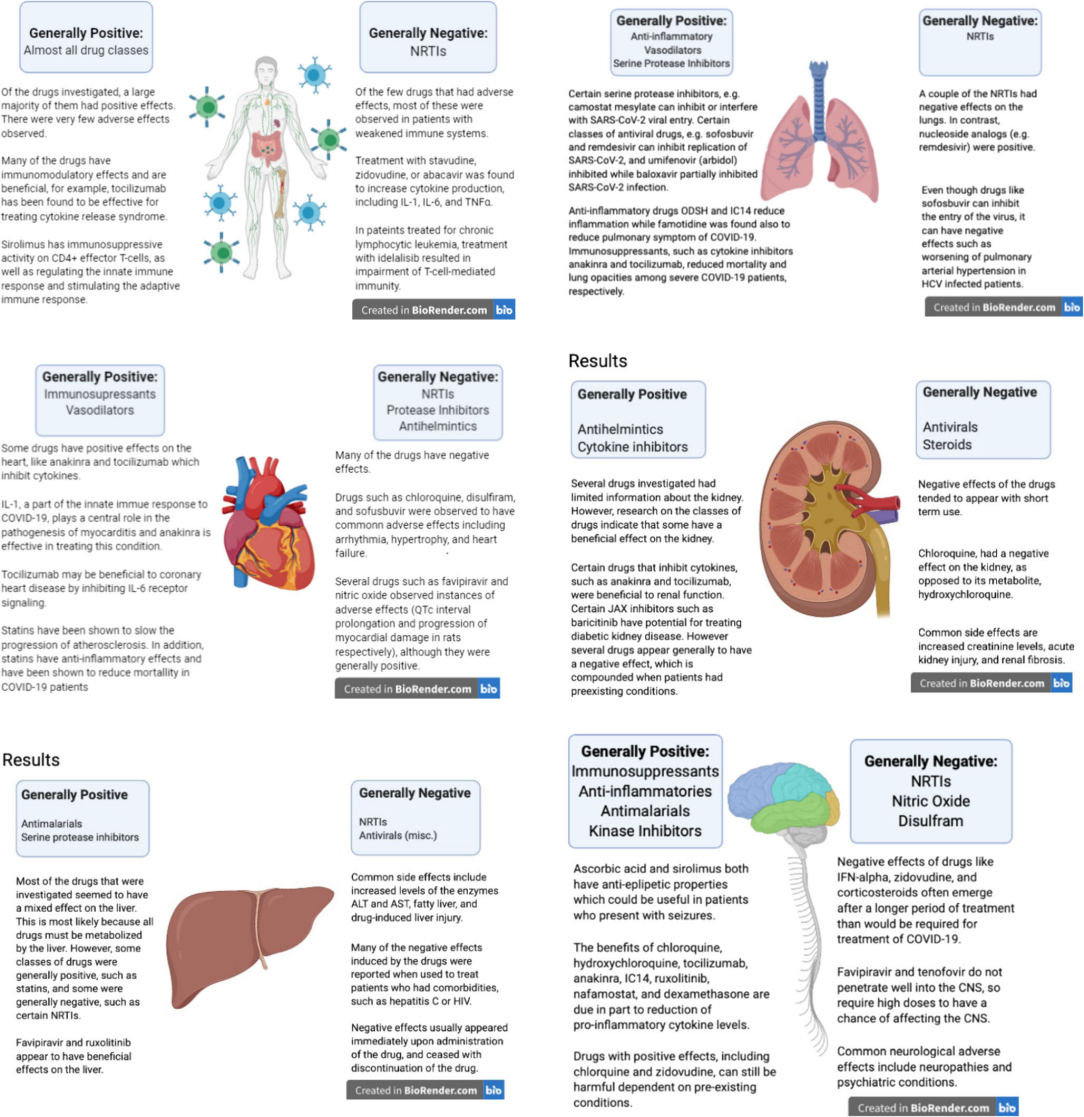
The effects of certain drugs on the immune system and individual organs (lungs, heart, kidneys, liver, and CNS)

In light of the manifestation of COVID-19 on various organs, it is important that the drugs used to treat COVID-19 patients do not exacerbate symptoms associated with the disease. This suggested the benefit of gathering a list of drugs that are being used off-label or in clinical trials for COVID-19 and searched for potential beneficial and harmful effects of these drugs on the immune system, lungs, heart, kidneys, liver, and CNS, when used to treat specific conditions or diseases. Search of PubMed, Google Scholar, Google, bioRxiv, and medRxiv, using the organ and name of the drug(s) and disease(s) as keywords, revealed multiple aspects of the drug interactions with various organs and systems. The goal is to provide an extensive depiction of how the drugs could have differential effects on the various organs, beneficial to some while harmful to other organs. To a lesser extent, this analysis could suggest situations in which specific drugs could be counter-indicated under certain conditions. The goal was to identify any positive or negative effects on these organs from the treatment of these drugs for any disease, including COVID-19, and potentially future infectious diseases. Table 1 provides a summary of each drug’s effects on these organs. Supplementary Tables 2-7 show in greater detail the effects of the drugs on the (1) immune system, (2) lungs, (3) heart, (4) kidneys, (5) liver, and (6) CNS. Several observations are apparent. First, many of the repositioned drugs are beneficial to the immune system and the lungs, but have mixed effects across the other organs. This is unsurprising as the drugs are predominantly immunomodulators or anti-virals and breathing difficulty and cytokine storm are major manifestations of COVID-19 that are targeted by the drugs. Second, few of these drugs are beneficial across all vital organs. Of the drugs evaluated in this study, APN01, a recombinant human ACE2, appears to have positive effects reported across the organs evaluated in this study. Third, patients with comorbidities are often more vulnerable to COVID-19 infection. These patients are most likely to show severe symptoms requiring hospitalization and treatment, underscoring the importance on being aware of the effect that the repositioned drugs have on various organs. For each of the organs, a summary of the findings is depicted in Fig. 1. For the immune system, most of the drugs have a beneficial effect. The drugs are generally immunosuppressive and function by reducing the cytokines released by both the innate and adaptive immune systems that contribute to COVID-19 pathology. These immune systems play an integral part in combatting cytokine release syndrome (CRS) of COVID-19. The few drugs with negative effects on the immune system were typically immunostimulatory, and adverse effects were most often observed in individuals with impaired immune systems (Supplementary Table 2). For the lungs, most of the drugs in this study had beneficial effects, nonetheless about a third of the drugs reviewed reported both positive and negative effects on the lungs. Anti-inflammatories, vasodilators, and serine protease inhibitors generally were helpful. In contrast, a couple of the nucleoside/nucleotide reverse transcriptase inhibitors (NRTIs) primarily affected the lungs negatively (Supplementary Table 3). For the heart, several drugs had reported favorable effects on the cardiac system. Most of these benefits related to improving the cardiovascular system or indicate limited risk of cardiac injury for COVID-19 patients. For example, statins reduced the progression of atherosclerosis, and in hospitalized COVID-19 patients, statins did not increase risk of cardiac injury. However, a majority of the drugs reported both positive and negative effects or primarily negative effects on the heart. In fact, many of the drugs have not been thoroughly investigated for their cardiovascular effects. Of these drugs, some of the more common adverse effects were hypertension, congestive heart failure (CHF), myocarditis, and QT interval prolongation. For example, AZM was associated with myocarditis and QT interval prolongation (Supplementary Table 4). For the kidneys, the drugs that report both positive and negative impact on the kidney or predominantly negative effects tend to appear in patients with autoimmune conditions such as HIV, hepatitis, or rheumatoid arthritis (RA). In healthy patients, these drugs may be less deleterious (Supplementary Table 5). For the liver, most of the drugs reported negative or both positive and negative effects due to its role in drug metabolism. Considering the frequency of liver dysfunction in COVID-19 patients, attributing hepatotoxicity to the drug vs. the underlying disease can be difficult. Most adverse effects were not severe, and resolved upon ceasing treatment. The most common negative effects were increases in liver enzymes and drug-induced liver injury (Supplementary Table 6). For the CNS, of the drugs studied, most had a positive effect on the CNS, particularly the immunosuppressant drugs and cytokine inhibitors. The only drugs as a class that were primarily negative to the CNS were NRTIs. The most common neurological side effects were headaches, dizziness, and fatigue, which in most cases were not severe enough to discontinue treatment (Supplementary Table 7).

### Long-term Effects of the Drugs

Studies on the long-term outcomes of the 2003 SARS indicate that there are consequences of the infection and of the drugs used to treat them, ranging from metabolic to pulmonary fibrosis to femoral head necrosis to neurological [92–98]. Although it is too early to know whether some of the symptoms that persist in recovered COVID-19 patients will be long lasting, it does raise concerns on the potential of not only the virus itself but of the treatments, including certain drugs currently being used off-label or in clinical trials to treat COVID-19, that could contribute to exacerbating these symptoms [99]. For example, the use of corticosteroid could help suppress lung inflammation but there is a concern that it could inhibit host immune response. In a 15-year follow-up of SARS patients treated with high-dose steroid pulse therapy, the femoral head necrosis was found not to be progressive and to show some recovery [94]. The existing evidence based on past experiences with SARS and MERS suggest that the use of steroids is inconclusive and caution is recommended as a routine treatment [100, 101]. Nonetheless, in cases of hyperinflammation, immunosuppression (including interleukin antagonists) could be beneficial without increased adverse effects [6]. As with any drug, there is the potential for drug-induced liver injury [102]. Drugs that could increase the risk of liver injury based on the medical condition of the individual [103–106]. Long-term damage of the viral infection could lead to chronic illness that impact not only the lungs but also the heart, immune system, brain (mental), and other organs. Therefore, we search in the literature to find if any of the drugs on our list (Table 1) have been associated with inducing chronic effects or have negative effects upon long-term administration of the drug (Table 2). The most common persistent effects after COVID-19 are fatigue and dyspnea [99, 130, 131]. Therefore, we searched in the literature to identify drugs that affect chronic fatigue syndrome (CFS) and indicate possible drugs that could either exacerbate or mitigate CFS (Table 3).

**Table 2:** Potential long-term effects of drugs

Drug	Long-term effect
Brincidofovir/cidofovir	Prolonged use can cause severe nephrotoxicity in a monkey study [107].
DEX	Prolonged use can cause fatty liver and diabetes in mouse models [108], as well as microvesicular steatosis [109]. May induce long-term negative effects on neuromotor function and somatic development in male infants [110].
DS	Several sources indicate that DS can cause neuropathies in patients if used at high doses or for long periods. While the long period may not be an issue for COVID treatment, this is still something to consider. The symptoms do resolve after treatment is discontinued [111].
EVT	While uncommon, neuropathy is a potential side effect of EVT if given for long periods, at high dosages, or to patients with other risk factors for neuropathies [112].
EPO	Intravenous EPO improves long-term survival in primary pulmonary hypertension [113].
HQ/CQ	Increased risk of retinopathy was observed with high-dose and long-term (5+ years) use of HCQ [114]. These drugs interact with lysosome activities which may contribute to retinopathy and cardiotoxicity [115–117].
MP	Possible long-term bone metabolism effects in patients with MS [118].
RUX	A case study of a patient with MF-associated pulmonary hypertension (PH) developed left ventricular systolic dysfunction after long-term (5 years) treatment with RUX [119].
Statins	Long-term use of statins is associated with inhibiting the progression of aortic stenosis and aortic stiffness [120, 121].
Stavudine	Stavudine was found to reduce N-acetylaspartate (a marker of mitochondrial function) in the frontal lobe of HIV patients. Stavudine has a mitochondrial toxicity in the brain, which worsens the longer the drug is administered [122].
THD	Long-term treatment (>1 year) with THD of multiple myeloma patients has shown to have toxicity including neurotoxicity [123]. When used to treat dermatologic disorders (prurigo nodularis and aphthous stomatitis), long-term use led to peripheral neuropathy. Thus, only short-term use is recommended due to neurotoxic effects [124]. In lupus patients, THD neuropathies are potentially irreversible after discontinuing treatment [125].
AZT	Long-term treatment with AZT may induce mitochondrial toxicity in HIV patients [126]. They may also induce in HIV patients anemia [127], myalgia, muscle weakness, and increased serum creatine kinase levels [126]. Adverse neurological effects of AZT are rare but can be serious, including seizures, dose-reduction encephalopathy, and myopathy. The likelihood of these adverse effects occurring increases the longer the drug is administered [128]. Long-term monotherapy with AZT has been reported to induce fatal lactic acidosis and hepatotoxicity in case reports [129].

**Table 3:** Drugs that impact chronic fatigue syndrome

Drug	The effect on chronic fatigue
Anakinra	Anakinra does not lessen chronic fatigue syndrome (CFS) [132].
AA	Vitamin C reported to reduce fatigue in office workers [133].
AZM	Patients with CFS who responded to AZM reported a decrease in the symptoms and lower levels of plasma acetylcarnitine [134].
Baricitinib	In a randomized, double-blind, phase 3 clinical trial, baricitinib resulted in reductions of pain and fatigue, and improved daily activity and work productivity compared to placebo with RA patients [135].
Colchicine	Colchicine associated with drug-induced fatigue [136].
Corticosteroid	Corticosteroid did not improve the severity of associated CFS symptoms in CFS patients who do not have allergic rhinitis [137].
DEX	DEX was effective in treating cancer-related fatigue (CRF). DEX acts rapidly in relieving CRF in patients with advanced cancer [138].
Emtricitabine	Case study found fatigue was attributed to HIV patients switching back from emtricitabine to lamivudine [139].
EPO	Fatigue can be a symptom of pulmonary hypertension. EPO has been both associated with fatigue [140] at high doses as a side effect, as well as improving fatigue [141].
Famotidine	Case study of COVID19 patients treated with famotidine showed quicker improvements in clinical symptoms other than fatigue, with one instance of increasing fatigue [142].
Favipiravir	A study comparing favipiravir and arbidol found more COVID19 patients on favipiravir suffered from fatigue albeit not statistically significant (P value 0.0579) [143].
HCQ	HCQ treatment for Sjogren’s syndrome (pSS) found that when treating fatigue related to pSS, HCQ was no different than placebo [144].
IC14	Patients with myalgic encephalomyelitis/CFS have elevated soluble CD14 in their blood. As an anti-CD14 monoclonal antibody, it could be explored for its potential on chronic fatigue [145].
IFN	CD4 T cells from CFS patients produced less interferon-γ than did cells from controls [146].
IFN-α	Can induce persistent fatigue in some patients. There is an increase in the levels of IL-6 and IL-10 concurrent with IFN-α treatment [147]. IFN-α treatment of CFS patients improved for a subset of patients with diminished natural killer (NK) function. IFN-α increased NK function [148].
Ivermectin	A case report of fatigue developed with ivermectin treatment [149]. Ivermectin along with other anti-virals are associated with fatigue [150].
Leronlimab	Fatigue has been reported with monoclonal CCR5 antibody albeit not specifically with leronlimab [151].
LPV/RTV	LPV/RTV is associated with improvements in fatigue [152].
NTZ	NTZ used in treating diarrhea and enteritis associated with blastocystis hominis reported fatigue as an adverse event [153].
NO	NO metabolites (nitrates) levels are elevated in CFS patients [154]. In contrast, a study found NO levels are similar in CFS and control patients and unrelated to CFS [155].
RUX	Treating myelofibrosis patients with RUX has been associated with instances of fatigue or increasing fatigue [156, 157].
Sarilumab	Reported to improve fatigue in RA patients [158].
Sirolimus (rapamycin)	Sirolimus treatment of complex vascular malformations [159] as well as sirolimus-associated pneumonitis in renal transplant patients is associated with fatigue. Discontinuation of sirolimus resulted in recovery within 6 months [160].
Sofosbuvir	Adverse effects of sofosbuvir in treating chronic hepatitis C patients include fatigue [161].
Statins	Statin is associated with exertional fatigue [162]. Coenzyme Q10 (CoQ10) was significantly lower in patients with myalgic encephalomyelitis (ME)/CFS and was associated with fatigue. Statins decrease CoQ10 levels, so statins could be counterproductive [163].
Tenofovir	Fatigue is one of the more commonly reported adverse events in treating chronic hepatitis B patients with tenofovir disoproxil fumarate [164] or tenofovir alafenamide [165].
THD	One of the common side effects of THD use is fatigue [166, 167].
TCZ	TCZ was effective in reducing the disease activity and improving fatigue in patients with RA. The hypothalamic-pituitary-adrenal (HPA) axis activated by IL-6 and IL-6 blocking agents has been shown to relieve fatigue in RA patients [168]. TCZ as an anti-IL-6 receptor may explain how it could mitigate fatigue.
Umifenovir (Arbidol)	Fewer COVID19 patients on arbidol reported fatigue as compared to favipiravir but it was not statistically significant (*P* value 0.0579) [143].

### Clinical Trial Results

Many of these drugs have undergone or are undergoing clinical trials. Some drugs have few published results. Clinical trials of each drug for treatment of COVID19 listed clinicaltrials.gov included available, not yet recruiting, recruiting or enrolling by invitation, active-not yet recruiting, recruiting, suspended, terminated, withdrawn, and completed for the drugs listed in Table 1 as of January 11, 2021. A search for trials with publications of the results was performed. For each peer-reviewed publication, we searched for the NCT number and confirmed the publication contains controlled and randomized results from the trial. Table 4 collates the current results of COVID-19 clinical trials that are completed for drugs that report results in peer-reviewed publication.

**Table 4:** COVID-19 clinical trial results

Drug	COVID-19 clinical trial summary (NCT #)
ACE2 (recombinant human)	A two-part phase II trial comprising an open-label intrapatient dose escalation and a randomized, double-blind, placebo-controlled phase in intensive care units of COVID-19 patients found rhACE2 markedly reduced angiotensin II levels [169] (NCT01597635).
AZM	In a randomized clinical trial in Brazil, AZM used in combination with standard care, which included HCQ, did not result in a statistical difference in recovery time. Clinical trial did not note any significant increase between the control and AZM in arrhythmia, cardiac arrest, acute kidney failure, or QT interval prolongation [170] (NCT04321278).
Baricitinib	No clinical trials. Some cite baricitinib as a frequent cause of co-infection leading to increased mortality (PRAVEEN; PUVVADA; M, 2020). Link A double-blind, randomized, placebo-controlled clinical trial of baricitinib plus RDV was better than RDV alone in improving recovery time and clinical status of COVID-19 patients [171] (NCT04401579).
Colchicine	A prospective, open-label, randomized clinical trial of 105 patients in Greece noted significant clinical benefit of colchicine in COVID-19 hospitalized patients; however, there were no significant differences in cardiac troponin or CRP levels in the treated vs. control group [172] (NCT04326790).
Corticosteroids	Preliminary results of a clinical trial suggest hydrocortisone improved the time for patients to be organ support free; however, the trial was stopped early because no treatment strategy met prespecified criteria for statistical superiority, barring definitive conclusions. [173] (NCT02735707)
DEX	In a controlled, open-label trial, DEX reduced the 28-day mortality in hospitalized COVID-19 patients among those receiving invasive mechanical ventilation or oxygen but not in those without respiratory support [174] (NCT04381936). In a multicenter, randomized, open-label, 28-day clinical trial of intensive care units in Brazil of patients with COVID-19 and moderate to severe ARDS, DEX was effective at increasing the number of ventilator-free days in patients when used in conjunction with standard of care [175] (NCT04327401).
HCQ	In a multicenter, randomized, open-label, controlled trial of hospitalized patients with mild-to-moderate COVID-19 found the use of HCQ alone, or with AZM, did not improve clinical status at 15 days as compared with standard care [176] (NCT04322123). A randomized, double-blind, placebo-controlled clinical trial at 2 tertiary urban hospitals found no clinical benefit treating with HCQ daily for 8 weeks pre-exposure prophylaxis of health care workers [177] (NCT04329923). A randomized, double-blind, placebo-controlled clinical trial concluded that HCQ did not prevent illness after high-risk or moderate-risk exposure to COVID-19 as compared to the placebo group. Additionally, HCQ did not significantly reduce symptom severity in patients with early, mild COVID-19 [178] (NCT04308668). In a multicenter, blinded, placebo-controlled randomized clinical trial conducted at 34 hospitals in the USA of hospitalized adults, HCQ was not found to be effective at improving clinical status after 14 days as compared to placebo. Patients receiving HCQ had numerically higher but not statistically significant instances of adverse events compared to the placebo group [179] (NCT04332991).
Combination of IFNβ-1B, LPV, and ribavirin	A multicenter, prospective, open-label, randomized, phase 2 clinical trial of COVID-19 patients in six hospitals in Hong Kong treated with combination of IFNβ-1b, LPV–RTV, and ribavirin found the triple therapy to be statistically more effective at shortening hospital stay and viral shedding than just LPV–RTV for patients with mild-to-moderate COVID-19 [180] (NCT04276688).
Ivermectin	A pilot clinical trial of hospitalized patients with mild-to-moderate COVID-19 treated with the addition of ivermectin to HCQ and AZM had a shorter hospitalization period and no adverse effects Ivermectin therapy added to HCQ and AZT was more effective, shortening the length of the hospital stay, and with no obvious adverse events. However, the study was limited to a small number of patients [181] (NCT04343092).
LPV/RTV	A randomized, controlled, open-label, clinical trial did not find LPV/RTV successful in reducing duration of hospital stay, or mortality rate, or risk of progressing to invasive mechanical ventilation [182] (NCT04381936). In another randomized controlled trial, arbidol monotherapy treatment of mild-to-moderate COVID-19 patients did not significantly improve clinical outcome [183] (NCT04252885).
MP	A clinical trial of multicenter observation study exploring association between exposure to prolonged, low-dose MP treatment and need for ICU referral, intubation, or death within 28 days found early administration of prolonged MP was associated with reduced hazard of death and ventilator dependence [184] (NCT04323592). A single pretest, single posttest quasi-experiment in a multicenter health system in Michigan found early short course of MP in moderate to severe COVID-19 patients may prevent disease progression and improve clinical outcomes [185] (NCT04374071). In a double-blind, placebo-controlled, randomized, phase IIb clinical trial in Brazil found short-term MP was not effective at reducing mortality rates. In the trial, patients meeting ARDS criteria were also treated intravenous with ceftriaxone plus AZM or clarithromycin. The trial did note that MP was significantly effective at reducing mortality rates in patients over the age of 60 [186] (NCT04343729).
RDV	A randomized, open-label, phase 3 trial of hospitalized severe COVID-19 patients with radiologic evidence of pneumonia not requiring mechanical ventilation treated with RDV did not show a significant difference between a 5- or 10-day treatment, and no placebo control was included [187] (NCT04292899). A randomized, open-label trial of hospitalized COVID-19 patients with confirmed severe ARDS did not find statistically significant clinical benefit with RDV as compared to standard care. Nausea, hypokalemia, and headache were more frequent in the RDV group [188] (NCT04292730). A double-blind, randomized, placebo-controlled trial of intravenous RDV in patients hospitalized with COVID-19 showed a significant difference in recovery time and reduced respiratory tract infection. More serious adverse events were reported for the placebo group than for the RDV group [189] (NCT04280705). A randomized, double-blind, placebo-controlled, multicenter trial at ten hospitals found RDV was not associated with a statistically significant difference in clinical benefits and was stopped due to more adverse events as compared to placebo [190] (NCT04257656).
Sarilumab	In an ongoing international, multifactorial trial, critically ill COVID-19 patients receiving organ support in intensive care treated with sarilumab (an IL-6 receptor antagonist) improved survival [191] (NCT02735707).
TCZ	A randomized controlled phase 3 COVACTA trial failed to meet its primary endpoint of improved clinical status and did not improve patient mortality, but TCZ-treated patients spent approximately a week less in hospital as compared with the placebo group. The broad eligibility criteria COVACTA did not appear to stratify patients by clinical signs of hyperinflammation, which could have an impact on the responsiveness of the patients to the drug [192] (NCT04320615). In a prospective, open-label, randomized clinical trial of hospitalized patients with COVID-19 pneumonia in Italy, TCZ was not found to be significantly better than the control standard care at preventing the patients from deteriorating [193] (NCT04346355). A randomized, double-blind, placebo-controlled trial of hospitalized COVID-19 patients found TCZ was not effective for preventing intubation or death in moderately ill patients [194] (NCT04356937). An ongoing international trial of critically ill COVID-19 patients receiving organ support in intensive care treated with TCZ improved survival [191] (NCT02735707).
Umifenovir (Arbidol hydrochloride)	In a randomized controlled trial, arbidol monotherapy treatment of mild-to-moderate COVID-19 patients did not significantly improve recovery time [183] (NCT04252885).

### Emergency Use Authorization for HCQ/CQ and RDV

Early in the pandemic, hydroxychloroquine (HCQ) was given Emergency Use Authorization (EUA) for COVID-19 in April 2020 [195] (https://www.fda.gov/media/136537/download) which was subsequently revoked due to serious adverse cardiac events (https://www.fda.gov/media/136784/download) (https://www.fda.gov/media/138945/download). Indeed, both chloroquine (CQ) and HCQ, still undergoing clinical trials for COVID-19, had some studies that assessed for adverse cardiovascular complications or cardiac toxicity by monitoring QT interval prolongation or torsades de pointes. Subsequently, RDV was approved for EUA for COVID-19 in May 2020 (https://www.fda.gov/media/137564/download). Based on studies that RDV lowers viral load in animals [196, 197] and inhibits SARS-CoV-2 infection in cells [197], and a clinical trial (NCT04280705) found RDV was superior to placebo in shortening recovery time of hospitalized adult COVID-19 patients [189], there is ample support for the use of RDV for COVID-19. Nonetheless, another clinical trial (NCT04257656) found hospital patients with severe COVID-19 treated with RDV was not associated with statistically significant clinical benefits [190]. In addition, search for the effect of these drugs on the various organs identified an instance of torsades de pointes in a COVID-19 patient treated with RDV and requiring resuscitation [198], although it is unclear if this was due directly to RDV. Many clinical trials use QT interval threshold as part of the exclusion criteria, and while a number of drugs (e.g., AZM, baricitinib, CM, colchicine, corticosteroid, HCQ, ivermectin, LPV/RTV, sarilumab, TCZ) monitor for QT prolongation or cardiac arrhythmia as a secondary measure (NCT04381936, NCT04366206, NCT04382625, NCT04374019), trials with RDV to date do not appear to. In light of the comorbidities of COVID-19 patients, it would be beneficial to measure QT interval or other cardiac function as part of the measures and outcomes in the clinical trials of drugs for COVID-19.

### The Effect of CQ and RDV on 3D Human Heart Organoids

A retrospective study that analyzed electrocardiograms from 524 COVID-19 patients showed approximately 20% showed QT prolongation [199]. Therefore, it is critical that the drugs used to treat COVID-19 do not enhance the potential of QT prolongation. Although it is known that CQ has a potential for QT interval prolongation and was found to prolong QT interval in COVID-19 patients [200], less is known about RDV. Of the 78 clinical trials in Clinicaltrial.gov with RDV, none are explicitly monitoring for QT prolongation or torsades des pointes. While we were performing this study, another group assessed for cardiotoxicity and QT prolongation of CQ-treated and RDV-treated human iPSC-derived cardiomyocytes [20] and found that RDV is associated with both cardiotoxicity and arrhythmogenic risk. Although iPSC-derived cardiomyocytes constitute an excellent model for human cardiotoxicity studies [201–203], recent advances in stem cell technologies have facilitated the emergence of human stem cell–derived organ-like model systems (organoids) which allow for higher degree of precision and physiological relevance [204, 205]. Organoids recapitulate many organ properties, structure, and physiology to a significant extent, thus arguably constituting a better model than traditional 2D cell cultures containing a single cell type and no microenvironment [206]. In contrast, organoids have multiple cell types that interact with cardiomyocytes, along with matrix native to the heart, providing physicochemical properties that are better able to mimic the heart in vivo. Organoids are particularly useful to study unapproachable disease states in humans and have been used to model a wide range of tissues and disease conditions with great success [206, 207], including SARS-CoV-2 infection of human lungs and intestine [51, 205, 208]. Using a recently developed protocol for the generation of highly sophisticated human heart organoids (hHOs) from hiPSCs [209], the cardiac effects of CQ and RDV were explored with hHOs. Given the higher complexity in the organoids, including different cell types, morphology, and extracellular matrix, we expected the organoids will be more robust than cardiac monolayer cultures.

**Fig. 2:**
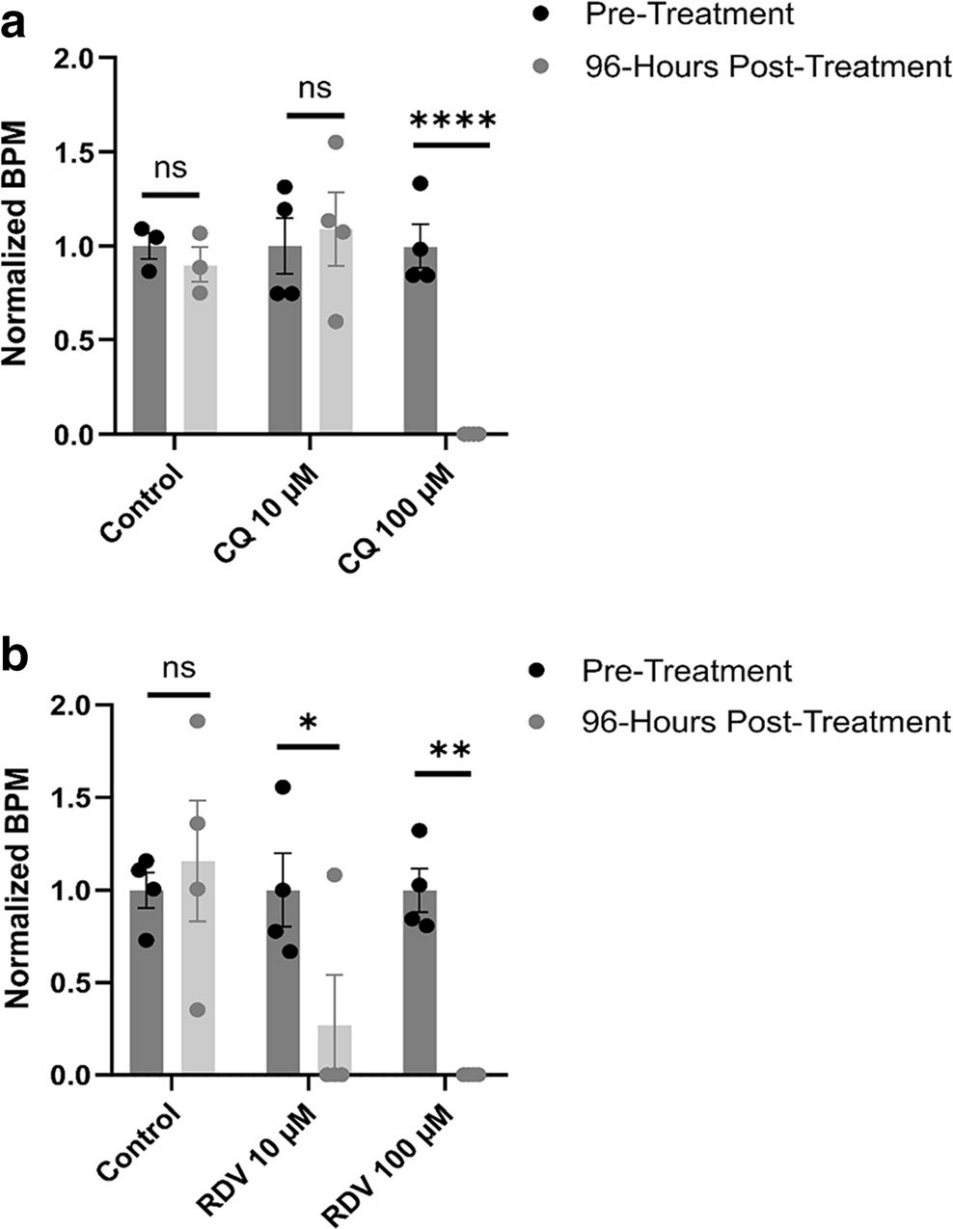
Chloroquine (CQ) and remdesivir (RDV) influence human heart organoid beat rate. Beat rate (per minute) was assessed following 96 h of treatment. Heart organoids were treated with either **a** CQ (*n* = 3, control; *n* = 4, 10 μM and 100 μM) or **b** RDV (*n* = 4 for all conditions) at concentrations of 10 μM or 100 μM for 96 h. Beats per min (BPM) in the treatment conditions were normalized to BPM in the pre-treatment condition for each individual organoid in each condition. (Value = mean ± s.d., two-way ANOVA multiple comparison test; *p = 0.0571, **p < 0.01, ****p < 0.0001)

Experiments were performed on hHOs derived from human iPSCs treated with CQ (known to have adverse cardiac effects) or RDV at two different concentrations to assess a potential of RDV for adverse cardiac effect. CQ and RDV were prepared as described in the methods (see supplementary file). At 96-h post-treatment with control media, CQ-containing media, or RDV-containing media, all organoids treated with control media or 10 μM of CQ were beating while 25% of the hHOs treated with 10 μM of RDV were beating. None of the heart organoids treated at the higher concentration (100 μM) of either CQ or RDV were beating (Fig. 2).

**Fig. 3:**
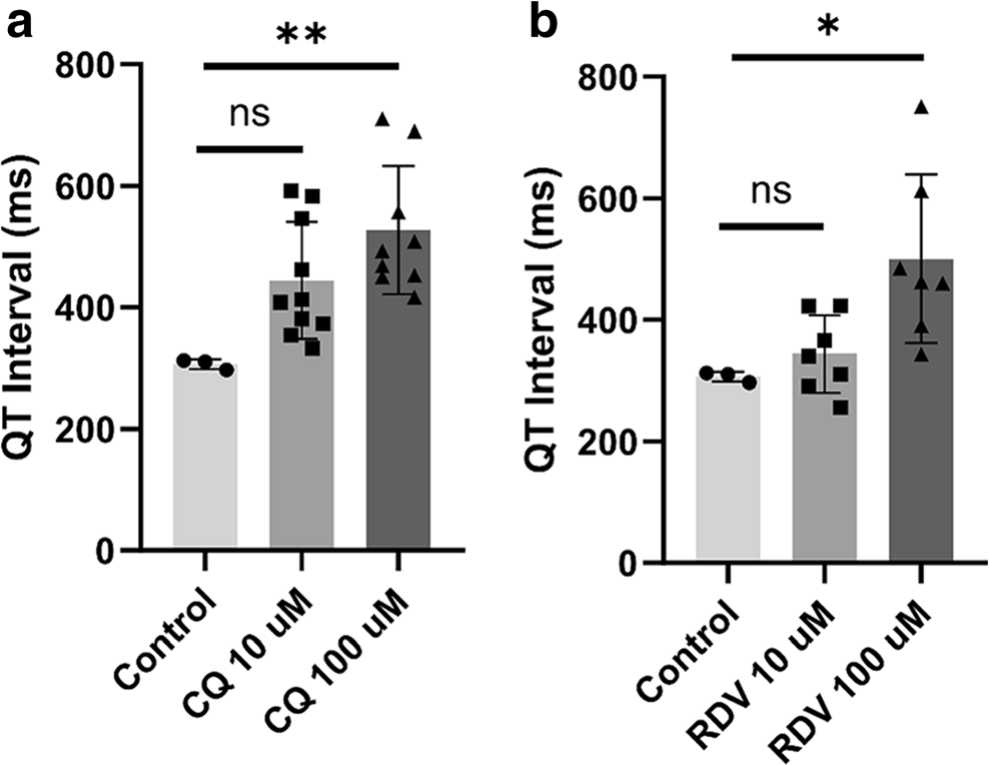
CQ and RDV induce QT interval prolongation in human heart organoids.Using the electrophysiological data obtained with the MEA system, QT intervals were measured in organoids with or without treatment of **a** CQ or **b** RDV. (value = mean ± SEM, one-way ANOVA multiple comparison test, compared to control; *p < 0.05, ** *p* < 0.005)

To assess for QT interval abnormalities in the hHOs, an in-house microelectrode array (MEA) system was used to record electrical activity of individual organoids. The QT interval in the control condition was 306 ± 4.70 ms. The QT intervals were heightened for both the 10 μM CQ and 10 μM RDV-treated hHOs, albeit non-significantly (444 ± 30.5 ms and 344 ± 24.2 ms, respectively) (Fig. 3). Notably, both 100 μM CQ and 100 μM RDV induced significant QT prolongation in the hHOs (527 ± 35.2 ms and 501 ± 52.5 ms, respectively). CQ was shown to exhibit an increased effect on QT prolongation as compared to RDV. Thus, the electrophysiological abnormalities arising from CQ and RDV treatment indicate a cardiotoxic mechanism in both CQ and RDV.

CQ and RDV both exhibit a pathological phenotype in the treatment of hHOs. While CQ induced cessation of visible beating at 100 μM, RDV exerted a similar effect at both 10 μM and 100 μM, suggestive of cardiotoxicity. These data suggest that further studies are needed to determine the safety and efficacy of RDV on the heart in the treatment of COVID-19.

### Limitations of Study

Therapies not covered in this review include stem cell therapy [210, 211], transfusion of convalescent plasma [212], and engineered decoys for neutralizing pathogens (including hACE2 for SARS-CoV-2) [213]. Although pilot studies [214–217] suggest that convalescent plasma could be beneficial, a randomized controlled study and an open-label, multicenter, randomized clinical trial did not find it to be associated with a reduction in the progression to severe COVID-19 or result in a statistically significant reduction in time to clinical improvement [218, 219], while early results of a clinical trial suggests it is safe and efficacious [220, 221]. This present study does not evaluate pharmacogenetics or how the genetic makeup of an individual contributes to their differential immune response to SARS-CoV-2 [222] or their differential response to a drug [223], which could aid in predicting which drugs affect an individual adversely while being beneficial to another.

## Conclusion

Even as vaccines are becoming available for COVID-19, variants of the SARS-Cov-2 virus are proliferating, leading to concerns that some mutations may reduce the efficacy of the vaccines to stem the broader pandemic [224, 225]. Therefore, treatments will continue to be an essential aspect in the fight against COVID-19 and its variants. Innovative repurposing of drugs, such as recent reports of the use of sarilumab and TCZ, are showing promise in the treatment of patients with severe COVID-19 [191]. Still, clinical trials are needed to assess the effects of these drugs on non-targeted organs and systems, such as the cardiovascular system. This study has compiled an extensive report of the many drugs proposed to treat COVID-19 and improve lung performance, along with examination of the effects of these drugs on other systems. Furthermore, the findings of this study are relevant to diseases other than COVID-19 by providing indications of how these drugs affect various organs. This could assist in guiding the implementation of these drugs in their repositioning for established and emerging diseases.

## Supplementary Information

ESM 1 (PDF 678 kb)

## Data Availability

Available upon request.

## Drugs:

AA: Ascorbic acid
AZM: Azithromycin
CM: Camostat mesilate
CQ: Chloroquine
DEX: Dexamethasone
DS: Disulfiram
DSTAT: Dociparstat sodium aka 2-0, 3-0 Desulfated Heparin (ODSH)
EVT: Entecavir
EPO: Epoprostenol
HCQ: Hydroxychloroquine
LPV: Lopinavir
MP: Methylprednisolone
NM: Nafamostat
NTZ: Nitazoxanide
NO: Nitric oxide
rhACE2: Recombinant human angiotensin-converting enzyme 2
RDV: Remdesivir
RTV: Ritonavir
RUX: Ruxolitinib
TAF: Tenofovir alafenamide
TDF: Tenofovir disoproxil fumarate
TET: Tetrandrine
THD: Thalidomide
TCZ: Tocilizumab
AZT: Zidovudine

## Other acronyms:

ARDS: Acute respiratory distress syndrome
ANG: Angiotensin
ANP: Atrial natriuretic peptide
CCCR#: C-C chemokine receptor #
CRP: C-reactive protein
CKD: Chronic kidney disease
CLL: Chronic lymphocytic leukemia
COPD: Chronic obstructive pulmonary
CHF: Congestive heart failure
CRS/CSS: Cytokine release syndrome/cytokine storm syndrome
CMV: Cytomegalovirus
ER: Endoplasmic reticulum
eNOS: Endothelial nitric oxide synthase
EMT: Epithelial-mesenchymal transition
HBV: Hepatitis B virus
HCV: Hepatitis C virus
HDL: High-density lipoproteins
HMGB1: High-mobility group box 1
HIV: Human immunodeficiency virus
iPSC: Induced pluripotent stem cell
iNOS: Inducible nitric oxide synthase
IFN: Interferon
IRF: Interferon regulatory factor
IL#: Interleukin #
JAK: Janus kinase
LPD: Lipopolysaccharide
LDL: Low-density lipoproteins
mTOR: Mammalian target of rapamycin
MAP 2: Microtubule-associated protein 2
MNC: Mononuclear cells
MS: Multiple sclerosis
MF: Myelofibrosis
NF-κB: Nuclear factor κB
NTP: Nucleoside triphosphate
NRTIs: Nucleoside/nucleotide reverse transcriptase inhibitors
PGI2: Prostacyclin
ROS: Reactive oxygen species
RA: Rheumatoid arthritis
SARS: Severe acute respiratory syndrome
SBECD: Sulfobutylether-β-cyclodextrin
SLE: Systemic lupus erythematosus
TLRs: Toll-like receptors
TGF-β: Transforming growth factor beta
TNF-α: Tumor necrosis factor alpha
