# Supplementary material for: Repositioned Drugs for COVID-19—the Impact on Multiple Organs
Source: SN Compr Clin Med. 2021 Apr 21;3(7):1484–501. doi: 10.1007/s42399-021-00874-8 (PMC8057921; doi:10.1007/s42399-021-00874-8)
Supplement: Supplementary file 1 — (PDF 678 kb) [file 42399_2021_874_MOESM1_ESM.pdf]

Supplementary File

Materials and Methods:

Human heart organoid (hHO) generation

iPSCs were plated in ultra-low attachment 96-well plates with Essential 8 Flex medium and 2 μM thiazovivan (Millipore Sigma) at a density of 10,000 cells per well and centrifuged for 3 minutes at 100g to form spherical aggregates. Following a 48-hour incubation at 37°C and 5% CO2, two-thirds of medium was removed and replaced with fresh RPMI 1640 (Gibco) with B27 supplement minus insulin including CHIR99021 (Selleck) (4 μM), Activin A (1.25 ng/ml), and BMP4 (1 ng/ml). After 24 hours, media was changed with RPMI/B-27 minus insulin. Following an additional 24 hours, media was changed with RPMI/B-27 minus insulin including Wnt-C59 (Selleck) (2 μM) and incubated for 48 hours. Following this, the media was changed with RPMI/B-27 minus insulin. After an additional 48 hours, media was changed to RPMI/B-27 (Gibco). After 24 hours, media was changed with RPMI/B-27 including 4 μM CHIR99021 for 1 hour, then the media was changed with RPMI/B-27. Following this, the organoids were allowed to culture for an additional 13 days, with 48-hour media changes occurring hereafter. After 13 days, the organoid medium was permanently switched to RPMI/B-27 including oleic acid, linoleic acid, T3 hormone, L-carnitine, and palmitic acid (Thermo Fisher). Following an additional 10 days in culture, the organoids were treated with CQ and RDV.

Culturing of hHO

CQ and RDV were prepared to achieve 10 μM and 100 μM final concentrations in the organoid wells. The hHOs were cultured in drug-containing media for 96 hrs, media replenished after 48 hours.

Multi-Electrode Array

Organoids were placed on top of the MEA within a 3D printed PDMS well, in 20 μL culture media. The MEA was placed within a Faraday cage inside an incubator at 37°C at low humidity to avoid damage to the MEA system. Each organoid was placed and kept recording in the incubator firstly for 20 minutes and then 20 μL isoproterenol was added (1 μM final concentration) to record for a period of 10 minutes. The PDMS well was washed with PBS between organoids for three times. The recorded signals were amplified and digitalized using a commercial Intan RHD2132 system (Intan Technologies) and then analyzed using the Matlab Chronux toolbox [1]. Representative electrical waveforms were generated for each condition following a 1μM isoproterenol challenge. This data was used to measure the QT intervals of individual hHO.

Supplementary Table 1: Drug Classification

Drug	Drug Classification
Abacavir	Nucleoside reverse transcriptase inhibitor (NRTI) - antiviral
Anakinra	Interleukin 1 receptor antagonist
APN01	Recombinant ACE2 (rhACE2)
Ascorbic Acid	Vitamin C
Azithromycin	Antibiotic
Baloxavir	A cap-endonuclease inhibitor - antiviral
Barictinib	JAK1/2 inhibitor
Brincidofovir/ Cidofovir	A cyclic cytidine nucleotide analogue (CMX001) - antiviral
Camostat Mesylate	Serine protease inhibitor
Chloroquine	Aminoquinoline, antimalarials and amebicides
Colchicine	Plant alkaloid, anti-gout agents, immunosuppressant
Corticosteroids	Synthetic steroid hormone
Dexamethasone	Synthetic glucocorticoids (steroid hormone)
Disulfiram	Antabuse, inhibits aldehyde dehydrogenase (ALDH1A1), proteasome inhibitor
Dociparstat Sodium	Glycosaminoglycan derivative of heparin, CXCL12/CXCR4 inhibitor (CX-01)
Emtricitabine	Nucleoside reverse transcriptase inhibitors (NRTIs) - antiviral
Entecavir	Nucleoside reverse transcriptase inhibitors (NRTIs) - antiviral
Epoprostenol	Member of family of prostaglandins, vasodilator
Famotidine	Antihistamine and Antacid, H2 receptor antagonist
Favipiravir	Pyrazinecarboxamide derivative - antiviral against RNA viruses
Hydroxychloroquine	Aminoquinoline, antimalarials, amebicides, antirheumatic agents
IC14	Anti-CD14 monoclonal antibody
Idelalisib	ATP-competitive kinase inhibitor that targets the PI3K
IFN-α	Cytokine
Ivermectin	Anthelmintics
Leronlimab	CCR5 blocking monoclonal antibody
Lopinavir-Ritonavir	Antiviral protease inhibitors
MethylPrednisalone	Glucocorticoid
Nafamostat	Serine protease inhibitor
Niclosamide	Anthelmintics
Nitazoxanide	Antiprotozoal and antihelmintic agent, anti-parasite drug
Nitric Oxide	Vasodilator
Remdesivir	Adenosine triphosphate analog - antiviral
Ruxolitinib	JAK1/2 inhibitor, antineoplastic
Sarilumab	Antirheumatic, monoclonal antibody against IL-6 receptor antagonist
Sirolimus (Rapamycin)	MTOR inhibitor, antineoplastic, immunosuppressant
Sofosbuvir	Nucleotide HCV NS5B polymerase inhibitor - antiviral
Stavudine	Nucleoside reverse transcriptase inhibitor (NRTI) - anitiviral
Statins	HMG-CoA reductase inhibitors, immunosuppressant
Tenofovir Alefenomide/ Tenofovir Disoproxil	Nucleoside reverse transcriptase inhibitors (NRTIs) - antiviral
Tetrandrine	Calcium channel blocker
Thalidomide	Glutamic acid derivative, immunonodulator and antineoplastic
Tocilizumab	Monoclonal antibody IL-6 receptor inhibitor
Umifenovir (Arbidol hydrochloride)	Indole-based, hydrophobic, dual-acting direct antiviral/host-targeting agent
Valganciclovir/ Ganciclovir	Synthetic analog of 2'-deoxy-guanosine, nucleoside analogue, antiviral
Zidovudine	Nucleoside reverse transcriptase inhibitors (NRTIs) - antiviral

Supplementary Table 2: Drug Effects on the Immune System

Drugs	Immune System
Abacavir	**Negative:** In HIV-positive patients hypersensitive to abacavir, an increased level of inflammatory and type 1 cytokines were observed as compared to the control [2].
Anakinra	**Positive:** Anakinra is a human recombinant IL-1 receptor antagonist used to treat RA patients. It blocks the activity of IL-1 by binding to IL-1R type I. In combination with methotrexate serum levels of IL-17, IFN-γ, IL-21 and IL-1β decreased significantly compared to methotrexate treatment alone [3].<br>Anakinra used to treat moderate to severe COVID-19 pneumonia was found to reduce blood inflammation markers and CRP [4, 5].
APN01 (rhACE2)	**Positive:** Reduces inflammatory cytokines such as IL-6 and has potential to neutralize the virus and protect the lungs [6]. The virus binds to APN01 as a decoy of ACE2 [7], inhibiting infection of engineered human tissue [8].<br>A case study of a patient with severe COVID-19 given 7-day administration of rhACE2 reduced concentrations of critical cytokines implicated in COVID-19 pathology (IL-6, IL-8, TNF-α), ferritin, and CRP [9].
Ascorbic Acid (AA)	**Positive:** Vitamin C (AA) acid supports the immune system through various cellular functions of both the innate and adaptive immune system [10]. It selectively influences intracytoplasmic cytokine production and inhibits IL-6 and TNF-α production by LPS-induced monocytes [11].
Azithromycin (AZM)	**Positive:** AZM modulates MAPK kinase activity to inhibit cytokine secretion and inflammation [12, 13].
Baloxavir	**Positive:** Used to treat influenza by blocking initiation of viral mRNA synthesis, and thereby preventing proliferation of the influenza virus [14].<br>A double-blind, placebo-controlled and oseltamivir-controlled trial in outpatients aged 12 years and older found single-dose baloxavir has superior efficacy to placebo and similar efficacy to oseltamivir for ameliorating influenza symptom [15].
Baricitinib	**Positive:** Baricitinib used to treat human monocyte-derived dendritic cells, plasmacytoid dendritic cells, B cells, and T cells, reduced the production of IFN and IL-6, and suppressed the differentiation of plasmablasts, T helper (Th) type 1 and Th17 cells, and innate immunity [16].<br>Off-label use of baricitinib for severe COVID-19 reduced serum cytokine levels (IL-6, IL-1β, and TNF-α) [17].<br><br>**Negative:** Baricitinib has known to cause neutropenia and lymphocytopenia [18].
Brincidofovir/ Cidofovir	**Positive:** Has been shown to have activity against several different viruses [19].<br>CXM001 rescued an immunosuppressed hamster model challenged with lethal dose of adenoviruses [20].
Camostat Mesylate (CM)	**Positive:** Human tracheal epithelial cell culture treated with CM reduced influenza viral replication and the production of inflammatory IL-6 and TNF-a cytokines [21].

Chloroquine (CQ)	See also HCQ:<br>**Positive:** Used to treat RA, SLE. The drug has immunomodulatory effects, including inhibition of cytokine production and immune activation [22, 23].
Colchicine	**Positive:** Exerts broad anti-inflammatory and immunomodulatory effects by modulating innate immunity, and inhibiting neutrophil, and NALP3 inflammasome activation [24].<br>In this prospective, open-label, randomized and double blind clinical trial of COVID-19 patients found colchicine added to HCQ was effective in reducing inflammatory markers as compared to HCQ alone [25].<br><br>**Neutral:** In an open-label, randomized clinical trial for COVID-19 there was no significant differences in CRP between colchicine-treated and standard care patients [26].
Corticosteroid	**Positive:** Potent anti-inflammatory and antifibrotic properties, may prevent an extended cytokine response and may accelerate resolution of pulmonary and systemic inflammation in mechanically ventilated patients with severe pneumonia [27].
Dexamethasone (DEX)	**Positive:** DEX selectively inhibits IL-12-induced Stat4 phosphorylation in T Lymphocytes. Preventing Stat4 phosphorylation decreases IL-12 responsiveness and diminishes the induction of IFN-gamma and IRF-1 [28].<br>DEX is a potent anti-inflammatory and antifibrotic properties, may prevent an extended cytokine response and may accelerate resolution of pulmonary and systemic inflammation in pneumonia [29].
Disulfiram (DS)	**Positive:** DS helped ameliorate immune system function in alcoholics including reducing adrenocorticotropic hormone (ACTH) and cortisol levels, and restoring TNF and IFN-gamma levels [30].<br>It blocked cytokine release and pyroptosis in LPS-primed human THP-1 cells and suppressed LPS-induced sepsis in mice [31].
Dociparstat Sodium (DSTAT, aka ODSH)	**Positive:** CXCL12/CXCR4 have an impact on the immune and nervous system [32]. Their signaling may be involved in resistance of some cancers to immunotherapies, inhibiting this pathway could contribute to anti-cancer activities [33].<br>DSTAT has broad anti-inflammatory activities [34] and inhibits the secretion of proinflammatory cytokines, interleukin (IL)-1beta and IL-6 in a rat burn model [35].<br>On a related heparin compound, a retrospective analysis on low molecular weight heparin (LMWH) treatment found the levels of IL-6 in the LMWH group significantly lower than in the control group [36].
Emtricitabine	**Positive:** TDF combined with emtricitabine decreased T-cell activation but IL-10, IFN-γ and TNF-α levels were not altered [37].<br>Low long-term toxicity in combination with TAF [38].
Entecavir (EVT)	**Positive:** Chronic active hepatitis B patients treated with EVT reduced levels of IL-37, which has been identified as a potent inhibitor of the immune response [39].<br><br>**Negative:** EVT did not influence TGF-β, IL-9, IFN-γ, and TNF-α production but reduced the level of IL-10 (which is an anti-inflammatory cytokine) [40].<br>EVT's adverse effects include upper respiratory tract infection, fatigue, and lactic acidosis [41].
Epoprostenol	**Positive:** Generally well tolerated for long-term use in patients with hypertension [42].

(EPO, synthetic equivalent of prostacyclin)	**Mixed:** EPO is prostaglandin I2. PG12 (prostaglandin I2 or PGI2) has different effects depending on the disease (promotes disease progress in RA ("pro-inflammatory"), and inhibits disease progression with pulmonary vascular disease and atherosclerosis ("anti-inflammatory") [43].
Famotidine	**Positive:** Famotidine inhibits HIV replication. COVID-19 patients receiving famotidine displayed lower levels of serum markers for severe disease including lower median peak CRP levels [44].<br><br>**Neutral:** Famotidine did not have as strong an immunomodulating effect (e.g. soluble interleukin-2 receptor production) as other histamine-2 receptor antagonists like cimetidine in gastric cancer patients [45].
Favipiravir	**Positive:** Favipiravir is a novel antiviral drug now in clinical trials for the treatment of influenza (1400 subjects have been involved with clinical trials in several countries). Favipiravir inhibits the RNA polymerase of many different RNA viruses and blocks replication of many strains of influenza virus [46] as well as Ebola virus in cynomolgus macaques [47] and mice. In mice, it was suggested that the drug's suppression of viral replication of Ebola allows the host to mount a virus-specific adaptive immune response [48].<br>A prospective, single-center study of hospitalized severe COVID-19 patients requiring mechanical ventilation with delayed treatment of favipiravir had some benefits but found inflammation and cytokine storm continued, requiring steroids to controlled them [49].<br><br>**Negative:** A case study of a heart transplant patient who contracted COVID-19 dropped lymphocyte count during favipiravir treatment [50].
Hydroxy Chloroquine (HCQ)	**Positive:** Used in treating RA, SLE. The drug has immunomodulatory effects, including inhibition of cytokine production and immune activation [22, 23].
IC14	**Positive:** It targets the immune system protein CD14 and reduces systemic inflammatory response induced by LPS [51] and could be a potential treatment against organ dysfunction in severe sepsis [52].
Idelalisib	**Negative:** In patients treated for CLL, idelalisib can cause immune-mediated severe hepatotoxicity (recorded in 54% of patients). Toxicity was lower if patients were receiving steroids at the time [53]. Evidence points to a possible autoimmune mechanism as the source of the toxicity [53]. Idelalisib impairs T-cell-mediated immunity in CLL and this impairment induced by idelalisib favored infections and/or viral reactivations [54].
IFN-α	**Positive:** IFN-α has an essential role in the innate cytokine response to viral infection [55]. Initially used as an antiviral therapy but has tumor suppression abilities [56].<br>In COVID-19 patients in one hospital treated with IFN-α2b significantly reduced duration of elevated blood levels of IL-6 and CRP [57].<br><br>**Mixed:** IFN-α can induce as well as inhibit the expression of several interleukins cytokines and cytokine receptors (IL-1, IL-2, IL-6, IL-8, IL-1 receptor, IL-1 receptor antagonist, TNF, TNFreceptor, and IFN-gamma) [58].
Ivermectin	**Positive:** Ivermectin is known to be immunostimulatory for parasitic control and could be beneficial for treating diseases involving immunosuppression [59, 60].<br>Ivermectin inhibits SARS-CoV-2 replication in vitro [61].

Leronlimab (PRO 140)	**Positive:** Critically ill COVID-19 patients treated with leronlimab reduced plasma IL-6, restored CD4/CD8 ratio, inhibited CCR5, and decreased SARS-CoV-2 plasma viremia [62].
Lopinavir/ Ritonavir (LPV/RTV)	**Negative:** LPV induced inflammatory cytokine **(**IL-6 and TNF-α) synthesis mediated by the unfolded protein response (UPR)/ER stress and extracellular-signal regulated protein kinase (ERK) activation in macrophages [63].
Methyl Prednisolone (MP)	**Positive:** Potent anti-inflammatory and antifibrotic properties, may prevent an extended cytokine response and may accelerate resolution of pulmonary and systemic inflammation in pneumonia. MP treatment of RA patients unresponsive to conventional therapy reduced inflammation and improved clinical parameters [64].<br>A case study of COVID-19 patients with ARDS treated early with high-dose, short-term systemic MP reduced CRP levels [65].
Nafamostat (NM)	**Positive:** NM has immunomodulatory effects on ischemic stroke in rats. This is related to the suppression of the NLRP3 inflammasome and NF-κB signaling pathways. It also reduced the level of IL-1β, TNF-α, COX-2, and iNOS but increased the expression of TGF-β, CD206, IL-4, and IL-10 [66].<br>NM treatment prior to reperfusion in an animal study inhibited complement pathway and had an antiinflammatory effect. [67].
Niclosamide	**Positive:** Niclosamide has anti-inflammatory effect in RA. It reduced cytokine secretion in a cell model of RA and also reduced the severity of collagen-induced arthritis (CIA) in a rat model [68]. Cytokine storm during influenza virus infection can contribute to morbidity and mortality and blocking IL-1β reduces influenza-induced inflammation [69].<br><br>**Mixed:** Nicolsamide also was found to activate the NLRP3 inflammasome and release the proinflammatory cytokine, IL-1β in macrophage**s** [70]. In an IL-1 KO animal study, IL-1 was found to promote clearance of influenza virus and protects from virus-induced mortality [71].
Nitazoxanide	**Positive:** In a study of mice treated with NTZ, it was found to inhibit the production of pro-inflammatory cytokines in peripheral blood MNC and suppressed IL-6 production [72]. Inhibits replication of canine, human, and mouse coronaviruses in vitro, including MERS [72].<br>NTZ has been shown to have immunomodulating effects on healthy patients [73].<br>It was found to amplify the host innate immune response to viruses and inhibited Ebola virus replication [74].
Nitric Oxide (NO)	**Mixed:** Has many effects on the immune system and shapes the immune response intra- and intercellularly. The production of NO by means of iNOS is detrimental in some infectious disease processes, however it can also take part in counteracting excessive immune reactions, and somewhat protects against autoimmunity [75].
Remdesivir (RDV)	**Positive:** RDV is a nucleotide polymerase inhibitor, reduced Ebola viral replication in non-human primates (NHPs) [76]. GS-5734 (RDV) when administered intravenously in healthy NHPs resulted in rapid accumulation and persistence of intracellular NTP, which acts as an alternative substrate and RNA-chain terminator [77].<br>RDV has been shown to have better antiviral activity than LPV and RTV for treating MER-CoV infection [78].<br>A COVID19 patient treated with the addition of RDV to TCZ showed marked improvement, suggesting that administering RDV early before secondary uncontrolled inflammatory response would be beneficial [79].

Ruxolitinib (RUX)	**Positive:** It was able to prevent CRS without impairing the anti-tumor effect of CAR T-cells [80].<br>A prospective, multicenter, controlled, randomized, single-blind study of patients with severe COVID19 treated with RUX was not associated with significantly accelerated clinical improvement as compared to control group but the levels of IL-12 and IL-6 cytokines and high-sensitivity CRP were significantly reduced in the RUX group as compared to the control group [81].<br>A monocentric retrospective chart analysis used a COVID19 inflammation score for imminent or full blown hyperinflammation found RUX treated patients significantly reduced their inflammation score with sustained clinical improvement [82].<br><br>**Negative:** When used for treating MF, most common adverse effects include anemia and thrombocytopenia but they rarely led to discontinuation of the drug [83].
Sarilumab	**Positive:** Sarilumab, developed originally to treat RA, is an IL-6 receptor antagonist. IL-6 is implicated in RA [84]. Sarilumab used as an RA treatment has a stable safety profile [85-87].<br>In a retrospective study, patients with lower levels of IL-6 and neutrophil to lymphocyte ratio at baseline responded to sarilumab with rapid decreases in CRP level and positive clinical outcome, but was limited by study size [88].<br><br>**Negative:** In treating moderate-to-severe RA patients, sarilumab reduced CRP levels but neutropenia was reported with some patients as with other IL-6Rα inhibitors. Most common adverse event was infections [89].
Sirolimus (Rapamycin)	**Positive:** Sirolimus has immunosuppressive activity on CD4+ effector T cells [90], and is a key regulator of the innate immune response [91] and also has a stimulatory effect on the adaptive immune system [92].
Sofosbuvir	**Positive:** Sofosbuvir in combination with ribavirin provided sustained virological response in HCV patients. Adverse effects include fatigue and myalgia [93].
Statins	**Positive:** This lipid lowering agent has immunosuppressive function. It inhibited induction of major histocompatibility complex class II (MHC-II) expression by IFN-γ [94].<br>Statins reduced IL-6 production in human vascular smooth muscle cell and human mononuclear cell cocultures [95].<br>Long-term use of statins is associated with inhibition of the progression of aortic stenosis and aortic stiffness [96, 97].<br>An observational study of COVID-19 patients with diabetes a statin had lower inflammatory markers (CRP) and reduced cumulative in-hospital mortality than those not on a statin. No difference in hospital mortality was observed in non-diabetic patients on or off statin.
Stavudine	**Positive:** Stavudine increased HIV-specific type 1 cytokine production, which include IL-2, IL-7, IL-15, and CD4 counts and reduced plasma viremia [98].<br>**Negative:** Stavudine has been shown to increase cytokine secretion in mouse adipocytes and preadipocytes [99] and associated with lipoatrophy [100].
Tenofovir	**Mixed:** TAF modulates cytokine production (e.g. IL-8), although the results are mixed with studies showing both upregulation and downregulation of cytokine levels [101, 102].<br>TDF has low long-term toxicity in combination with emtricitabine [38].
Tetrandrine (TET)	**Positive:** TET inhibits proinflammatory helper T cells Th1, Th2, and Th17 in mice study [103].<br>Th17 cells play an important pathogenic role in autoimmunity [104], and is an important effector in

	the development of autoimmune diseases (e.g., multiple sclerosis, RA, respiratory disease, SLE, psoriasis, systemic sclerosis and chronic inflammatory bowel disease) [105].<br>One study showed TET suppressed replication of HCoV-OC43 and reduced inflammatory cytokine production in MRC-5 (human lung) cells. The drug did not result in increased levels of cytotoxicity at the effective dosage levels [106]. Cytokines are elevated and involved in Th17 responses in many patients with COVID-19 and are associated with cytokine storm [107, 108], thus TET could be beneficial.
Thalidomide (THD)	**Positive:** An immunomodulatory drug with antiangiogenic and anti-inflammatory effects [109].<br>Has been shown to be anti-inflammatory and inhibit TNF-α in human monocytes [110].<br>Is also known to have anti-oncogenic properties by inhibiting angiogenesis [111, 112].<br>Some studies have shown a decrease in TNF-α level with THD treatment for erythema nodosum leprosum [113] whereas with myelodysplastic syndromes THD did not significantly decrease TNF-α level [114].<br><br>**Mixed:** THD has both anti-inflammatory and proinflammatory effects [115]. There are contradictory results reported concerning its effects on cytokine levels in vivo.
Tocilizumab (TCZ)	**Positive:** Used to treat cytokine storms caused by certain cancer treatments [116]. TCZ has been found to be an effective treatment for CRS in leukemia [117, 118]. TCZ binds IL-6 receptors to block IL-6-mediated responses in human IL-6R expressing COS-7 cells and suppresses the growth of the IL-6-dependent myeloma cell line [119].<br>A systematic literature search of clinical studies with primary data on TCZ treatment for severe COVID-19 found TCZ lowered fatality as compared with standard of care [120].<br>In a retrospective analysis of medical records TCZ was found to improve inflammatory responses in critically ill COVID-19 patients [121].<br>In another retrospective, observational cohort study patients with severe COVID-19 needing support in the intensive care unit and receiving TCZ found patients with baseline CRP levels of 15 mg/dL or higher were most likely to show an associated improved survival with TCZ which was not observed in patients with lower levels of CRP [122].<br>A study on preemptive therapy with TCZ of severe COVID-19 patients identified predictors of responsiveness of patients to TCZ therapy. They found lower neutrophil/lymphocyte ratio and systolic blood pressure were associated with favorable outcome (lower mortality) with TCZ therapy, although a comparable control group was not included [123].<br><br>**Mixed:** An observational study of 239 COVID-19 patients with CRS found that following TCZ treatment oxygenation and inflammatory biomarkers (e.g., high-sensitivity CRP and IL-6 reduced, however, D-dimer and soluble IL-2 receptor (aka CD25) levels increased significantly [124].<br>Indeed, IL-6 is known to have both pro- and anti-inflammatory properties [125].<br><br>**Negative:** TCZ has also been associated with serious infections in RA-treated patients [126-128].
Umifenovir (Arbidol)	**Positive:** In a mice study, arbidol decreased secretion of TNF-α in hantaan virus infections [129].<br>Exhibits some immunomodulatory and anti-inflammatory effects (e.g. reducing cytokine production) [130, 131].<br><br>**Neutral:** A retrospective study of COVID-19 patients found umifenovir did not improve the prognosis or accelerate SARS-CoV-2 clearance in non-ICU patients [132].<br>In support, a systemic review and meta-analysis of umifenovir use for COVID-19 did not improve disease outcome. More research is needed to reveal the association of umifenovir, immunity of host

	and clearance of SARS-CoV-2 [133].
Valganciclovir/ Ganciclovir	**Positive:** Studies (both in vivo and in vitro) showed ganciclovir treatment inhibited the immune response typically observed in the presence of CMV infection in transplant patients [134].<br><br>**Negative:** Adverse effect include neutropenia [135].
Zidovudine (AZT)	**Negative:** AZT has been shown to increase cytokine secretion in mouse adipocytes and preadipocytes [99].<br>Long-term treatment with AZT may induce mitochondrial toxicity in HIV patients [136].<br>It may also induce anemia, leukopenia [137], myalgia, muscle weakness, and increased serum creatine kinase levels [136].

Supplementary Table 3: Drug Effects on the Lungs

Drugs	Lung Effects
Abacavir	**Positive:** A randomized double-blind trial of HIV patients with low CD4 starting antiviral therapy had lower rate of serious adverse reaction than with nevirapine [138].<br><br>**Neutral:** A cohort study did not find abacavir to affect the risk for COVID-19 diagnosis in HIV-positive patients [139].<br><br>**Negative:** Abacavir hypersensitivity and influenza have overlapping symptoms. Abacavir is associated with gastrointestinal (GI) symptoms, cough, and rash, while cough without GI symptoms is associated with influenza [140].
Anakinra	**Positive:** An immunosuppressive drug used to treat RA [141]. A retrospective cohort study suggests treating with anakinra increased mechanical ventilation-free survival over standard treatment of COVID-19 patients [142].<br>In a cohort study, it also reduced mortality among severe COVID-19 patients [143].<br>In a retrospective cohort study of COVID-19 patients with ARDS and exhibiting signs of hyperinflammation (cytokine storm syndrome) and treated with high doses of anakinra showed favorable outcome, with reduced recovery rate, similar to tocilizumab. But the study was limited by a small sample size and lack of a placebo group [144].
APN01 (rhACE2)	**Positive:** APN01 is a recombinant human ACE2. It inhibited SARS-CoV-2 infection of engineered human blood vessel and kidney organoids. It also inhibited SARS-CoV-2 infection in a dose dependent manner in vitro [8].<br>APN01 reduces the negative effects of acute inflammation in the lungs induced by SARS-CoV-2 infection and was found to decrease SARS-CoV-2 viral load in a case study [145].
Ascorbic Acid (AA)	**Positive:** In the immune system, AA is a physiological antioxidant, protecting host cells against oxidative stress caused by infections [146].<br>In lung fibrosis induced by paraquat, vitamin C reduced secretion of IL-17 (a cytokine that links T cell activation to neutrophil mobilization and activation) and TGF-β-a pro-fibrotic mediator. It enhanced the antioxidant enzymes superoxide dismutase (SOD) and catalase [147].<br>A meta-analysis of patients undergoing elective cardiac surgery found vitamin C reduced the length of stay in the intensive care unit (ICU) and duration of mechanical ventilation [148].
Azithromycin (AZM)	**Positive:** A meta-analysis of long-term use of AZM in cystic fibrosis patients improved lung function [149].<br>A randomized trial of AZM found decreased frequency of exacerbations for chronic obstructive pulmonary disease (COPD) [150].<br>AZM inhibited mucus secretion from airway epithelial cells in vitro, which could be useful for treating mucus hypersecretion caused by allergic inflammation and LPS stimulation [151].<br>In COPD, AZM treatment led to reduced chemokine (C-X-C) ligand 1 (CXCL1), tumor necrosis factor (TNF)-α, interleukin (IL)-13 and IL-12p40 levels in bronchoalveolar lavage (BAL), but increased bacterial metabolites including glycolic acid, indol-3-acetate, and linoleic acid [152].<br>A small, non-randomized French study found that HCQ in combination with AZM was associated with significantly reduced viral load as compared to HCQ alone for COVID-19 [153].<br>Similarly, an uncontrolled, non-comparative, observational cohort study of 80 relatively mildly infected inpatients suggest HCQ/AZM combination to be beneficial for COVID-19 [154].

	**Neutral:** In a randomized clinical trial AZM plus standard care did not result in a statistical difference in recovery time as compared to standard care alone which included HCQ [155].
Baloxavir	**Positive:** A randomized, controlled trial involving patients at high risk for influenza complications found baloxavir was better at alleviating influenza symptoms than placebo in patients with uncomplicated influenza, and better than the oseltamivir group in reducing the viral load [156].<br>Blocks initiation of viral mRNA synthesis, and thereby preventing proliferation of the influenza virus [157].<br>Effective in treating H7N9 in mice. Prevented virus replication in respiratory organs [158].<br>The drug also reduced virus titer, inflammatory response and mortality in a mouse model infected with influenza A virus [159].<br>It was found to partially inhibit SARS-CoV-2 infection in vitro (in VeroE6 cells) [160].<br><br>**Neutral:** An exploratory trial did not find baloxavir to have any viral effect or clinical benefit with COVID-19 patients [161].<br><br>**Negative:** A phase 3, double-blinded, placebo-controlled randomized clinical trial found that following baloxavir treatment there appeared to be an emergence of influenza virus with PA/I38X substitutions which was associated with transient rises in infectious virus titers, prolongation of virus detectability, initial delay in symptom alleviation [162].
Baricitinib	**Positive:** Use to treat RA. This inhibitor of JAK functions by inhibiting the activity of selective JAK1 and JAK2 enzymes, that interfere with the JAK-STAT signaling pathway [163]. Early indications suggest that baricitinib with HCQ and LPV/RTV reduced recovery time. A case study indicated favorable clinical outcome for a RA patient who underwent baricitinib therapy for more than a year and who developed COVID-19 [164].<br>A double-blinded, randomized, placebo-controlled COVID-19 trial found baricitinib plus RDV reduced recovery time and accelerated clinical status improvement over RDV alone [165].<br><br>**Negative:** Report of RA patients treated with baricitinib developed deep vein thrombosis (DVT)/ pulmonary embolism (PE) [163].
Brincidofovir/ Cidofovir	**Positive:** A case study found brincidofovir (CMX001) was effective in treating severe adenoviral pneumonia in an adult renal transplant recipient [166].<br>Another case report found brincidofovir successfully treated a hematopoietic stem cell transplant patient with disseminated adenovirus infection [167].<br>Cidofovir inhibits lung metastasis of FGF2-T-MAE (murine endothelial cells) in mice without affecting primary tumor growth. It also protected against lung metastasis of melanoma cells in zebrafish and mice [168].
Camostat Mesylate (CM)	**Positive:** Matriptase expression is increased in patients with idiopathic pulmonary fibrosis (IPF) and using CM for treating pancreatitis [169] in a murine model of bleomycin-induced pulmonary fibrosis, inhibited matriptase and reduced pulmonary fibrogenesis [170].<br>CM inhibits TMPRSS2 and the spread of the virus as well as protected the SARS-CoV infected mice from death [171].<br>Both TMPRSS2 and lysosomal cathepsins are involved in activating SARS-CoV-2 entry [172].<br>CM inhibited but did not abrogate infection of lung cells. The drug partially blocked the entry of the SARS-CoV-2 virus into cells. Full inhibition resulted when E-64d, an inhibitor of CatB/L, was added with camostat [173].

	**Negative:** There was a reported case of CM causing acute eosinophilic pneumonia [174].
Chloroquine (CQ)	**Positive:** CQ prevents spread of the SARS virus in cell culture and could be due to its ability to prevent glycosylation of ACE2 [175].<br>Using ACE2 high-expressing HEK293T cells, CQ and HCQ were effective in blocking the SARS-CoV-2 spike pseudotyped virus from binding the ACE2 receptor and entering the cells [176].<br><br>**Negative:** CQ does not block SARS-CoV-2 infection of the TMPRSS2-positive human lung cell line [177].<br>A systematic review and meta-analysis found that CQ (and HCQ) are not effective at reducing the short-term mortality of COVID-19 and their effects on viral clearance were mixed, leaning towards no effects [178].
Colchicine	**Positive:** Treating pulmonary fibrosis patients with colchicine decreased hydroxyproline level, contributing to a mild antifibrotic effect but there was no effect on inflammation (i.e., levels of cytokines) that accompanies pulmonary fibrosis [179].<br>A single-center randomized, double-blinded, placebo controlled clinical trial of colchicine for moderate to severe COVID-19 found the time of need for supplemental oxygen was reduced by half [180].<br><br>**Neutral:** A prospective study of patients with idiopathic interstitial pneumonia (UIP) (a subgroup of IPF) found colchicine did not result in improvement or stopped the progression of the disease. It was ineffective as an antifibrotic agent. However as compared with prednisone (standard care), it experienced a lower incidence of side effects as well as a trend towards less rapid decline of pulmonary function and death (albeit not statistically significant) [181].
Corticosteroid	**Positive:** Decreases in inflammatory cytokines, C-reactive protein, and inflammatory cells have been observed with corticosteroid use, suggesting possible benefits for chronic obstructive pulmonary disease (COPD) [182].<br>Corticosteroid therapy reduced mortality and morbidity in adults with severe community-acquired pneumonia and reduced morbidity, but not mortality, for non-severe community-acquired pneumonia but was associated with hyperglycaemia [183].<br>Among non-ICU patients hospitalized with COVID 19 pneumonia complicated by acute hypoxemic respiratory failure (AHRF), treatment with corticosteroid (methylprednisolone, prednisone, hydrocortisone, or DEX) was associated with a significantly lower risk of ICU transfer, intubation, or in hospital death [184].<br>In a prospective meta-analysis of randomized trials found corticosteroids was associated with lower 28-day all-cause mortality, as compared with usual care or placebo, in hospitalized, critically ill patients with COVID-19 [185].<br><br>**Negative:** Steroid toxicity and increased vulnerability to pneumonia and death are adverse events associated with corticosteroid use [186].<br>Corticosteroid has potential for treating ARDS because of its potent anti-inflammatory, and pulmonary vasodilator actions. However, results of its use in both clinical and experimental studies in acute lung injury, ARDS or sepsis are often contradictory [187].<br>A systematic review of the clinical evidence from retrospective studies found contradictory results and could not infer a definitive protective benefit with corticosteroids for COVID-19 [188].<br>Clinical evidence does not support the use of corticosteroid for COVID19 lung injury [189].

Dexamethasone (DEX)	**Positive:** Administering DEX demonstrated a significant decrease in respiratory support need and improved pulmonary function for infants with bronchopulmonary dysplasia (BPD). Pulmonary inflammation was also suppressed [190].<br>A multicenter, randomized controlled trial of 17 intensive care units found early treatment of DEX could reduce duration of mechanical ventilation and overall mortality in patients with established moderate-to-severe ARDS [191].<br>In a controlled, open-label trial of COVID19, DEX lowered mortality among those who were receiving either mechanical ventilation or oxygen alone but not among patients receiving no respiratory support [192].<br>A systematic review of the clinical evidence indicated that the RECOVERY trial found significantly better outcome with DEX in severe cases of COVID-19 but need further replication of the outcomes to be conclusive [188].<br>In a multicenter, randomized, open-label, clinical trial<br>conducted of intensive care units in Brazil found the number of days the patients remain alive and free from mechanical ventilation during the first 28 days was significantly higher among patients treated with DEX plus standard care as compared with standard care alone [193].
Disulfiram (DS)	**Positive:** Suppresses lung colonization and induces cell death in Triple Negative Breast Cancer (TNBC), kills TNBC cells in a copper (Cu)-dependent manner [194].<br>Addition of DS to chemotherapy prolong survival of non-small cell lung cancer patients [195].<br>DS has been found to be an inhibitor of SARS and MERS [196].
Dociparstat Sodium (DSTAT, aka ODSH)	**Positive:** Neutrophil elastase (NE) is an inflammatory mediator in cystic fibrosis (CF) and DSTAT inhibits NE activity to block NE-induced airway inflammation. DSTAT inhibited NE-triggered high mobility group box 1 (HMGB1) secretion from macrophages and inhibited neutrophil elastase–induced airway inflammation in cystic fibrosis in animal and cell studies [197].<br>HMGB1 is a proinflammatory cytokine and plays an important role in pseudomonas aeruginosa lung infections. DSTAT reduced the binding of HMGB1 to its receptors TLR2 and TLR4. DSTAT reduced PA-induced lung injury and increased bacterial clearance [198].
Emtricitabine	**Positive:** Emtricitabine seems to have greater virological efficacy and lower probability of an adverse event than stavudine [199].<br>Emtricitabine-tenofovir treated ferrets showed reduced overall clinical scores and shorter duration of clinical symptoms than PBS-treated control group, furthermore, the virus titers in nasal washes of the emtricitabine-tenofovir group was lower than in the PBS-treated control group [200].<br>A cohort study of HIV-positive patients receiving TDF/emtricitabine was found to have a lower risk for COVID-19 and related hospitalization than other HIV therapies, including TAF/emtricitabine [139].<br><br>**Negative:** In a prospective cohort study, adults with HIV and pulmonary tuberculosis experienced declines in lung function on emtricitabine, tenofovir, and efavirenz therapy [201].
Entecavir (EVT)	**Positive:** EVT inhibited cytokine storm syndrome and attenuated acute lung injury in LPS-induced mice [202].<br><br>**Negative:** A patient with HBV-associated vasculitis presenting with multiple pulmonary nodules was treated with EVT. EVT worsened the patient’s lung nodules, but resolved the HBV when she was treated with short-term prednisone plus EVT [203].<br>EVT’s adverse effects include upper respiratory tract infection, fatigue, and lactic acidosis [204].

Epoprostenol (EPO, synthetic equivalent of prostacyclin)	**Positive:** Used to treat pulmonary arterial hypertension (PAH). Inhaled EPO (prostacyclin) is an effective pulmonary vasodilator and associated with reduced mean pulmonary artery pressures without altering the mean arterial pressure [205].<br>In a clinical trial, the drug was found to improve symptoms and to reduce mortality in patients with idiopathic PAH. It also improved hemodynamic in some patients with idiopathic PAH [206].<br>In a retrospective cohort study, short-term use of EPO for moderate-to-severe ARDS improved oxygenation [207].<br>Intravenous EPO was used in a small study of patients to treat pulmonary hypertension associated with systemic sclerosis. Overall, EPO was well-tolerated [208].<br>A study of three severe cases of COVID-19 patients with acute onset of bilateral lung infiltrates, and hypoxaemia, treated with 5 days of intravenous infusion of iloprost, a prostacyclin receptor agonist that promotes vasodilation, achieved sustained clinical improvement in the digital ischaemia [209].<br>A retrospective observational study of patients mechanically ventilated for COVID-19 found that 50% of the patients receiving inhaled EPO for hypoxemia showed clinically significant improvement [210].<br><br>**Neutral:** A retrospective single-center study found that inhaled EPO and inhaled NO in patients with refractory hypoxemia secondary to COVID-19 did not produce significant increases in oxygenation. However, a group of patients were responsive and had significant improvement with inhaled EPO and inhaled NO [211].
Famotidine	**Positive:** In a physician-sponsored cohort study, a combination of cetirizine and famotidine reduced pulmonary symptom progression in COVID-19 patients [212].<br>A retrospective study found patients hospitalized with COVID-19 and treated with famotidine was associated with a reduced risk of clinical deterioration leading to intubation or death [213].<br><br>**Negative:** A population-based cohort study found an increase in risk of pneumonia when COPD patients had used glucocorticoids concurrent with histamine receptor 2 antagonists (HR2As, e.g. famotidine) [214].
Favipiravir	**Positive:** Anti-viral approved in Japan to treat influenza. Effective against influenza viruses, including RNA viruses [215].<br>Also effective against Hantavirus Pulmonary Syndrome [216].<br>Its use on COVID19 patients in an open-label comparative controlled study and preliminary results indicate faster viral clearance [217].<br>A prospective, randomized, controlled, open-label multicenter trial comparing favipiravir and arbidol for COVID-19 found latency to fever reduction and cough relief in the favipiravir group was significantly shorter than that in the arbidol group, and adverse events associated with favipiravir included increased levels of serum uric acid. A control group of standard care was not included [218].
Hydroxy Chloroquine (HCQ)	**Positive:** This antimalarial drug is being used to treat some patients with systemic lupus erythematosus (SLE) [219]. Treatment of HCQ for SLE has a higher probability of long-term continuation [220].<br>CQ and HCQ accumulate in lysosomes and raise endosome pH which interferes with viral entry and exit. May reduce glycosylation of ACE-2 receptors and prevent SARS-CoV-2 from binding to host cells. It is used with chemotherapy drugs to treat non-small cell lung cancer (NSCLC) cells reversed the chemotherapy drug sequestration in lysosomes to chemo-sensitize the cancer cells. HCQ also enhanced $CD8^+$ T cell immune response [221].<br>Improved airflow and lowered IgE levels in asthma [219].

	Found effective in treating pulmonary sarcoidosis, which is an inflammatory disease that affects multiple organs in the body, including the lungs [222].<br>HCQ successfully inhibited growth of the SARS-CoV-2 virus in vitro [223].<br><br>**Neutral:** Clinically HCQ appears to have no effect in either lowering or an increasing risk of intubation or death in hospitalized COVID-19 patients in an observational study [224].<br>A randomized trial of HCQ did not benefit postexposure prophylaxis for Covid-19 [225].<br><br>**Negative:** In contrast, treatment of patients with RA has a low probability of long-term continuation due to inadequate control of the disease manifestations [220].
IC14	**Positive:** CD14 plays an important role in lung inflammation and infection by recognizing and binding a variety of (myco)bacterial and viral constituents. IC14 reduced LPS responsiveness in humans [226].<br>Could protect the lungs by blocking the inflammatory signals that CD14 activates. The antibody showed promise in sepsis [227], which is often associated with excessive inflammation leading to shock and multi-organ failure [228].
Idelalisib	**Negative:** Idelalisib impairs T-cell-mediated immunity in CLL and this impairment induced by idelalisib could favor infections and/or viral reactivations [54].<br>A study found idelalisib induced (lung inflammation) pneumonitis in CLL patients [229].
IFN-α	**Positive:** A study of 40 patients treated with a combination of IFN-α, RTV and LPV accelerated viral clearance of COVID-19 patients [230].<br>More patients were discharged from hospital without clinical and radiological symptoms in the IFN-treated compared with the non-IFN treated group. The case fatality rate was reduced in the patients that received IFN-α2b [231].<br>A study of COVID-19 patients in one hospital treated with IFN-α2b significantly reduced the duration of detectable virus in the upper respiratory tract [57].<br><br>**Negative:** Type I Interferon receptor signaling amplifies acute lung proinflammatory cytokine response during respiratory syncytial virus infection [232].<br>A HCV patient developed interstitial granulomatous pneumonitis after therapy with IFN and ribavirin [233].<br>In support, there are other cases of IFN-associated pulmonary pathology in patients with malignancy [234-238].
Ivermectin	**Positive:** An anti-parasitic drug. Exerts antitumor effects in various types of cancer (including lung cancer) by inhibiting the WNT-TCF pathway [239].<br>The drug was found to inhibit the replication of SARS-CoV-2 in vitro [240].
Leronlimab (PRO 140)	**Positive:** Being investigated for HIV, TNBC, and COVID19 [241-244].<br>Compassionate use of leronlimab of COVID-19 patients was well tolerated but not all the treated patients appear to have strong clinical response (e.g. inflammatory markers), however adverse events were not noted [245].<br>Ten critical COVID-19 patients with elevated IL-6 and CCL5 levels, and decreased CD8+ T cell levels, treated with leronlimab reduced IL-6 level and significantly reduced SARS-CoV-2 plasma viremia [246].<br><br>**Negative:** CCR5 is required for memory T cell recruitment and expression of antiviral molecules

	(which is needed to limit early virus replication) to lung airways during virus challenge [247].<br>Inhibiting CCR5 in mice has been shown to protect against pulmonary arterial hypertension [248].<br>CCR5 KO mice showed an excessive inflammatory response and increased mortality to influenza A [249].
Lopinavir/ Ritonavir (LPV/RTV)	**Positive:** Its use to treat MERS infection in nonhuman primates was found to reduce viral load and lung damage [250].<br>In a study of publications on cases of severe COVID-19 treated with LPV/RTV, time to clinical improvement was not significantly different for LPV/RTV in comparison to standard of care, however there appeared to be fewer cases of acute respiratory distress syndrome with LPV/RTV compared to standard of care [251].<br><br>**Neutral:** A randomized controlled, open-label trial of severe COVID-19 hospitalized patients treated with LPV/RTV did not provide a substantial benefit beyond standard care [252].<br>In an exploratory randomized controlled clinical trial treating mild to moderate COVID-19 cases with LPV/RTV did not provide significant advantage over supportive care [253].
Methyl Prednisolone (MP)	**Positive:** Ameliorated acute lung injury (ALI) by promoting macrophage M2 polarization [254], which is involved in modulating the inflammatory response [255].<br>A single-blind, randomized, controlled, clinical trial of 68 severe hospitalized COVID-19 patients in Iran using intravenous MP was found to be effective at reducing recovery time for hospitalized patients as compared to standard care, although two patients were noted to have adverse effects (infection and oedema) [256].<br><br>**Neutral:** A meta-analysis suggested that prolonged MP treatment could help resolve ARDS [257], although it remains controversial [258].<br>A parallel, double-blind, placebo-controlled, randomized, Phase IIb clinical trial in Brazil using MP for COVID-19 found no difference in virus clearance and did not reduce mortality [259].<br><br>**Negative:** Systemic treatment with MP increases matrix metalloproteinases activity in the lung and caused emphysema in rats [260].
Nafamostat (NM)	**Positive:** May improve lung function. NM prevented pulmonary vascular injury and coagulation abnormalities in lipopolysaccharide (LPS)-induced pulmonary vascular injury in rats [261].<br>NM suppressed radiographic contrast media induced pulmonary dysfunction in rats [262].<br>NM inhibits lung metastasis of TNBC cells in mice xenograft model [263]. This was achieved by inhibiting TGFβ-stimulated Smad2 phosphorylation, metastasis-related gene expression (matrix metalloproteases (MMPs) and urokinase plasminogen activator), and downregulated extracellular signal-regulated kinase (ERK) activity.<br>NM inhibits SARS-CoV-2 infection of lung-derived human cell line Calu-3. NM inhibited SARS-CoV-2 S-mediated entry into host cells with higher efficiency than camostat mesylate *in vitro* [264].<br>A study of critically ill COVID19 patients on mechanical ventilation treated with NM and favipiravir showed low mortality rate [265].
Niclosamide	**Positive:** Niclosamide was able to inhibit the replication of SARS-CoV and reduce the viral yields in the culture supernatants from SARS-CoV infected Vero E6 cells [266].<br>It inhibited LPS-induced release of IL-8 in Calu3 airway epithelial cells. It also reduced mucus production and secretion, and bronchoconstriction in mice likely by blocking TMEM16A/F [267].<br>It can reverse radioresistance of human lung cancer cells [268] and sensitized non-small cell lung cancer cells to radiotherapy [269].

	Nasal and pulmonary delivery of niclosamide through particles of human lysozyme as a carrier molecule was found to reduce viral load in the lungs of hACE2 transgenic mice infected intranasally with SARS-CoV-2 [270].
Nitazoxanide (NTZ)	**Positive:** NTZ is a potent TMEM16A antagonist that blocked airway smooth muscle depolarization and contraction and promoted bronchodilation of mouse airways [271].<br>NTZ reduced the duration of clinical symptoms with acute uncomplicated influenza and reduced influenza viral shedding [272].<br>It was found to amplify the host innate immune response to viruses and inhibited Ebola virus replication [74].<br>NTZ was found to inhibit SARS-CoV-2 replication and infection in Vero E6 cells [273, 274].<br>In clinical settings of COVID-19 patients, NTZ showed a positive outcome with some patients weaned from mechanical ventilation [275].
Nitric Oxide (NO)	**Positive:** The impact of inhalation of NO by patients with severe adult respiratory distress syndrome (ARDS) increases oxygenation [276] and reduces the pulmonary-artery pressure [277].<br>NO was found to inhibit the replication cycle of SARS CoV in vitro [278].<br>Inhalation of NO showed improved arterial oxygenation, reduced inspired oxygen therapy and airway pressure support in patients with SARS [279].<br>In a retrospective cohort study, it was found that both inhaled EPO and inhaled NO were comparable in terms of oxygenation and ventilation parameters for moderate to severe ARDS [207].<br>The burst of NO increases O_2 saturation and has been suggested that it could be protective against COVID19 [280].<br><br>**Neutral:** In a single-center retrospective case–control study of patients with ARDS treated with iNO, found that half the patients with refractory hypoxaemia (inadequate arterial oxygenation) secondary to COVID-19 ARDS did not show an increase in PaO2/FiO2 ratio in response to iNO and the response was much lower compared with non-COVID-19 ARDS patients [281].
Remdesivir (RDV)	**Positive:** In nonhuman primates with SARS-CoV-2 infection, RDV administered early had lower viral load in the lungs and less lung damage [282].<br>In support, a systematic review found RDV inhibits SARS-CoV-2 replication, reduces viral load, and has protective effects in SARS-CoV-2 infected animals [283].<br>This drug is a nucleotide polymerase inhibitor, known to reduce viral replication [284] and also found to potently block SARS-CoV-2 virus infection in vitro [274].<br>RDV inhibits SARS-CoV-2 replication in human lung cells and primary human airway epithelial cells, and reduces lung viral load and improved pulmonary function in mice infected with chimeric virus [285].<br>The efficacy and safety of RDV were analyzed through a literature search of observational and interventional studies of COVID-19 patients. Analyzing the mortality rates, clinical improvement, and adverse events revealed that the 5-day RDV group had a lower severe adverse event rate as compared to 10-day and placebo groups and patients without invasive mechanical ventilation were benefited more [286].<br>In a compassionate use of RDV for COVID-19 it improved oxygen support but the study lacked a placebo control [287].<br>In a double-blind, randomized, placebo-controlled trial of intravenous RDV in hospitalized adults with Covid-19 had shortening time to recovery and evidence of lower respiratory tract infection [288].<br><br>**Neutral:** In a randomized, double-blind, placebo-controlled, multicenter clinical trial RDV was not

	associated with statistically significant reduction in time to clinical improvement, viral clearance, or mortality in adults with severe COVID-19, but requires confirmation in larger study [289].
Ruxolitinib (RUX)	**Positive:** RUX improved pulmonary hypertension in patients with myelofibrosis. It increased plasma levels of NO, and reduced key cytokines (tumor necrosis factor alpha, interleukin-4 (IL-4), IL-1β, IL-2, IL-6 and IL-8) and increased interferon-gamma (IFN-γ), IL-1α, IL-17A, and IL-10 [290].<br>Preliminary studies of COVID-19 patients with inflammatory syndrome treated with RUX showed improvement [82, 291].<br><br>**Neutral:** A prospective, multicenter, controlled, randomized, single-blind study of patients with severe COVID19 treated with RUX was not associated with significantly accelerated clinical improvement and RUX recipients had similar median time of virus clearance compared with patients in the control group [292].<br><br>**Negative:** Given that RUX targets IFNα and IFNλ induced by JAK/STAT, a recent study in lung cell line, Calu-3 (IFN-competent cells), found that RUX increased SARS-CoV-2 replication, likely by inhibiting JAK/STAT signaling and IFN types I and III inhibited its replication [293].<br>A case study found RUX exacerbated pulmonary arterial hypertension in a patient [294], while another study found ARDS developed secondary to RUX use [295].
Sarilumab	**Positive:** Inhibits IL-6 pathway. IL-6 signaling contributes to pulmonary arterial hypertension (PAH) and inhibiting IL-6 receptor signaling protected against chronic hypoxia-induced PAH [296].<br><br>**Neutral:** Studies are being conducted to determine the effectiveness of sarilumab in patients with severe cases of COVID-19. Thus far, an open-label cohort study did not find significant difference between sarilumab and standard care, although the drug was associated with faster recovery for a subset of patients with minor lung consolidation [297].<br><br>**Negative:** There have been cases of alveolar hemorrhage associated with sarilumab [298, 299].
Sirolimus (Rapamycin)	**Positive:** Rapamycin may be beneficial for treating various diseases [300].<br>Improved several parameters of lung function in transgenic mice model of noninflammatory lung disease [301].<br>Sirolimus stabilized lung function, reduced serum VEGF-D levels in patients with lymphangioleiomyomatosis (LAM) [302].<br>LAM, a rare cystic lung disease found predominantly in women, is linked to MTOR signaling and tuberous sclerosis complex (TSC) [303].<br>Has been suggested as a possible therapy for COPD [304], based on studies in a transgenic mouse model [301] and in patients with mild to severe COPD in which rapamycin restored corticosteroid sensitivity [305].<br>In patients with severe H1N1 pneumonia, corticosteroids plus sirolimus was associated with improvement in multiple organ dysfunction, virus clearance, and shortened need for ventilator [306].<br><br>**Negative:** In a meta-analysis of rapamycin and its analogues for treating TSC, which can occur in multiple organs including the lungs, rapamycin increased the risk of stomatitis but did not improve or increase risk of upper respiratory tract infections [307].<br>Sirolimus is associated with venous thromboembolism in lung transplant patients [308].<br>Mixed results when treating bleomycin-induced lung fibrosis in rats and mice [304].<br>A clinical trial of everolimus (an MTOR inhibitor) found it worsened outcomes in patients with IPF

	[309].
Sofosbuvir	**Positive:** Sofosbuvir was found to inhibit SARS-CoV-2 replication in human hepatoma-derived (Huh-2) and Type II pneumocyte-derived (Calu-3) cells [310].<br>SARS-CoV-2 has an exonuclease-based proofreader to maintain viral genome integrity and sofosbuvir displayed a higher level of resistance to this proofreading activity than remdesivir [311].<br>In an open-label, multicenter, randomized controlled clinical trial in moderate and severe COVID-19 patients found adding sofosbuvir and daclatasvir to standard care significantly reduced the duration of hospital stay compared with standard care alone [312].<br><br>**Negative:** Case study suggests a possible association with the worsening of pulmonary arterial hypertension (PAH) in HCV patients [313].<br>Sofosbuvir plus simeprevir could contribute to pulmonary toxicity in HCV infected patients [314].
Statins	**Positive:** A retrospective study of COVID19 patients found that statins use was associated with a lower prevalence of ARDS and a lower risk of mortality [315].<br><br>**Neutral:** A retrospective multi-center cohort study found that the use of statins was significantly associated with the absence of COVID-19 symptoms in some of the patients. However, the effects of statin intake on serious clinical outcomes (long-stay hospitalization or death) were not statistically significant. In addition, there was no statistically significant association between angiotensin-converting enzyme inhibitors (ACEi), angiotensin II receptor blockers (ARB) and asymptomatic status [316].<br>A large cohort study did not find an association between statin use and the incidence of ILD [317].<br><br>**Negative:** Lowers cholesterol levels, however, can cause serious lung injuries. In a case study rosuvastatin caused diffuse ground-glass opacities, several subpleural ground-glass opacity nodules, and a foamy alveolar macrophage appearance [318].<br>Several cases of statin-induced interstitial lung disease (ILD), which could lead to irreversible fibrosis, have been reported [319].
Stavudine	**Negative:** The time to develop severe drug toxicities were shorter and virological efficacy was lower in the regimens containing stavudine as compared to emtricitabine when used with didanosine and efavirenz [199].
Tenofovir	**Positive:** TAF showed anti-fibrotic effects and inhibited TGF-β in a bleomycin-induced murine model of pulmonary fibrosis [320].<br>Tenofovir has been suggested to potentially inhibit SARS-CoV-2 polymerase [321].<br><br>**Neutral:** An observational prospective study of HIV-infected individuals on tenofovir did not affect their risk of severe SARS-CoV-2 infection [322].
Tetrandrine (TET)	**Positive:** Has been used to treat asthma and pulmonary hypertension. It has anti-allergic, antioxidant, and anti-fibrogenic effects on lungs [323].<br>It also has anticancer properties [324].<br>TET reduces A549 lung cancer cell viability and induces apoptosis possibly through the VEGF/HIF-1α/ICAM-1 signaling pathway. Significantly upregulated the expression of intercellular adhesion molecule-1 (ICAM-1) but downregulated expression of vascular endothelial growth factor (VEGF) and HIF-1α [325].

	**Negative:** Human lung cell lines treated with TET reduced cell viability by up to 60%. TET caused acute lung injury in mouse studies with massive bronchiolar and alveolar hemorrhage and edema [326].
Thalidomide (THD)	**Positive:** A case-report of a patient with severe COVID-19 pneumonia treated with THD in combination with low-dose methylprednisolone suggests that THD had a positive outcome, likely contributing to reducing elevated cytokine levels [327].<br>THD had anti-inflammatory effect on H1N1-induced acute lung injury in BALB/C mice which contribute to attenuating H1N1-induced pulmonary injury [328].<br>A single-center study found THD improved cough and respiratory quality of life in patients with IPF [329].<br><br>**Mixed:** Although THD was not found to significantly improve pulmonary function in patients with corticosteroid-dependent sarcoidosis [330], it has been suggested that the drug could be helpful for pulmonary sarcoidosis in patients resistant or with contraindications to corticosteroids [331].<br><br>**Negative:** THD has been found to cause acute pulmonary toxicity in a case study of multiple myeloma patient [332].<br>THD effectiveness on several pulmonary diseases and lung injuries were performed in animals, nonetheless there are concerns with the use of THD given its undesirable side effects and the associated toxicities [333].<br>Nonetheless, there is concern in the use of THD with corticosteroids [333].
Tocilizumab (TCZ)	**Positive:** A case report of COVID-19 patient found TCZ altered the inflammatory response by blocking cellular receptors for IL-6 and resulted in a favorable outcome [334].<br>In a retrospective study, lung opacities of severe COVID-19 patients were absorbed in over 90% of patients treated with TCZ [335].<br>In an observational cohort study, TCZ improved lung function in COVID-19 patients with CRS [336].<br>In a retrospective, observational cohort study of adults with severe COVID-19 pneumonia found TCZ treatment was associated with reduced risk of invasive mechanical ventilation or death [337].<br>Similarly, another prospective study of COVID-19 pneumonia associated TCZ with significant clinical improvement [338].<br>In a Rapid Review, a comparison of serum IL-6 concentration in patients with severe or critical COVID-19 with other conditions, found the mean IL-6 levels were ~100 times higher in patients with CRS and 12 times higher in patients with ARS unrelated to COVID-19 [339].<br>Analysis of a multicenter cohort study of adult COVID-19 patients in intensive care units at 68 hospitals across the US found, after multivariable adjustment, TCZ treatment was associated with a lower risk of invasive mechanical ventilation or death as compared with those not treated with TCZ [340] .<br>An observational study found TCZ administered early during the disease course to patients with severe COVID-19 is associated with decreased intubation and reduced mortality; notably patients requiring ≤45% fraction of inspired oxygen (FiO2) was associated with better clinical outcomes when treated with TCZ than patients requiring >45% FiO2 [341].<br>An observational study of 239 COVID-19 patients with CRS found TCZ-treated patients with severe COVID-19 disease had higher admission levels of high-sensitivity CRP, received TCZ sooner, and their survival was similar to patients with nonsevere COVID-19 disease [124].<br><br>**Neutral:** In a randomized, double-blind, placebo-controlled trial of hospitalized COVID-19 patients TCZ was not effective in preventing intubation or death in moderately ill patients [342]. See also

	**Table 4:** COVID-19 Clinical Trial Results. Several randomized, double-blind, placebo-controlled trials did not find TCZ improved clinical outcomes or mortality. In contrast, an international trial of critically ill COVID-19 patients found TCZ improved survival.<br><br>**Negative:** ARDS is connected with CRS and IL-6. TCZ is an IL-6 inhibitor and could halt the inflammatory response by blocking cellular receptors for IL-6. However, TCZ was found to induce pulmonary fibrosis in a RA patient [343].
Umifenovir (Arbidol)	**Positive:** Has an antioxidant effect [344] and efficiently inhibits H1N1 strain and diminishes both viral replication and acute inflammation by modulating inflammatory cytokine expressions [345].<br>Arbidol (umifenovir) reduced the viral load (RNA level) in the heart and lungs of mice with the Coxsackievirus B5 infection [346].<br>Arbidol has been suggested as a possible candidate for further investigation as a broad-spectrum respiratory antiviral [347].<br>This drug was able to inhibit SARS-CoV-2 virus *in vitro* [160].<br>In a study of health care professionals, the drug was associated with a lower incidence of SARS-CoV-2 infection but not hospitalization rate [348].<br><br>**Neutral:** In an exploratory randomized controlled clinical trial arbidol monotherapy was used to treat mild to moderate COVID-19 cases and did not provide significant advantage over supportive care. Arbidol did not significantly improve recovery time or alleviate symptoms, and no difference was observed in the rates of antipyresis, cough alleviation, or improvement of chest computed tomography [253].<br>In a single-center retrospective study, patients with SARS-CoV-2 infection (not in ICU) treated with umifenovir did not appear to improve CT score, recovery time, or length of stay in the hospital [349].
Valganciclovir/ Ganciclovir	**Positive:** A lower incidence of CMV infection/disease and acute rejections in lung transplant patients treated with valganciclovir as compared to ganciclovir [350, 351].
Zidovudine (AZT)	**Negative:** AZT affects the structural development of human fetal lung tissue in vitro [352].<br>AZT was found to exert cytotoxic effects on human myogenic cells [353] and contribute to myopathy associated with this drug [354].

Supplementary Table 4: Drug Effects on the Heart

Drugs	Heart
Abacavir	**Positive:** In healthy, virologically suppressed HIV-infected patients, abacavir did not cause inflammation, endothelial dysfunction, insulin resistance as compared with TAF/emtricitabine [355].<br><br>**Negative:** An increased risk of myocardial infarction has been seen with abacavir [356, 357]. Abacavir/lamivudine increased total and LDL cholesterol [355].<br>Abacavir was independently associated with impaired endothelial function, a marker for CVD risk [358].
Anakinra	**Positive:** Improved coronary flow, left ventricle function, endothelial function and reduced inflammation in RA patients [359].<br>Anakinra reduced systolic BP in mice with one kidney, deoxycorticosterone acetate implantation, and salt induced hypertension [360].<br>IL-1 plays a central role in the pathogenesis of cardiac inflammation (myocarditis) and anakinra is effective in treating this condition [361-363].<br>A COVID-19 patient with myocarditis developed acute respiratory failure and cardiogenic shock due to cytokine storm-like syndrome and treated with anakinra and DEX had rapid clinical improvement, reduced serum inflammatory markers and marked recovery in cardiac magnetic resonance-based markers of inflammation and contractile dysfunction [364].
APN01 (rhACE2)	**Positive:** Attenuates negative effects of ANG II and improves remodeling of post-myocardial infarction [365].<br>rhACE2 prevented development of hypertension and heart fibrosis in renin transgenic mice and attenuated ANG II-induced cardiac hypertrophy and fibrosis [366]. S<br>Studies of severe COVID-19 patients treated with human rhACE2 markedly reduced angiotensin II [367] and increased ACE2 levels [9].
Ascorbic Acid (AA)	**Positive:** AA significantly improves endothelial dysfunction of epicardial coronary arteries of patients with hypertension [368].<br>AA in vascular smooth cells may prevent apoptosis, which can help stabilize plaque if atherosclerosis develops [369].<br>A review of epidemiologic studies suggests vitamin C deficiency is associated with a higher risk of mortality from cardiovascular disease but vitamin C's improvements on endothelial function and lipid profiles are slight, and are more likely to improve for groups with low plasma vitamin C levels [370].<br>In a mice study, vitamin C was shown to decrease TNFα expression and decrease pathological damage to the myocardium. It also shows some therapeutic and protective effects against the progression of experimental autoimmune myocarditis [371].
Azithromycin (AZM)	**Neutral:** In a clinical trial no significant increase in arrhythmia, cardiac arrest, acute kidney failure, or QT interval prolongation between the control and AZM group in severe COVID-19 patients in Brazil [372].<br>A retrospective pharmacoepidemiological study indicate a combination of CQ and AZM did not show pronounced increases in arrhythmias, although the sample size was small [373].<br><br>**Negative:** Five days of AZM therapy was shown to have an increased risk of cardiovascular deaths, more so in patients with higher risk scores for cardiovascular disease, and increased risk of sudden

	cardiac death [374]. It has been associated with QT-interval prolongation and torsade de pointes [375, 376].<br>In a case study, fulminant myocarditis was associated with AZM exposure [377].
Baloxavir	**Neutral:** No adverse cardiovascular effects observed in preclinical trials for influenza [378].
Baricitinib	**Mixed:** Meta-analysis of randomized trials of patients with RA treated with baricitinib was associated with a dose response increase in both HDL and LDL cholesterol. This could potentially have adverse cardiovascular effects, however in this study baricitinib did not statistically alter cardiovascular risk [379]
Brincidofovir/ Cidofovir	**Limited Information:** Also known as CXM001 and used to prevent CMV in hematopoietic cell transplantation [380].
Camostat Mesylate (CM)	**Limited Information:** CM's effects on the cardiac system has not been studied. However as a serine protease inhibitor it could affect processes involved in cardiac function (potential to reduce cardiac inflammation and fibrosis) [381].
Chloroquine (CQ)	**Neutral:** A retrospective cohort study of COVID-19 patients treated with CQ/HCQ and AZM did not find pronounced increases in arrhythmias [373].<br><br>**Negative:** CQ is associated with cardiotoxicity and cardiomyopathy [382].<br>HCQ/CQ known to produce mild cellular and cardiac toxicities. Other cardiac adverse effects include hypertrophy, HF, valvular dysfunction, and pulmonary arterial hypertension. These adverse outcomes improved in some patients upon HCQ/CQ withdrawal, while the effects were irreversible in some patients or even led to mortality in others [383].<br>CQ induced QTc prolongation in COVID-19 patients [384, 385].
Colchicine	**Positive:** Colchicine is an anti-inflammatory used to treat gout and has been shown to reduce prevalence of myocardial infarction and cardiovascular events [375, 386].<br>In a randomized, double-blind trial of patients recruited within 30 days after myocardial infarction colchicine treatment led to a significantly lower risk of ischemic cardiovascular events than placebo [387].<br>A randomized, controlled, double-blind trial of patients with chronic coronary disease the risk of cardiovascular events was significantly in patients taking colchicine [388].<br>A study of 105 patients in Greece noted significant clinical benefit of colchicine in COVID-19 hospitalized patients, however there were no significant differences in cardiac troponin or CRP levels in the treated vs. control group [26].<br>In contrast, a single-center randomized, double-blinded, placebo controlled clinical trial of colchicine for moderate to severe COVID-19 found the colchicine group showed significant reduction of serum CRP [389].<br>In a prospective, open-label, randomized and double blind clinical trial, 100 patients hospitalized with COVID-19 colchicine was found to reduce systemic symptoms by inhibiting inflammatory biomarkers [390].<br><br>**Neutral:** In an open-label, randomized clinical trial for COVID-19 there was no significant differences in high-sensitivity cardiac troponin or CRP levels in the treated vs. control group of standard care patients [26].<br><br>**Negative**: In a mouse model of coxsackievirus B3 (CVB3)-induced myocarditis, colchicine had negative effects. It increased the viral load in the heart and the pancreas as well as destroying the

	exocrine pancreas [391].<br>In case reports, patients with chronic renal insufficiency colchicine was associated with cardiac insufficiency and death [392].<br>Similarly, colchicine was found to contribute to cardiotoxicity in a heart transplant patient with chronic renal failure [393].
Corticosteroid	**Positive:** Corticosteroids can increase atrioventricular conduction and is used to treat symptoms caused by pericarditis or myocarditis [394].<br>A nested case-control analysis suggests that low doses of inhaled corticosteroids may be associated with reduced risk of acute myocardial infarction in COPD patients [395].<br>In a hospital in France, adding corticosteroids treatment of children with multi-inflammatory syndrome in children with COVID-19 aid in the recovery of cardiac function [396].<br>A study of 13 Italian cardiology units found treating COVID-19 patients with corticosteroids and heparin were associated with lower mortality, although patients with HF were less likely to be treated with corticosteroid [397].<br><br>**Mixed:** The use of corticosteroid as an immunomodulatory therapy for influenza is inconclusive and controversial [398].<br><br>**Negative:** Two major adverse effects of glucocorticoids are dyslipidemia and hypertension [399].<br>In patients without diabetes, corticosteroid treatment increases the risk of glucose intolerance. In patients with diabetes, it is associated with decreasing glycemic control [400].<br>High dose corticosteroid therapy increases risk of developing atrial fibrillation [401].
Dexamethasone (DEX)	**Positive:** A case report of a child with multi-viral infection including SARS-CoV-2 with respiratory distress, myocarditis, and coronary aneurysms was treated with intravenous immunoglobulins, aspirin, diuretics, DEX, and HCQ recovered with normalization of left ventricular function [402].<br>A COVID-19 patients with myocarditis was treated with anakinra and DEX showed clinical improvement, reducing serum inflammatory markers and reduced cardiac magnetic resonance-based markers of inflammation and contractile dysfunction [364].<br><br>**Negative:** Myocardial hypertrophy is associated with Dex therapy in infants with bronchopulmonary dysplasia [403].<br>Chronic DEX treatment induces insulin resistance, hyperinsulinemia, and hyperglycemia [404, 405].<br>Fetal sheep treated with DEX saw a drop in the partial pressure of oxygen [406] and an increase in their arterial blood pressure [407].
Disulfiram (DS)	**Negative:** DS could contribute to myocardial infarction, arrhythmia, chronic heart failure (HF), or death [408]. DS was observed to have significant cardiovascular effects such as chest pain, palpitation, tachycardia, and hypotension in patient undergoing alchohol detoxification [409].
Dociparstat Sodium (DSTAT, aka ODSH)	**Positive:** Formerly known as CX-01, which is an inhibitor of CXCL12/CXCR4. A promising glycosaminoglycan derivative of heparin, this drug anticoagulant activity decreases myocardial reperfusion injury in an animal model [410].<br>ODSH reduced infarct size in ischemic-reperfused porcine myocardium [410].<br><br>**Mixed:** The role of CXCL12/CXCR4 on the cardiovascular system (and thereby inhibiting it) could be beneficial (e.g. increase cardiac function) or harmful (e.g. increase TNFα expression and cardiomyocyte apoptosis or reduce infarction size when deficient in CXCR4) depending on the condition [411-413].

Emtricitabine	**Positive:** Used to treat HIV and hepatitis B infection [414]. Emtricitabine/TAF elicited decreases in serum total, HDL and LDL cholesterol [415].<br><br>**Negative:** An episode of syncope was reported while treating an HIV patient with tenofovir, emtricitabine and nevirapine [416]. See also TAF.
Entecavir (EVT)	**Limited Information:** Used to treat chronic hepatitis B. Myopathy cases have been reported with NRTIs including EVT [417], however entecavir's effect on the heart is not known.
Epoprostenol (EPO, synthetic equivalent of prostacyclin)	**Positive:** EPO is a vasodilator and inhibits platelet aggregation. Studies support its use in pulmonary arterial hypertension. PG12 improves cardiac output [418].<br><br>**Negative:** However, it can exacerbate underlying myocardial dysfunction and is contraindicated in HF [419].<br>Similarly, infusion of EPO in patients with severe left ventricular dysfunction was associated with increased mortality rates, without improvement in quality of life [420].<br>It can also increase plasma level of atrial natriuretic peptide, which impacts blood pressure [421].
Famotidine	**Negative:** Based on hemodynamic parameters famotidine treatment exerted a negative effect on cardiac performance and led to reduced stroke volume and cardiac output in healthy patients and patients with CHF [422].
Favipiravir	**Negative:** Treatment increased QTc interval prolongation in an Ebola infected patient [423].<br>A risk assessment study of several drugs being used for COVID-19 found that there is some risk of long QT with favipiravir [424].
Hydroxy Chloroquine (HCQ)	**Positive:** HCQ is independently associated with a decreased risk of cardiovascular morbidities in RA patients [425].<br><br>**Negative:** HCQ is known to have a safer profile than CQ. It nonetheless has led to QT prolongation, arrhythmia [425] and cardiomyopathy [426].<br>QTc interval prolongation risk increased with HCQ and torsades de pointes was also observed in certain COVID-19 patients [427].<br>In COVID-19 patients combinatory treatment of HCQ and AZM for SARS-CoV-2 significantly prolong the QTc interval [428], but did not appear to promote torsade de pointes or arrhythmia-related death [429, 430].<br>A systematic review of the arrhythmogenic effect of short courses of CQ or HCQ found significant QT-interval prolongation in patients treated with these drugs [431].
IC14	**Limited Information:** IC14's effect on the heart has not been thoroughly investigated. However, soluble CD14 plays a role in inflammation, coronary heart disease, and chronic HF [432, 433], and IC14 suppressed LPS-stimulated TNF production in patients which could be beneficial in chronic HF patients with systemic immune activation. In patients with chronic HF, IC14 suppressed TNFα expression. The suppression of TNFα was found in both chronic HF and healthy patients [434].
Idelalisib	**Limited Information:** In a study of the efficacy and safety of idelalisib in patients with relapsed, rituximab- and alkylating agent-refractory follicular lymphoma, there were 2 patients with adverse events that were considered possibly (acute cardiac arrest) or probably (drug-induced pneumonitis) related to idelalisib treatment [435]. More studies are needed to assess cardiovascular risk.
IFN-α	**Positive:** A prospective observational study of patients treated with (LPV/RTV and CQ and IFN-α

	saw a higher proportion of patients recovered from COVID-19 than those treated with (LPV/RTV and CQ and without IFN-α2b. Patients who did not receive IFN-α2b had higher incidence of comorbidities, such as high blood pressure, ischemic heart disease, and diabetes mellitus, but were also older than those receiving IFN-α2b [436].<br>Two patients with CHF secondary to enteroviral myocarditis were treated successfully with IFN-a, however this study is small, observational, and was not randomized [437].<br><br>**Negative:** A review of 44 cases of interferon induced toxicity identified some cardiovascular adverse effects including cardiac arrhythmia, dilated cardiomyopathy, and symptoms of ischemic heart disease, including myocardial infarction and sudden death [438].
Ivermectin	**Negative:** Tachycardia, hypotension, and prolonged PR interval have been observed [439, 440].
Leronlimab (PRO 140)	**Limited Information:** Leronlimab's effect on the heart has not been studied. However inhibiting CCR5 signaling could have beneficial effects on the heart [441].
Lopinavir/ Ritonavir (LPV/RTV)	**Neutral:** A trial of LPV/RTV in adults hospitalized with severe COVID-19 did not accelerate clinical improvement but it did not show significant QTc prolongation or serious arrhythmic events in either the patients treated with LPV/RTV or the standard care group [442].<br>A case study of a COVID-19 patient treated with LPV/RTV developed brady arrythmia which resolved when treated was stopped [443].<br><br>**Negative:** HIV protease inhibitor RTV has caused QT and PR interval prolongation [444, 445].<br>It induced endothelial cell injury at concentrations near clinical plasma levels which could have implications on cardiovascular complications. It caused endothelial mitochondrial DNA damage and necrosis [446].
Methyl Prednisolone (MP)	**Positive:** Has been shown to improve cardiac recovery after ischemic arrest [447].<br>Used successfully to treat myocarditis [448, 449].<br>A case study of patient with dyspnoea due to COVID-19 develop elevated cardiac enzymes, severe left ventricular dysfunction, and high inflammatory markers was successfully treated with intravenous immunoglobulin (IVIG) and MP [450].<br>A case study of a patient presented with SARS-CoV-2 related fulminant myocarditis treated with milrinone and MP reduced myocardial inflamation [451].<br><br>**Negative:** High-dose i.v. MP has been associated with atrial fibrillation [452].
Nafamostat (NM)	**Positive:** In an animal study NM treatment prior to ischemia and reperfusion found that it had cardioprotective effect of attenuating myocardial injury [67].<br><br>**Negative:** A case study reported a cardiac arrest caused by treatment of NM as an anticoagulant for hemodialysis [453].
Niclosamide	**Limited Information:** Used to treat tapeworm infection and potentially useful for treating some cancers [454]. It has not been investigated for its effect on the heart. It targets multiple signaling pathways including WNT signaling [455]. WNT signaling activity is not very active in the cardiovascular system of healthy patients but is activated in many pathologies of heart and blood vessels [456]. The salt version of this drug appears to inhibit artery constriction in rats [457].
Nitazoxanide	**No effect:** NTZ did not prolong QT interval in healthy male and female volunteers [458].<br>Little cardiovascular effects observed in a rat study [459].

Nitric Oxide (NO)	**Positive:** NO is a major signaling molecule in the cardiovascular system [460]. Improved NO production in the endothelium can decrease blood pressure via vasodilation [461].<br>NO has vasodilatory, anti-thrombotic, and anti-inflammatory effects on vasculature, but when availability of NO is decreased, it can cause endothelial dysfunction [462].<br>NO protects against ischemia-reperfusion injury [463].<br>An outpatient with vasoreactive idiopathic pulmonary arterial hypertension and COVID-19 infection recovered upon treatment with inhaled NO [464].<br><br>**Negative:** In autoimmune myocarditis, the presence of excess NO appears to lead to the progression of myocardial damage in rats [465].
Remdesivir (RDV)	**Limited information:** Originally developed and approved to treat Ebola. Cardiovascular toxicities have not been extensively studied [466, 467].<br><br>**Negative:** An Ebola patient developed hypotension followed by cardiac arrest [468].<br>There has been an instance of torsade de pointes requiring cardiac resuscitation reported with RDV in a COVID-19 patient [469].<br>In a case study of 4 COVID19 patients, one patient treated with RDV was prematurely discontinued because of a torsade de pointes requiring cardiac resuscitation [470].<br>In another case study of 2 COVID-19 patients developed sinus bradycardia or QT prolongation when treated with RDV which reversed when discontinued [471].
Ruxolitinib (RUX)	**Positive:** A chemotherapy drug. Improved MF-associated pulmonary hypertension [472].<br>In a review and analysis of studies and trials of MF patients treated with ruxolitinib indicate that RUX was not associated with the deaths of patients from myocardial infarction or cardiac arrest [473].<br>A prospective, multicenter, controlled, randomized, single-blind study of patients with severe COVID19 treated with RUX had faster clinical improvement, however one patient developed grade 3 hypertension which was transient and reversible. Serious adverse events, e.g. acute HF, occurred only in the control group [81].<br><br>**Negative:** A case report showed treatment with RUX reduced cardiac function (i.e., left ventricular dysfunction) [472].
Sarilumab	**Limited Information:** Sarilumab's effect on the heart has not been studied. IL-6 signaling can have either beneficial or harmful effects on the cardiac system depending on the kinetics of the host response. Increased IL6R protein levels are associated with coronary heart disease (CHD) and targeting IL-6 receptor antagonists could be beneficial [474].
Sirolimus (Rapamycin)	**Positive:** Sirolimus is an immunosuppressive drug. Although MTOR inhibitors could be beneficial to the heart [475].<br><br>**Negative:** Sirolimus has been associated with significant increases in total cholesterol and triglyceride levels [476].<br>Sirolimus is associated with venous thromboembolism in cardiac transplant recipients [477, 478].
Sofosbuvir	**Negative:** Sofosbuvir has been associate d with worsening of left ventricular function in a longitudinal study of HCV patients [479].<br>In a cohort study of 415 HCV patients, 5 patients developed cardiac arrhythmia [480].<br>Sofosbuvir alone has negligible effect on the cardiomyocyte electrophysiology while amiodarone

	and sofosbuvir together did affect cardiomyocyte electrophysiology [481, 482].<br><br>**Neutral:** Minimal cardiovascular risks observed in treating hepatitis C viral infection in patients with severe renal insufficiency [483]. Nonetheless, there was one cardiac-related event that occurred in a chronic hepatitis C patient with cirrhosis and pre-existing CHF [484].
Statins	**Positive:** Statins decreased the levels of atherogenic lipoproteins and CRP, and these decreases are associated with slower progression of atherosclerosis [485].<br>Used to reduce cholesterol levels, it also has anti-inflammatory effects which contributes to their beneficial effects on the cardiovascular system [486].<br>Although statin appear to reduce severity of experimental myocarditis in animal studies, there are no clinical studies on the use of statins for myocarditis [487].<br>In-hospital use of statins or statins plus ACE inhibitors or ARBs for COVID-19 patients did not increase the risk of cardiac injury [488].
Stavudine	**Mixed:** Treatment with stavudine was associated with myocardial infarction in one study that found a significant increase in myocardial infarction and stroke, while several other studies did not find a significant increase in these cardiac events associated with the drug [489].
Tenofovir	**Positive:** TDF is associated with a decreased risk of HF in HIV patients [490] which could be related to its potential lipid-lowering effects.<br><br>**Negative:** Used to treat hepatitis B and HIV infection. TAF is associated with an increased risk of HF in HIV patients [491].<br>TDF/emtricitabine was associated with a higher likelihood of atherosclerosis in HIV patients [492].<br>An episode of syncope was reported while treating a HIV patient with tenofovir, emtricitabine and nevirapine [416].
Tetrandrine (TET)	**Positive:** TET is a calcium channel blocker with antifibrotic effects, preventing human cardiac myofibroblast activation and suppressing collagen synthesis in vitro and limiting myocardial fibrosis in Dahl salt-sensitive hypertensive rats [493].<br>TET pretreatment had protective effects on isoproterenol-induced myocardial infarction in an animal model. TET lowered the levels of c-troponin I (a cardiac necrosis marker) and malondialdehyde (marker of oxidative stress) in the isoproterenol treated animals [494].<br>It improved ischemia/reperfusion lesions in rat myocardium by inhibiting neutrophil activation and infiltration, and ROS production [495].
Thalidomide (THD)	**Positive:** Chronic HF patients treated with THD increased cardiac performance (i.e., increased left ventricular ejection fraction) [496].<br><br>**Mixed:** THD has anti-inflammatory (decreases neutrophil count) and proinflammatory (increases TNF-α) effects on chronic HF patients [497]. Inflammatory cytokines, including TNF-α and IL-6 are associated with HF [498].<br><br>**Negative:** THD can contribute to bradycardia [499].<br>May induce cardiotoxicity in multiple myeloma patients [500]..
Tocilizumab (TCZ)	**Positive:** This drug is a humanized IL-6 receptor antibody and used to treat RA [501].<br>IL-6 receptor signaling adversely affects CHD and inhibiting IL-6R signaling could be beneficial.<br>In a phase 3 clinical trial study of TCZ safety for RA, myocardial infarction was lower in the treated group as compared with the control group [127].

	A patient in critical condition with COVID-19 related myocarditis was treated with TCZ, an aldose reductase inhibitor, and corticosteroids. The patient made a full recovery [502].<br>Improves endothelial function but increases total cholesterol and LDL levels [503].<br><br>**Negative:** Major adverse cardiovascular events in RA patients treated with TCZ were higher than with etanercept (TNF-α inhibitor), but not meaningfully higher [504], it did increase blood pressure and LDL [505].<br>A study of 43 patients with severe COVID-19 pneumonia treated with TCZ indicated ~7% of the patients developed candidemia, with one developing both endophthalmitis and endocarditis [506].<br>In a prospective, open-label, randomized clinical trial of hospitalized patients with COVID-19 pneumonia in Italy, TCZ increased CRP levels [507].
Umifenovir (Arbidol)	**Positive:** Umifenovir has a safe cardiac profile for treatment of influenza [508].<br>Arbidol reduced the viral load in the heart and lungs of mice with the Coxsackievirus B5 infection [509].
Valganciclovir/ Ganciclovir	**Positive:** When used as a preventative measure, a protective effect against CMV infection was observed. In addition, the survival of older patients with cardiovascular comorbidities was increased [510].<br>Valganciclovir was used to resolve myocarditis caused by CMV infection in a patient [511].
Zidovudine (AZT)	**Positive:** A case study suggests that AZT may be beneficial in treating HIV-associated myocarditis [512].<br><br>**Negative:** Treatment of AZT increased blood pressure and promoted cardiovascular damage. However, these negatives were inhibited by vitamin C [513]. Study was done for 8 months on rats.<br>An association between AZT and the development of cardiomyopathy was reported in patients with HIV, both in children [514] and adults [515]. However, a more recent study suggests immunodeficiency may be influencing the development of cardiomyopathies [516].

Supplementary Table 5: Drug Effects on the Kidneys

Drugs	Kidney Effects
Abacavir	**Negative:** Used to treat HIV. Some evidence of patients treated with tenofovir and indinavir, and to a lesser extent abacavir, had a higher risk for chronic kidney disease (CKD). [517]
Anakinra	**Positive:** Administering IL-1 receptor antagonist (IL-1ra) has been shown to attenuate tubular necrosis in a rat model [518].<br>Ischemia/reperfusion injury in organ transplantation or hemorrhagic shock causes renal dysfunction. Anakinra was found to be safe in patients going through renal transplantation [519].<br><br>**Mixed:** Anakinra reduced renal fibrosis but exacerbated renal hypertrophy and had modest effects on renal inflammation and leukocyte infiltration in mice with one kidney, deoxycorticosterone acetate (DOCA) implantation, and salt induced hypertension [520].
APN01 (rhACE2)	**Positive:** rhACE2 alleviated angiotensin II-mediated renal fibrosis and structural injury in atherosclerosis-prone ApoE-mutant mice. rhACE2 prevented Ang II-mediated increases in superoxide production and mTOR and extracellular receptor kinase (ERK)1/2 phosphorylation signaling, and also reduced TGF-β1, collagen I and fibronectin mRNA levels [521].<br>rhACE2 ameliorated albuminuria, and reduced kidney fibrosis in mouse models of systemic renin angiotensin system activation. It reduced the AKT (aka protein kinase B) and ERK phosphorylation, which helped protect renal cells from Ang II-induced injuries [522].<br>Human soluble rhACE2 can inhibit SARS-CoV-2 infection of engineered human blood vessel organoids and human kidney organoids [8].
Ascorbic Acid (AA)	**Positive:** However, L-AA dose-dependently was found to protect renal tubular cells from chromium intoxication-induced oxidative stress and cell death in a cell study. Chromium ($K_2Cr_2O_7$)-induced ROS production, apoptosis, and autophagy was suppressed with L-AA treatment [523].<br><br>**Negative:** AA breaks down ultimately to oxalate. Supersaturated calcium oxalate in the tubules preferentially deposit as crystals in damaged tubular epithelium, where they are thought to cause both obstruction and apoptosis of tubular epithelial cells [524]. In a case study, excessive vitamin C was believed to contribute to acute tubular injury (ATI) and oxalate nephropathy in two patients with slowly resolving acute kidney injury (AKI) after ARDS due to COVID-19 [525].<br>There are several case studies that have associated high levels of AA and acute or chronic renal failure [526-529].
Azithromycin (AZM)	**Neutral:** In a clinical trial no significant increase in acute kidney failure between the control and AZM group in severe COVID-19 patients in Brazil [155].<br>A population-based cohort study found a small but statistically significant greater 30-day risk of hospitalization with AKI for patients co-prescribed calcium-channel blockers and clarithromycin as compared with AZM [530].<br><br>**Negative:** In rats, AZM appears to induce phospholipidosis, as the of kidney damage was correlated with the spatial distribution of AZM accumulation, its metabolites, and phospholipids (phospholipids-phosphatidylinositol, phosphatidylethanolamine, and possibly didocosahexaenoyl (C22:6)-bis(monoacylglycerol) phosphate) [531].
Baloxavir	**Limited information:** Renal impairment due to baloxavir/marboxil has not been evaluated [532].

Baricitinib	**Positive:** In treating diabetic kidney disease (DKD), baricitinib significantly reduced albuminuria, the most widely accepted clinical biomarker for DKD, in study participants at high risk for disease progression. This JAK1/JAK2 inhibitor shows potential for treating DKD [533].
Brincidofovir/ Cidofovir	**Positive:** A case study found brincidofovir successfully treated adenoviral infection in a kidney transplant patient that developed renal insufficiency with cidofovir [166].<br><br>**Negative:** Brincidofovir induced severe AKI in a heart and a kidney organ transplant patient with CMV infection, which was reversible [534].<br>In a retrospective study of pediatric patients with adenovirus infection, cidofovir was associated with greater increases in creatinine levels and AKI [535].<br>Prolonged use is known to cause severe nephrotoxicity in a monkey study [536].
Camostat Mesilate (CM)	**Positive:** CM appears to be beneficial for treating diabetic nephropathy [537].<br>CM attenuates the progression of CKD in an adenine-induced CKD rat model by decreasing the expression and protein levels of fibrotic markers, and the accumulation of oxidative stress [538].<br>CM appears to have an antihypertensive effect on Dahl salt-sensitive rats fed a high salt diet, which contributed to renoprotective effects [539].<br>CM exerted renoprotective effects on kidney injury induced by aldosterone and salt in Sprague–Dawley rats by suppressing inflammatory and pro-fibrotic cytokine expression, ROS production and TGF-β signaling. CM inhibited renal plasmin and suppressed plasmin induced pro-fibrotic (collagen I/III) and inflammatory genes (e.g. TGF-β1, TNF-α) in cultured renal fibroblasts. CM attenuated urinary protein excretion, glomerulosclerosis and tubulointerstitial fibrosis [540].
Chloroquine (CQ)	**Negative:** Renal toxicity has been observed with long-term use of CQ in rats [541].<br>In a case study, a patient with RA developed muscle weakness and renal insufficiency with CQ, which improved eventually upon cessation of the drug [542].<br>Furthermore, serious complications occurred in a patient with underlying end-stage kidney failure who got CQ [543].
Colchicine	**Positive:** There is some evidence that colchicine could be beneficial in renal fibrosis, although more clinical trials are needed [544].<br>Similarly in animal studies, colchicine was found to mitigate renal fibrosis in a murine unilateral ureteral obstruction model [545].<br><br>**Negative:** Colchicine was associated with kidney injury in a diabetic with stage 4 chronic kidney disease and a renal transplant recipient [546].
Corticosteroids	**Negative:** In a retrospective study, corticosteroids were associated with severe adverse effects (e.g., all-cause mortality, severe or fatal infection, gastrointestinal hemorrhage or gastrointestinal perforation, new-onset diabetes mellitus or cataract, fatal/nonfatal myocardial infarction, fatal/nonfatal stroke, and heart failure) in patients with IgA nephropathy [547].
Dexamethasone (DEX)	**Positive:** DEX appears to reduce the incidence of severe AKI after cardiac surgery, notably in patients with advanced CKD [548].<br>Aged human kidneys form tertiary lymphoid tissue (TLT). In a kidney injury mouse model administering DEX halted TLT formation and attenuated renal inflammation and fibrosis [549].
Disulfiram (DS)	**Positive:** Elevated transglutaminase 2 (TG2) activity in the extracellular matrix is associated with renal fibrosis [550, 551]. DS was shown to be an irreversible inhibitor of human TG2, but further investigation is needed [552].

Dociparstat sodium (DSTAT, aka ODSH)	**Limited information:** The effect of ODSH on the kidneys has not been studied. However, CXCL12/CXCR4 levels are upregulated in kidneys of lupus mouse model and contribute to leukocyte trafficking into the kidneys which may mediate nephritis [553]. As a CXCL12/CXCR4 inhibitor, DSTAT could mitigate renal pathology in lupus nephritis.
Emtricitabine	**Positive:** In a study of 2499 HIV patients, emtricitabine with TDF was associated with a very mild nonprogressive reduction in creatinine clearance [554].<br><br>**Negative:** An HIV patient taking Genvoya (elvitegravir/cobicistat/emtricitabine/tenofovir alafenamide) with simvastatin developed severe rhabdomyolysis-induced anuric AKI. This is most likely due to Genvoya interfering with metabolism of simvastatin via the CYP3A4 pathway [555].
Entecavir (EVT)	**No effect:** As compared to tenofovir, which decreased the estimated glomerular filtration rate (eGFR), EVT did not cause a significant change in eGFR in chronic hepatitis B patients [556].<br>No negative effects on the kidney were reported in a rat study [557].
Epoprostenol (EPO, synthetic equivalent of prostacyclin)	**Mixed:** EPO's effect on the kidneys has not been studied, however, prostacyclin infusion was found to increase renal plasma flow, and plasma levels of atrial natriuretic peptide (ANP) [558]. ANP may lead to CKD [559]. It also increased renal levels of angiotensin (ANG) II in healthy controls [558]. Increases in ANG II have been reported in different types of renal diseases [560].
Famotidine	**No effect:** Famotidine does not appear to interfere with clearance of other basic drugs in patients with chronic renal insufficiency [561].
Favipiravir	**No effect:** Patients with COVID-19 treated with favipiravir as compared to arbidol did not significantly improve clinical recovery rate but favipiravir did significantly shorten the latency to relief for fever and cough [218].<br>The results for favipiravir on the kidney is limited. Favipiravir, an RNA-dependent RNA polymerase (RDRP) inhibitor, is predominantly excreted through the urine; thus, patients with eGFR<20 ml/min per 1.73 m^2 are excluded from clinical trials. Favipiravir used in Ebola infection was not associated with a significant decrease in creatine kinase level [562].<br><br>**Limited information:** A case report of SARS-CoV-2 infection in a kidney transplant recipient treated with HCQ and AZM and favipiravir, followed by TCZ had fatal outcomes [563].<br>In contrast, another kidney transplant patient with COVID-19 pneumonia was treated with several drugs including favipiravir and TCZ had a favorable outcome [564].
Hydroxy Chloroquine (HCQ)	**Positive:** This antimalarial retards the onset of renal damage. HCQ can protect from renal damage in lupus patients. Inhibits intracellular TLRs and the movement of nuclear material into cells, which prevents the formation of auto-antibodies and activation of plasmacytoid dendritic cells. This decreases production of IFN-α. [565-573]<br>In a mice model of ischemia/reperfusion injury HCQ attenuated renal injury by reducing inflammation by inhibiting inflammasome activation [574].<br>HCQ protected against renal damage in SLE patients [565].
IC14	**Limited information:** The effect of IC14 on the kidneys has not been studied. However, CD14 levels correlated with polycystic kidney disease (PKD) severity in *cpk* mice, suggesting a potential for anti-CD14 therapy in PKD [575].
Idelalisib	**Positive:** A case study of a patient with Waldenström's macroglobulinemia (a rare B-cell lymphoma) and refractory to first-line treatment with rituximab, cyclophosphamide, and DEX, presented with

	acute renal failure had improvement in his clinical status when treated with idelalisib [576].<br><br>**Negative:** Adverse renal effects of idelalisib on a chronic lymphocytic leukemia patient included hypokalemia, AKI, and hyponatremia but nonetheless had fewer adverse effects than ofatumumab, alemtuzumab and ibrutinib [577].
IFN-α	**Positive:** IFN-α therapy for metastatic renal cell carcinoma (RCC) showed a modest survival as compared with placebo [578].<br><br>**Negative:** IFN-α treatment has been associated with focal segmental glomerulosclerosis [579]. Systemic IFN-α treatment of a lupus prone mice model led to the development of glomerulonephritis and accelerated kidney disease [580].
Ivermectin	**Positive:** Ivermectin inhibits proliferation and promotes apoptosis in RCC cells and inhibits RCC tumor growth in vivo by inducing mitochondrial dysfunction (i.e.decreasing mitochondrial membrane potential, mitochondrial respiration and ATP production) and oxidative stress. Ivermectin is preferentially cytotoxic to RCC cells compared to normal kidney cells [581].
Leronlimab (PRO 140)	**Limited information:** The effect of leronlimab on the kidneys has not been studied. CCR5 is a receptor for the C-C chemokine RANTES and is expressed in inflammatory kidney diseases. A study found an association between CCR5-positive T cells and renal insufficiency in glomerular diseases and progression of renal disease [582].<br>In a study of 413 patients, those with high-sensitivity C-reactive protein (hsCRP) > 10 mg/L and not carrying a deletion allele for CCR5 had the worst survival. The results suggest deletion of CCR5 protected dialysis patients from inflammation associated mortality. A dual CCR2/CCR5 antagonist was found to have antifibrotic and anti-inflammatory effects on the kidney in a mouse model [583]. Therefore, leronlimab could be beneficial by inhibiting CCR5 for fibrotic diseases.
Lopinavir/ Ritonavir (LPV/RTV)	**Negative:** LPV/RTV slows the clearance of TDF in HIV-infected patients [584].<br>Interacts with renal transport of organic anions, leading to proximal tubular intracellular accumulation of TDF [585].<br>RTV has been associated with AKI [586].<br>LPV/RTV have been associated with an adverse effect on renal function in patients without pre-existing renal impairment [587].<br>RTV, LPV, and other protease inhibitors (lamivudine, tenofovir, and didanosine) can interact with the renal transport of organic anions, leading to proximal tubular intracellular accumulation of tenofovir in HIV patients. The clinical and biological characteristics are consistent with Fanconi syndrome–type tubulopathy [585].
Methyl Prednisolone (MP)	**Positive:** MP reduces LPS induced expression of fractalkine through the NF-κB pathway. Fractalkine is involved in the development of human lupus nephritis [588].<br><br>**Negative:** MP has been associated with transient renal failure [589].
Nafamostat (NM)	**Positive:** NM, a synthetic serine protease inhibitor, is used as an anticoagulant during continuous renal replacement therapy (RRT). In a prospective study NM did not increase the risk of bleeding during continuous RRT in critically ill patients with AKI patients at high risk of bleeding tendencies [590].<br><br>**Negative:** Hyponatremia and hyperkalemia rates were higher in the NM than the gabexate mesilate treated group [591].

	A cell study suggested that NM-induced decrease in prostasin (an important physiologic regulator of sodium reabsorption in the kidney and is involved in the development of salt-sensitive hypertension), which leads to a decrease in amiloride-sensitive sodium channels activity, and could account for the hyponatremia or hyperkalemia [592].
Niclosamide	**Positive:** Niclosamide inhibits proliferation and migration and induces apoptosis in human RCC cells [593].<br>It reduced proteinuria, glomerulosclerotic lesions, and fibrosis in a mouse model of adriamycin nephropathy [594] and improved kidney injury in db/db mice [595].<br>It inhibited breast cancer metastases to the kidney in mice [596].<br>Delivery of niclosamide via particles of human lysozyme as a carrier molecule reduced viral load in the kidneys of hACE2 transgenic mice infected intranasally with SARS-CoV-2 [270].
Nitazoxanide (NTZ)	**Positive:** NTZ successfully treated norovirus- and sapovirus-related symptoms in renal transplant patients [597].<br><br>**Negative:** It was found to increase markers for kidney injury in rats [598].
Nitric oxide (NO)	**Positive:** NO is a vasodilator mainly through the eNOS/NO/cGMP pathway. Renal diseases are often associated with NO deficiency [599].<br>In ischemia/reperfusion induced acute renal failure, NO was protective against kidney injuries [600].<br><br>**Negative:** In contrast, inhaled NO could increase the risk of renal impairment in adult patients with acute respiratory distress syndrome and acute lung injury [600].
Remdesivir (RDV)	**Positive:** In a single-center study, RDV was tolerated well in patients with CKD and AKI, and many of the patients were on hemodialysis. More research is needed for conclusive results [601].<br><br>**Negative:** Used to treat COVID-19, however, patients with severe AKI and end stage kidney disease (ESKD) were excluded from all RDV trials on the basis of eGFR cutoffs (either 50 or 30 ml/ min per 1.73 m^2). Intravenous preparation of RDV uses SBECD as a carrier. Accumulation of SBECD is associated with renal tubule obstruction in animal studies, but is easily removed by continuous RRT and hemodialysis [602].<br>In support, an analysis of World Health Organization (WHO) databases found significant pharmacovigilance signals of nephrotoxicity associated with RDV [603].
Ruxolitinib (RUX)	**Positive:** In a retrospective analysis of 100 patients with previously untreated primary MF, RUX was associated with improved renal function as compared to control [604].<br><br>**Negative:** However, RUX also has been associated with renal insufficiency in some patients with MF [605].
Sarilumab	**Limited information:** Sarilumab is a human monoclonal antibody against IL-6 receptor-α approved for the treatment of RA [606]. Its effect on the kidney has not been studied. Given the relevance of IL-6 is a prognostic indicator of renal diseases, blocking IL-6 receptor-α could be a possible therapy for renal diseases.
Sirolimus (Rapamycin)	**Positive:** Inhibiting mTORC1 by rapamycin has renoprotective effects on diabetic nephropathy progression in animal models of type 1 and type 2 diabetes [607-609].<br>In mouse kidney fibroblasts, rhACE2 and mTOR inhibitor rapamycin exposure prevented superoxide generation and mTOR activation induced by angiotensin II [521].

	Sirolimus decreased kidney transplant rejection comparable to cyclosporin. Unlike cyclosporin, it did not cause nephrotoxicity [610].<br><br>**Mixed:** mTOR signaling in glomerular disease is heterogeneous and the effect (positive or negative) of rapamycin is context-dependent [611].<br><br>**Negative:** In some patients with chronic glomerulopathies, rapamycin was found to cause nephrotoxicity [612].
Sofosbuvir	**Mixed:** Patients with HCV treated with sofosbuvir showed viral clearance but had higher incidences of anemia and worsening renal function [613].
Statins	**Positive:** Statins effect on the kidney is context dependent. It appears to prevent AKI after cardiac surgery and reduce major cardiovascular events and mortality in CKD [614].<br>Statins protect against nephrolithiasis (kidney stone formation), and not through its lipid-lowering mechanism [615].<br>In-hospital use of statins or statins plus ACE inhibitors or ARBs did not increase the risk of kidney injury of COVID-19 patients [315].<br><br>**Negative:** Used to treat hyperlipidemia. There appears to be an association between statin and increased incidence of acute and chronic renal disease as well as tubulo-interstitial nephritis, and statins' effects in patients with CKD remain uncertain [614].
Stavudine	**Negative:** In a case study, stavudine was associated with renal tubular injury [616].<br>An AIDS patient on stavudine and lamivudine was associated with lactic acidosis [617].<br>Another case study, an HIV patient developed lactic acidosis and renal failure secondary to acute hepatic failure [618].
Tenofovir	**Negative:** Tenofovir [619] and TDF used to treat HIV is associated with nephrotoxicity and involves mitochondrial abnormalities [620].<br>Tenofovir increases CKD [621]. A nonrandomized large cohort was associated with a higher incidence of CKD with increasing exposure to tenofovir [622].
Tetrandrine (TET)	**Positive:** TET is an urate-lowering drug and hyperuricemia could influence on eGFR. Evidence suggest that hyperuricemia has a damaging effect on kidney function, but its benefits maybe context dependent, with less conclusive benefits in patients with high blood pressure [623] and proteinuria [624].<br>In RCC, proteinuria predicts decline in kidney function [625]. Overexpression of transient receptor potential cation channel protein 6 (TRPC6) caused proteinuria in healthy mice [626]. TET protected podocytes by inhibiting TRPC6-overexpression-induced intracellular Ca^{2+} influx, inhibiting podocyte apoptosis and enhancing podocyte growth via inhibition of the RhoA/ROCK pathway [627].<br>TET treatment decreased growth and viability of RCC cells. TET induced G1 cell cycle arrest and apoptosis in RCC cells, respectively, by increasing expression of cell cycle regulatory protein $p21^{WAF1/CIP1}$ and $p27^{KIP1}$, and activating caspase-8, caspase-9, and caspase-3 in TET-treated RCC cells [628]. TET inhibited RCC migration and invasion by downregulating the expression of NF-κB, MMP-9, p-AKT, p-PI3K and p-PDK1 [629].
Thalidomide (THD)	**Positive:** Used to treat side effects caused by chemotherapy. Renal failure is a concern with multiple myeloma patients. THD, alone or in combination with DEX improved renal function [630].<br>Treatment with THD prevented cisplatin-induced increase in blood urea nitrogen (BUN) and creatinine levels. Cisplatin-induced cytokines (TNF-α, IL-1β, IL-6, TGF-β1) were reduced by THD in

	a dose-dependent manner, while IL-10 was elevated. THD prevents renal dysfunction induced by cisplatin by reducing NO-mediated cytotoxicity [631].<br><br>**Negative:** Nephrotoxicity associated with this drug included kidney stones and decreased GFR in clinical practice [632].
Tocilizumab (TCZ)	**Positive:** Evidence indicates the relevance of IL-6 as a prognostic indicator and blocking IL-6 receptor-α as a therapy for renal diseases [633]. TCZ reduced proteinuria and stabilized renal function in patients with Multicentric Castleman's disease (MCD) which overproduces IL-6 [634, 635].<br>TCZ reduced proteinuria and stabilized amyloid deposits in a patient with nephrotic syndrome due to rapidly progressing AA amyloidosis [636].<br>Patients with end-stage renal disease with COVID-19 have been successfully treated with TCZ [637, 638].<br><br>**Neutral:** Kidney transplant recipients who received TCZ for severe COVID-19 have shown high mortality (possibly due to the TCZ group was more critically ill), and when compared to matched control cohort group TCZ did not have significant beneficial response (e.g. mortality) [639].<br>The effect of TCZ on kidney transplant patients with COVID-19 pneumonia requires further controlled studies [640].
Umifenovir (Arbidol)	**Limited information:** It is a broad-spectrum antiviral [641]. The effect of umifenovir/arbidol on the kidneys has not been investigated.<br>In an in-hospital study of COVID19 patients, there was no significant difference between patients with and without AKI treated with umifenovir [642], ganciclovir, interferon, lopinavir/ritonavir, oseltamivir, and ribavirin.
Valganciclovir/ Ganciclovir	**Negative:** Used as anti-CMV therapy in kidney transplant recipients. Patients developed acidosis, which was compounded with active viral infection [643].<br>In kidney transplant patients preemptive post valganciclovir prophylaxis treatment had limited impact on preventing late-onset CMV disease [644].
Zidovudine (AZT)	**Positive:** HIV transgenic mice treated with AZT had significant reductions in glomerulosclerosis, interstitial fibrosis, and tubular atrophy, but not cortical inflammation or tubular cast formation. However, it is unclear if AZT had a protective effect independent of darunavir [645].<br><br>**Negative:** In several case studies, AZT treatment of HIV patients was associated with fatal lactic acidosis [646].

Supplementary Table 6: Drug Effects on the Liver

Drugs	Liver Effects
Abacavir	**Negative**: Abacavir hypersensitivity is associated with abnormal liver enzyme levels [647].<br>Abacavir induces multiorgan toxicity in people who carry the HLA-B*57:01 allele [648].<br>Mice carrying this allele did not increase markers of liver injury unless also treated with CpG oligodeoxynucleotide [649].<br>In Hep3B and human liver tissue abacavir combined with acetaminophen (even at low doses) negatively impacted mitochondrial function and increased risk for liver injury, and is relevant to HIV patients taking NRTIs, given that HIV increases oxidative stress which can affect mitochondrial function [650].
Anakinra	**Positive:** Mice lacking the IL-1R1 receptor in liver cells were protected from D-galactosamine (D-GalN) and LPS induced liver failure. Anakinra, which blocks IL-1R1, prevents liver injury from D-GalN/LPS as determined by reduction in alanine aminotransferase (ALT) and aspartate aminotransferase (AST) levels [651].<br>Anakinra attenuated alcohol-induced liver inflammation, steatosis, and steatohepatitis in mice [652].<br><br>**Negative:** In a case study, a patient treated with anakinra for RA displayed elevated liver enzymes and hepatotoxicity [653].
APN01 (rhACE2)	**Positive:** Adeno-associated viral vector delivery of rhACE2 sustained overexpression and activity of ACE2 which reduced the ratio of Ang II to Ang-(1–7) and reduced hepatic stellate cell activation, oxidative stress, release of proinflammatory, and profibrotic mediators, and thereby inhibited liver fibrosis in three different models of liver disease in mice [654].
Ascorbic Acid (AA)	**Positive:** AA could protect the liver from arsenic induced lipid peroxidation by improving mitochondrial function and preventing DNA fragmentation likely due to its antioxidative properties [655].<br>A cross-sectional study suggests that AA could have an inverse relationship to non-alcoholic fatty liver disease (NAFLD) [656].
Azithromycin (AZM)	**Positive:** In cells, AZM showed anti-tumor effects and suppressed the proliferation of HepG2 cells by inducing apoptosis through both the extrinsic/intrinsic apoptotic pathways [657].<br><br>**Negative:** A patient developed cholestatic hepatitis after four days of therapy with high-dose AZM for the treatment of suspected bronchitis. This case study of AZM being used to treat bronchitis was associated with adverse hepatic reactions [658].<br>A review of publications in 2015 found that acute drug-induced liver injury was more severe and potentially fatal in patients with underlying liver disease than in patients without liver disease, and AZM increased the risk of hepatotoxicity in these patients [659].
Baloxavir	**Limited information:** Little evidence that baloxavir caused liver injury, however instances of elevated ALT levels have been observed [660].
Baricitinib	**No effect:** Used to treat RA. Patients with inadequate response to methotrexate, treated with baricitinib, increased LDL and HDL cholesterol levels compared with placebo. Increases in creatinine and creatine phosphokinase were non-clinically significant [661].

Brincidofovir/ Cidofovir	**Positive:** Brincidofovir and cidofovir are more effective than ribavirin at inhibiting pathology and replication of adenovirus (Ad)5 in the liver of hamsters with this infection. Both drugs reduce liver damage, morbidity, and mortality when given prophylactically or therapeutically [20, 662].
Camostat Mesilate (CM)	**Positive:** Improved metabolism in obese mice by increasing hepatic fibroblast growth factor 21 (FGF21) mRNA and plasma levels [663].<br>CM prevented rat hepatic fibrosis by reducing TGF-β generation and plasmin activity, which is protective [664].<br><br>**Negative:** However, a case report of drug-induced liver injury (DILI) due to CM, benzbromarone, or a combination of the two, was associated with autoimmune hepatitis [665].
Chloroquine (CQ)	**Positive:** CQ attenuated acute liver injury in carbon tetrachloride (CCl_4) treated mice by inhibiting inflammation and inducing apoptosis. Pretreatment with CQ down regulated hepatic tissue and serum levels of HMGB1, IL-6, and TNF-α. HMGB1-mediated the inflammatory responses. CQ pre-treatment also inhibited autophagy, and promoted apoptotic cell death of non-recoverable cells [666].<br><br>**Mixed:** Rats pretreated with CQ prevents ischemic liver damage early but aggravates liver damage in late phase of ischemia/reperfusion [667].
Colchicine	**Positive:** There is evidence suggesting colchicine could reduce some of the clinical manifestation of cirrhosis [668].<br>Treatment with colchicine prevents and delays the development of hepatocellular carcinoma (HCC) in patients with hepatitis virus-related cirrhosis. The protective mechanisms could be related to anti-inflammatory properties and inhibition of mitosis [669].<br>In a prospective, open-label, randomized and double blind clinical trial, hospitalized COVID-19 patients treated with colchicine + standard care (HCQ) reduced AST and ALT levels as compared to standard care [670].<br><br>**Negative:** Colchicine was found to contribute to liver damage in a heart transplant patient with chronic renal failure, which improved upon ceasing colchicine [671].<br>In another case report, it was found to contribute to hepatotoxicity and elevated liver enzymes which return to normal after discontinuing colchicine [672].
Corticosteroids	**Positive:** As an immunosuppressive agent, corticosteroids have been used to effectively treat DILI involving the autoimmune response (e.g. drug-induced autoimmune hepatitis) and to a lesser extent drug-induced hypersensitivity [673].<br><br>**Negative:** A case report of idiopathic thrombocytopenic purpura treated with corticosteroid developed non-traumatic osteonecrosis of the femoral head, elevated ALT and AST levels, and NAFLD [674].
Dexamethasone (DEX)	**Positive:** DEX improved liver function and survival of chronic hepatitis B patients with acute-on-chronic pre-liver failure (pre-ACLF) [675].<br>In rat studies, DEX has been shown to protect against drug-induced hepatotoxicity [676, 677].<br>DEX reversed a rat model of cholestasis-induced inflammation (TNF-α, IL-1β, IL-6) [678].<br><br>**Negative:** DEX has been shown to inhibit mitochondrial beta-oxidation, decrease hepatic triglyceride secretion leading to microvesicular steatosis [679] and to induce fatty liver in mouse models [680].
Disulfiram (DS)	**Positive:** DS influences *L*-tryptophan metabolism by increasing hepatic 3-hydroxykynurenine (3-HK)

	and 3-hydroxyanthranilic acid (3-HAA) levels, which possess alcohol-aversive and immunosuppressant properties [681].<br>DS has been investigated for treating HCC. DS combined with copper inhibited tumor progression in SCID mice but not in C57BL/6 mice, suggesting intact immune system compromises the therapeutic efficacy of DS plus copper [682].<br><br>**Negative:** There is evidence that DS could induce hepatotoxicity [683, 684].
Dociparstat sodium (DSTAT, aka ODSH)	**Limited information:** The effect of ODSH on the liver has not been studied. However, CXCL12/CXCR4 pathways involved in advanced liver disease are associated with hepatitis B and C [685]. As a CXCL12/CXCR4 inhibitor, DSTAT could mitigate the inflammation and fibrosis activated by these pathways.
Emtricitabine	**Negative:** Emtricitabine in chronic HBV has been associated with elevated ALT level [686] and exacerbation of chronic hepatitis B posttreatment, with one requiring liver transplantation [687].<br>A combination of efavirenz, emtricitabine, and tenofovir treatment of an HIV patient was associated with hepatotoxicity exhibiting extremely elevated aminotransferase levels, but resolved without acute liver failure [688].
Entecavir (EVT)	**Positive:** Reversal of cirrhosis has been observed with EVT, including decreased ALT and AST levels and improved liver function of chronic hepatits B and liver cirrhosis patients. $CD34^{+}$ cells increased in peripheral blood and liver tissues, suggested that these progenitor cells may migrate to the liver and help regenerate or repair the liver tissue [689].<br><br>**Negative:** Chronic hepatitis B patients with impaired liver function treated with EVT was associated with severe lactic acidosis [690].<br>Elevated ALT levels have resulted in chronic hepatitis B patients treated with EVT [691].<br>EVT is primarily used for hepatitis B treatment, and was found to have a higher risk of HCC as compared to tenofovir [692].
Epoprostenol (EPO, synthetic equivalent of prostacyclin)	**Positive:** EPO's effect on the liver has not been studied, however, prostacyclin (PGI2) and a prostaglandin analogue when administered early prevented galactosamine-induced hepatic necrosis and mortality in an animal model [693].
Famotidine	**Positive:** Overall has a good safety profile, it increases glycogen stores by inhibiting glycogen synthase kinase-3β and improves oral glucose tolerance (decreases serum glucose levels) in healthy volunteers [694].<br>A retrospective cohort study of hospitalized COVID-19 patients found that patients receiving famotidine had a statistically significant reduced risk of clinical deterioration leading to death or intubation [213].<br><br>**Negative:** There has been a case study in which a hepatitis C patient developed acute hepatitis associated with famotidine [695].
Favipiravir	**Positive:** Favipiravir and ribavirin exert a synergistic activity against HCV in human hepatoma cells [696]. Favipiravir may act as a mutagenic agent in influenza viruses in vitro [697] and viral RNA chain terminator [696]. Favipiravir used in Ebola infection was associated with a significant decrease in ALT and AST levels [562].
Hydroxy	**Positive:** When used to treat patients with SLE or RA, it reduced serum cholesterol and LDL levels

Chloroquine (HCQ)	compared to those treated with corticosteroids [698].<br>HCQ helped to improve obesity-associated insulin resistance and hepatic steatosis. In high fat diet (HFD)-induced obese C57BL/6J mice, HCQ reduced blood glucose levels, serum lipids, and hepatic steatosis. It also downregulated *de novo* lipogenesis genes and lipogenic enzyme levels in liver tissues, which had a hepatoprotective effect [699].<br>Antimalarials inhibit platelet aggregation and adhesion and reduce blood viscosity and thrombus size. They positively affect serum lipid profile and glucose concentration by preventing the formation of autoantibodies and immune-complexes, diminishing inflammation, and favorably acting at the vascular endothelial level [565]<br><br>**Negative:** HCQ caused a ten-fold increase in transaminase levels in a patient with ARDS caused by COVID-19 infection. Discontinuation of HCQ improved levels within 5 days [700].<br>HCQ-induced acute toxic hepatitis in a SLE patient, which resolved after ceasing HCQ and administering mycophenolate mofetil [701].
IC14	**Limited information:** The effect on IC14 on the liver has not been studied. However, CD14 levels in nonalcoholic steatohepatitis (NASH) patients correlate with liver inflammation, suggesting a potential for anti-CD14 therapy in NASH [702].
Idelalisib	**Negative:** Idelalisib, a PI3Kδ inhibitor, is associated with elevated transaminase levels in CLL patients [703]. In patients treated for CLL, idelalisib can cause immune-mediated severe hepatotoxicity (recorded in 54% of patients) [53]. The toxicity was immune-mediated, as evidenced by increased levels of chemokine ligand (CCL)-3 and CCL-4. Discontinuing the drug and/or beginning immunosuppressants remedied the complications [53].
IFN-α	**Positive:** IFN-α induced apoptosis independent of p53 in HCC cell lines. IFN-α induces the tumor suppressor promyelocytic leukemia protein (PML) and the tumor necrosis factor-related apoptosis-inducing ligand (TRAIL), which is mediated by PML [704].<br><br>**Negative:** Instances of primary biliary cirrhosis [705, 706], sarcoidosis [707, 708], autoimmune hepatitis [709, 710] and hepatic granulomas have been reported in hepatitis C patients treated with pegylated interferon therapy [711].
Ivermectin	**Negative:** A case of ivermectin induced severe hepatitis has been reported [712].<br>Ivermectin is metabolized via P4503A4 and if taken along with drugs like LPV/RTV or darunavir/cobicistat which inhibit P4503A4 will increase systemic exposure of ivermectin [713].
Leronlimab (PRO 140)	**Limited information:** The effect on leronlimab on the liver has not been studied. However, a study found inflammation in the early stages of HCV disease in patients carrying the CCR5Δ32 allele was significantly reduced as compared to control. CCR5Δ32 is a deletion mutation in the chemokine receptor CCR5 which results in significant reduction of CCR5 expression. The study suggests that CCR5 may be involved in the recruitment of immune cells in the liver and viral clearance during chronic HCV infection [714].
Lopinavir/ Ritonavir (LPV/RTV)	**Mixed:** LPV/RTV has been reported to elevate liver enzymes [715] and contribute to hepatotoxicity and full dose of RTV was associated with an increased risk of liver injury, while low dose RTV to LPV is not associated with greater risk of liver injury as compare with other HIV protease inhibitors [716]. See also umifenovir/arbidol.<br>Overdose can activate the ER stress signaling, inhibit proliferation of hepatocytes, induce apoptosis of hepatocytes through caspase activation, induce inflammatory reactions, and accelerate liver injury

	by aggravating oxidative stress [717].<br>RTV is associated with elevated liver transaminase levels in HIV-infected patients [718]. Using a PXR-humanized mouse model it was found that pregnane X receptor (PXR) modulates RTV hepatotoxicity through CYP3A4-dependent pathways [719].
Methyl Prednisolone (MP)	**Positive:** A study with rats found MP protected against hypoxia-induced liver injury by preventing mitochondrial membrane depolarization [720].<br><br>**Negative:** MP is associated with liver damage [721], including elevated ALT levels [722], fatty liver, steatohepatitis [723], and acute liver failure [724].
Nafamostat (NM)	**Positive:** Studies have found that NM has the potential to inhibit cancer progression, including HCC [725].<br>NM attenuates the acute decrease in blood pressure after reperfusion that frequently occurs during liver transplantation [726].<br>NM did not have an effect in reducing blood loss/bleeding but did suppress coagulation activity, as indicated by a reduced plasma levels of thrombin-antithrombin III complex and fibrinopeptide in liver surgery [727]. The is relevant since the level of thrombin-antithrombin comples and fibrinogen are higher in COVID19 patients than healthy controls and also higher in patients with thrombotic disease (who had higher case-fatality) than without thrombotic disease [728].
Niclosamide	**Positive:** Niclosamide salts, niclosamide ethanolamine (NEN) and niclosamide piperazine (NPP) attenuated hepatic steatosis by mitochondrial uncoupling and improving glucose tolerance in mice fed a HFD [729, 730].
Nitazoxanide (NTZ)	**Positive:** Chronic HCV treated with NTZ and peginterferon, with or without NTZ, showed normalization of their ALT level as did the patients treated with standard care (peginterferon alfa-2a and ribavirin). NTZ was associated with fewer relapses as opposed to standard care [731].<br>A case study suggests a potential role of this drug for treating HBV [732].<br><br>**Negative:** NTZ was found to increase ALT, AST, and alkaline phosphatase (ALP) levels in rats but these elevation decreased gradually with time [598].
Nitric Oxide (NO)	**Positive:** In animal models, breathing NO reduces hepatic ischemia/reperfusion injury [733].<br>In humans, iNO decreases hepatocyte apoptosis after liver transplantation [734].<br><br>**Mixed:** Exogenous NO plays a dual role in glycochenodeoxycholate-induced hepatocellular apoptosis. It can be anti-apoptotic at specific/limited concentration range and inhibited proliferative at high concentration, which may reflect the pathological role of iNOS/NO [735, 736].
Remdesivir (RDV)	**Positive:** RDV attenuated metabolic syndrome in HFD-induced NAFLD in mice. Liver weight, hepatic dysfunction and lipid accumulation was significantly reduced with RDV treatment. RDV supplementation suppressed the systemic and hepatic inflammation, reducing inflammatory cytokines and NF-κB activation. RDV reduced HFD-induced stimulator of interferon genes (STING) and its downstream factor IRF3 in livers of mice [737].<br>A liver transplant patient treated with RDV did not require ICU admission and was discharged after 5 days [738].<br><br>**Negative:** There is evidence that RDV increases ALT/AST levels indicating liver injury. There have been reports of elevated liver enzymes in COVID-19 patients treated with RDV. However the adverse effect did not progress to severe liver damage or liver failure in these patients whom did not have

	prior chronic liver disease [739].<br>RDV is not water soluble, so SBECD is used as a carrier. SBECD is associated with elevated transaminases, and accumulation of SBECD is associated with liver necrosis in animal studies [602].<br>In a study where RDV was used to treat COVID-19, one of the most common adverse events was increased hepatic enzymes [287].
Ruxolitinib (RUX)	**Positive:** A case study found RUX improved bilirubin levels in a patient with diagnosed primary MF and suspected of liver failure [740].
Sarilumab	**Mixed:** In some RA patients, sarilumab is associated with elevated levels of liver enzymes (ALT and AST) as well as total cholesterol, LDL, and HDL [741]. Despite the frequent elevations in liver tests, sarilumab has not been linked to instances of clinically apparent liver injury and most aminotransferase elevations were self-limited and without symptoms [742].
Sirolimus (Rapamycin)	**Positive:** Sirolimus reduced polycystic liver volume compared to treatment with tacrolimus in patients with autosomal dominant polycystic kidney disease by inhibiting mTOR in epithelial cells lining the liver cysts [743].<br><br>**Mixed:** Sirolimus has been associated with hepatotoxicity. It could have negative effects in liver transplant patients but in a prospective analysis it could be helpful in HCC [744].
Sofosbuvir	**Negative:** Case study of patients with hepatitis C cirrhosis developed severe DILI with administration of sofosbuvir and ribavirin [745].
Statins	**Positive:** In-hospital use of statins or statins plus ACE inhibitors or ARBs did not increase the risk of liver injury of COVID-19 patients [315].<br><br>**Negative:** Has well-researched lipid-lowering properties. Statins are associated with a very low risk of serious liver injury, although asymptomatic and transient elevation of aminotransferases have been observed [746].<br>It has been associated with cases of drug-induced liver injury [747].
Stavudine	**Negative:** According to the WHO database, stavudine is one of the five most common drugs associated with fatalities [748].<br>Stavudine was associated with hyperlactatemia [749], lactic acidosis [750], hepatic steatosis [751, 752], hepatotoxicity [618] and acute liver failure [753] in HIV patients.<br>In primary rat hepatocytes, stavudine impaired fatty acid (palmitate) oxidation and at very high doses led to cell death [754], and could lead to steatosis, and in the worst case liver failure [755].
Tenofovir	**Positive:** In a mouse study of CCl_4-induced liver fibrosis tenofovir treatment reduced liver fibrosis in this model [756]. TAF and TDF attenuated liver fibrosis and inflammation by downregulating the TGFβ1/Smad3 and NF-κB/NLRP3 signaling in the CCl_4-induced liver fibrosis mouse model [757].<br><br>**Negative:** In a patient with HBV infection, TAF [758] and TDF were found to induce liver failure or severe liver injury.
Tetrandrine (TET)	**Positive:** TET inhibited human HCC metastasis in vitro by inhibiting EMT; it also inhibited metastasis to the lung in mice [759].<br>TET has anti-cancer potential towards several cancers, including liver [760].<br>TET attenuates LPS-D-galactosamine-induced liver injury in mice [761].

	**Negative:** TET is a Chinese plant-derived alkaloid, and is an urate-lowering drug used to treat hyperuricemia. It could lead to decline in survival rate of L-02 (hepatic) cell line, increase the activities of AST, ALT, lactate dehydrogenase (LDH) and ALP [762].<br>Maximum dose exposure to TET caused elevations in ALT, AST, GGT, and LDH in mice [763].
Thalidomide (THD)	**Positive:** A patient with liver cirrhosis and advanced HCC went into remission with low dose THD therapy [764].<br>Used to treat endotoxin-induced liver injury and mortality in rats. By preventing the increase in $[Ca^{2+}]_i$ due to LPS, THD reduces the production of TNF-α by Kupffer cells. THD prevented LPS-induced mortality and liver injury [765].<br><br>**Negative:** There have been case reports indicating that THD could cause hepatotoxicity [766].<br>THD treatment of a patient with end-stage plasma cell leukemia developed hepatotoxicity [767].<br>In a study of a multiple myeloma patient treated with DEX and THD developed acute liver injury [768].<br>Other studies of multiple myeloma patients found that treatment of one patient with THD developed fatal fulminant hepatic failure (FHF) [769], while THD treatment induced hepatitis in another [770].
Tocilizumab (TCZ)	**Positive:** Although, mean value elevations of liver functional tests (AST, ALT and total bilirubin) in RA patients with inadequate response to methotrexate were observed in the TCZ group as well as in the control group, they were within normal range. These abnormalities were clinically not significant and no hepatitis was observed. Therefore, TCZ monotherapy appears to be tolerable in terms of liver function [771].<br>COVID-19 patients with elevated liver enzymes, IL-6 levels, and impaired lung function did not improve after HCQ, AZM and LPV/RTV treatment, were treated with TCZ, which improved their clinical condition and normalized their liver function within 3 weeks of treatment [772].<br>In a case report, TCZ had a positive outcome in a liver transplant recipient on hemodialysis with COVID-19 [773].<br><br>**Negative:** TCZ is associated with elevated ALT levels in RA patients [127, 774, 775].<br>There has been an instance of acute liver injury associated with TCZ [776].<br>A case report of a RA patient who was refractory to standard therapies was treated with TCZ and had normal liver enzyme levels but developed hepatosplenomegaly as a symptom [653].<br>A patient with COVID-19 treated with TCZ developed acute hypertriglyceridaemia [777].
Umifenovir (Arbidol)	**Negative:** A retrospective observational study of COVID-19 patients indicates that combined treatment of LPV/RTV and arbidol were risk factors for liver injury in non-critically ill patients. It is thought that the inhibiting metabolism of arbidol by LPV/RTV could contribute to drug-related liver injury [778].
Valganciclovir/ Ganciclovir	**Positive:** Used to treat adenovirus infection. Ganciclovir and valganciclovir inhibit Ad5 replication in hamster livers. It has efficacy both prophylactically and therapeutically. It inhibits Ad5 DNA synthesis possibly through inhibition of the adenoviral DNA polymerase [779, 780].<br><br>**Negative:** In a case study, ganciclovir was associated with acute liver injury in a lupus patient [781].
Zidovudine (AZT)	**Negative:** AZT treatment of HIV patients was associated with hepatic failure [646]. Long-term monotherapy with AZT is known to cause hepatotoxicity [782]leading to death [783].<br>AZT causes mitochondrial DNA depletion through inhibition of thymidine salvage [784].

Supplementary Table 7: Drug Effects on the Central Nervous System (CNS)

Drugs	Neurological Effects
Abacavir	**Positive:** In children with HIV, abacavir was able to reduce viral and pro-inflammatory marker concentrations in CSF, as well as improving the neuropsychological outcome of children under 6. [785]<br><br>**Negative:** In 6 separate case studies, abacavir caused psychiatric effects severe enough to discontinue treatment. These effects appeared within one month of beginning treatment and resolved after treatment was stopped. [786]<br>Abacavir may be linked to increased stroke risk, although this is somewhat controversial as studies both indicate it does [787] and does not [788] increase stroke risk.
Anakinra	**Positive:** Anakinra acts as an anti-inflammatory drug to reduce cytokine concentration in the brain in patients with various conditions (traumatic brain injury (TBI), stroke, and subarachnoid hemorrhage). [789]<br>In addition to the primary anti-inflammatory function, anakinra has been shown to reduce the frequency of seizures in several patients with intractable seizures. [790]<br>In patients with epileptic encephalopathy, anakinra reduced seizure recurrence in both the acute and chronic phases of the disease. This effect was due primarily to reduction of inflammatory factors within the brain. [791]
APN01 (rhACE2)	**Positive:** rhACE2's impact on the cardiovascular system may also extend to the nervous system, helping to prevent stroke in patients with COVID-19. [792]<br><br>**Neutral:** In a clinical study of healthy patients, no severe adverse effects, neurological or otherwise, were seen after administering rhACE2 daily for one week. 6 patients reported headaches, but not severe enough to discontinue treatment. [793]<br><br>Note: these sources referenced rhACE2, not APN01 specifically.
Ascorbic Acid (AA)	**Positive:** In diabetic rats, AA showed neuroprotective effects after ischemic injury. Since AA is lower in diabetic tissues, this may be more helpful for diabetic patients, since a normal level of AA may be sufficient to give this same effect. The improvement following AA administration was attributed to reduction of oxidative stress, which is made worse by diabetes. [794]<br>In rats with epilepsy induced by penicillin, AA reduced frequency and amplitude of epileptic activity. An experiment in rats suggests that AA required the endothelial-NOS/nitric oxide pathway to achieve this anti-seizure effect. [795]
Azithromycin (AZM)	**Positive:** In humans with cystic fibrosis, AZM activates anti-inflammatory macrophages following spinal cord injury. AZM also lowered neurotoxic potential of M1 macrophages. [796]<br><br>**Negative:** Negative neurological side effects of AZM seem to be relatively uncommon, most reports are case studies rather than large cohort studies. However, the side effects can be severe. One patient showed agitation and choreoathetoid upper body movements after two separate administrations of AZM. Other case studies discussed toxic catatonia, myasthenic crisis, hearing loss, and loss of taste. [797]
Baloxavir	**Neutral:** No effects on the CNS were detected in animal models, even at levels higher than the recommended dose in humans. [798]

Baricitinib	**Positive:** In patients with various interferonopathies (CANDLE, SAVI, and others), baricitinib improved clinical symptoms and reduced serum levels of inflammatory markers, including chemokines. This effect is achieved by inhibition of JAK1/2 signaling pathways. [799]<br>In a mouse model of HIV, the drug reversed cognitive deficits and decreased levels of pro-inflammatory markers and macrophages in the brain. [800]
Brincidofovir/ Cidofovir	**Positive:** For short-term (3 week) use in treating smallpox, brincidofovir was well tolerated, with no severe neurologic adverse effects reported - the most common were GI complaints, and the majority of all adverse effects were classified as mild. [801]<br>In HIV+ progressive multifocal leukoencephalopathy patients, cidofovir did not have an effect on mortality or residual disability. In vitro, brincidofovir is able to inhibit viral replication in human brain derived cell lines, but this benefit has not been observed in a clinical trial. [802]<br>Has led to neurological stabilization in idiopathic CD4 lymphocytopenia. [803]
Camostat Mesylate (CM)	**Positive:** In rats, CM prevented pancreatic pain induced by increased spinal Fos protein expression. A potential role in anti-inflammatory action in the spinal cord was also identified, also through inhibition of Fos expression. [804]<br>Since CM is able to penetrate into the CNS, it is possible that its activity of preventing viral entry into target cells may extend into the CNS as well as being demonstrated in other cell lines. [264]
Chloroquine (CQ)	**Positive:** In rats, CQ was found to be neuroprotective following TBI, reducing neuronal autophagy and cytokine expression. [805]<br>CQ and HCQ are effective for controlling neurological sarcoidosis. [806]<br><br>**Negative:** A 2020 review of the FDA's Adverse Event reporting system found that 12% of 4336 cases with exposure to CQ reported psychiatric adverse effects including amnesia, delirium, hallucinations, depression, and loss of consciousness. The diseases present in these cases were not specified. [807]
Colchicine	**Positive:** Colchicine can reduce stroke risk in patients with a history of coronary artery disease at low doses. [808]<br>In a TBI model with rats, colchicine increased neuronal survival over a 2-week period. This, in addition to the early neuroprotective effect, is suspected to be related to interaction with the iNOS pathway. [809]<br><br>**Negative:** When plasma levels are elevated above the given dose (due to altered renal function or other causes), colchicine can lead to myopathies, usually weakness, and axonal polyneuropathy. The myopathy usually resolved 3-4 weeks after the drug was stopped, while the neuropathy resolved more slowly. [810]<br>A patient on colchicine for 6 years developed colchicine myoneuropathy which improved upon discontinuation of the drug. [811]<br>Since colchicine can affect the absorption of vitamin B12, colchicine neuropathy may be due to vitamin B12 deficiency. [812]
Corticosteroids	**Positive:** Corticosteroids act as an anti-inflammatory and immunosuppressant agent, which can help to combat the inflammation in the brain caused by COVID-19. Glucocorticoids have been successfully used to treat encephalopathy caused by SARS in 5 patients in a hospital in Geneva. [813]<br><br>**Negative:** Continued high-dose use can lead to psychiatric side effects, including depression, mania, and psychosis (approximately 20% of patients). Usually these symptoms resolve after treatment is discontinued. [814]

Dexamethasone (DEX)	**Positive:** In rats, DEX prevents osmotic demyelination induced after rapid correction of hyponatremia. This effect could be due to DEX preventing BBB disruption. [815]<br>Treatment of tuberculosis infected human microglial cell culture with DEX reduced proinflammatory cytokine and chemokine concentration. [816]<br><br>**Negative:** In a clinical study of cerebral malaria, DEX was found to prolong recovery times and increase secondary adverse effects. Complications in DEX-treated patients included a longer comatose state, as well as non-neurological effects like pneumonia. [817]<br>Male infants treated with DEX for prevention of chronic lung disease had a higher incidence of neuromotor dysfunction than the control group. [818]
Disulfiram (DS)	**Negative:** Several sources indicate that DS can cause neuropathies in patients if used in high doses or for long periods of time. While the long period of time may not be an issue for COVID treatment, this is still something to consider. The symptoms do resolve after treatment is discontinued. [819]<br>The rate of occurrence of negative neurological effects increases with the duration of DS treatment. DS can decrease clearance of oxidatively metabolized drugs and increases the effect of both caffeine and alcohol. [820]
Dociparstat Sodium (DSTAT, aka ODSH)	**Positive:** In rats, ODSH acted as an anti-inflammatory without increasing the risk of hemorrhage following ischemic brain injury. This reduced damage from reperfusion injury common after ischemic stroke. [821]<br>After TBI in mice, ODSH reduced leukocyte accumulation in the brain, decreasing inflammatory effects. It also improved acute neurologic recovery as indicated by performance on water maze tests. [822]<br>CXCL12 affects communication between the immune system and the brain, allowing for coordination of activity, but resulting in an increased inflammatory response to mechanisms that target CXCR4. DSTAT inhibition of CXCL12 can help to reduce inflammation by this mechanism. [32]
Emtricitabine	**Negative:** When combined with tenofovir and raltegravir (a somewhat common combination for HIV treatment), emtricitabine inhibits neural progenitor cell proliferation in the mouse dentate gyrus. This leads to difficulty repairing damage caused either by treatments or by a disease. [823]<br>Emtricitabine has little mitochondrial toxicity or neurological effect but did have a higher rate of headache occurrence than lamivudine. [824]<br>Note: hard to find much clinically on emtricitabine alone, as it is so often used as one part of combination therapy for HIV/AIDS.
Entecavir (EVT)	**Negative:** While uncommon, neuropathy is a potential side effect of EVT if given for long periods of time, at high dosages, or to hepatitis B patients with other risk factors for neuropathies. [825]
Epoprostenol (EPO, synthetic equivalent of prostacyclin)	**Positive:** PG12 (aka EPO) reduced vasospasm induced by subarachnoid hemorrhage in a clinical study. The study suggests that low doses of PG12 could be a means of reducing cerebral vasospasms which do not respond to other means of treatment. [826]<br>PG12 increased oligodendrocyte precursor cell migration to the site of spinal cord injury, aiding in remyelination and repair of the injury site. [827]

Famotidine	**Positive:** Famotidine improved the condition of schizophrenic patients when administered in conjunction with their existing medications. [828]<br>In case studies of non-hospitalized COVID-19 patients, high-dose oral famotidine improved disease-related neurologic symptoms, including headaches and anosmia. [829]<br><br>**Negative:** Case study: woman treated with famotidine developed mania and seizures after administration. Manic symptoms continued for 3 months after discontinuation, requiring treatment with other psychiatric medications. While this was a singular case, this could potentially be an important adverse effect. [830]<br>In some patients, renal failure caused CSF levels of famotidine to be elevated above what was expected given the dose. These patients were observed to have mental deterioration and convulsions, suggesting that the adverse effects may be linked to unexpectedly high concentrations of famotidine within the CNS. [831]<br>In a mouse model, famotidine reduced the anticonvulsant function of antiepileptic drugs. [832]
Favipiravir	**Positive:** In treating rabies in mice, a very high dose (2-3X standard dosage) is required to achieve penetration into the central nervous system (CNS). At these high doses however, Favipiravir was successful in reducing viral replication within the CNS. Unless a high dose is given, Favipiravir will not have significant effects on the CNS due to low penetration. [833]<br>Favipiravir patients recovered more quickly from fever than lopinavir/ritonavir patients, and did not display significantly higher rates of adverse effects. [834]<br>In COVID-19 patients, Favipiravir showed only mild psychiatric effects, and had similar 7-day recovery rates (61% of patients) to umifenovir. Favipiravir had shortened latency to relief from cough and pyrexia compared to umifenovir. [218]
Hydroxy Chloroquine (HCQ)	**Positive:** In cell culture of human microglia, HCQ treatment administered before LPS activation reduced production of both pro- and anti-inflammatory cytokines. It also reduced demyelination in the spinal cord of mice. [835]<br>A review of WHO's database showed a very low incidence of psychiatric and neurological side effects (<1%, except headaches which was 2.8%). [836]<br><br>**Negative:** HCQ and CQ interact with lysosome activities which may contribute to retinopathy. [837]<br>Increased risk of retinopathy was observed with high dose and long-term (5+ years) use of HCQ. [838]
IC14	**Positive:** In humans, IC14 administration reduced LPS induced TNF and IL-10 levels. It also reduced the occurrence of symptoms including headache, fever, and vomiting. It significantly inhibited proinflammatory cytokine release that was induced by LPS injection. [226]
Idelalisib	**Neutral:** This study says that idelalisib and other PI3K inhibitors have low neurologic toxicity because they are unable to cross the BBB in a healthy animal model. [839]<br><br>**Negative:** One case study in a patient with Hodgkin's lymphoma found idelalisib to induce polyradiculopathy (demyelination that led to difficulty with movement and neuropathic pain), however this toxicity is unusual. [840]
IFN-α	**Negative:** Leukemia patients treated with IFN-α for an average of 26 months exhibited a variety of cognitive deficits, including verbal memory, speed of visual scanning and sequencing, and executive function. These deficits were more severe in the IFN treatment group compared to patients with leukemia who had not received IFN treatment. [841]<br>IFN-α treatment of 31 patients with HCV increased glutamate concentration in the dorsal anterior cingulate cortex and the basal ganglia, consistent with what is observed in bipolar depression. Depressive symptoms were observed in patients treated with IFN-α. [842]

	IFN treatment in cancer patients is associated with cognitive deficits and mood disorders. Risk factors include treatment duration of more than 2-3 months and high dose, as well as a history of neurologic or psychiatric illness. It is possible to alleviate these effects by using other pharmacologic treatments, including antidepressants, psychostimulants, and opioid agonists, simultaneously. [843]
Ivermectin	**Negative:** While negative neurological effects only occur in a small number of people, when they do occur they are severe: coma and death. There is limited data on these cases and most of them occurred in rural Cameroon. These side effects are believed to be associated with elevated levels of *Loa loa* microfilariae (Mf). Microfilariae death in CNS tissue was also observed. [844]<br>Based on molecular studies, ivermectin use combined with the hyperinflammatory state induced by COVID-19 may cause interaction with GABA gated chlorine channels leading to neurotoxicity. [713]<br>Serious adverse neurological events can occur when Ivermectin is used to treat *Onchocerciasis volvulus.* While the mechanism of these effects was thought to be due to *Loa loa* coinfections, a review of VigiBase adverse event reports suggests that while serious adverse effects of Ivermectin are rare, they can occur in the treatment of any disease. [845]
Leronlimab (PRO 140)	**Limited Information:** Not many studies focused on the CNS exclusively. The drug reduced plasma levels of IL-6 and decreased the amount of SARS-CoV-2 virus in 10 severely ill COVID patients. There are studies currently ongoing, with no results published as of yet. These preliminary results however do indicate that leronlimab may have an anti-inflammatory effect that could be useful in combating neurological impacts of COVID. [246]
Lopinavir/ Ritonavir (LPV/RTV)	**Negative:** LPV/RTV has been shown to induce serotonin syndrome, which is a potentially life-threatening condition [846], in some HIV patients [847], and more recently the first two cases of serotonin syndrome in COVID19 patients receiving this treatment was reported. This is mainly due to coadministration of other substances that increase serotonin levels, since RTV only reduces elimination rate, which on its own is usually not enough to induce serotonin syndrome. [848]<br>LPV causes oxidative stress in neurons in vitro, triggering the endogenous antioxidant response. It also caused a 54% loss of MAP2, which is a measure of neuronal health and function. [849]
Methyl Prednisolone (MP)	**Positive:** High dose MP reduced the expression of adhesion molecules on cerebral epithelial cells, decreasing the amount of MNC migration into the CNS. [850]<br>MP is effective in treating spinal cord injury by reducing edema and protecting cells against peroxidation. [851]
Nafamostat (NM)	**Positive:** NM shows neuroprotective effects in patients with neurovascular ischemia, reducing neuronal cell death (necrosis and apoptosis) and preventing changes to the BBB. Potential effects include reducing cerebral edema, BBB disruption, and inflammatory cytokines (IL-1B, TNF-a); increasing anti-inflammatory cytokines (IL-4,IL-10,TGF-β), p53 and RELB. [725]<br>In a rodent model, NM improved functional recovery following stroke by inhibiting neuroinflammation. NM both decreased expression of proinflammatory molecules and increased expression of anti-inflammatory molecules at different timepoints throughout recovery. [66]<br>NM was found to prevent infection with Middle East coronavirus infection. Potential neurologic side effects may include headaches and easy bleeding in the brain. [852]
Niclosamide	**Positive:** It reversed mechanical hyperalgesia (hypersensitivity to pain) in a rat model of neuropathic pain. [853]<br>Nasal and pulmonary delivery of niclosamide (via particles of human lysozyme as a carrier molecule) was found to reduce viral load in the brain of hACE2 transgenic mice infected intranasally with SARS-CoV-2. [854]
Nitazoxanide (NTZ)	**Positive:** In patients with hepatic encephalopathy (a neurological disorder caused by liver dysfunction), NTZ induced clinical improvement. [855]<br>In equine protozoal myeloencephalitis, NTZ is considered to be an effective treatment, supporting its ability to combat encephalopathies. [856]

	**Negative:** In a clinical trial, 4.8% of patients treated with NTZ for *B hominis* infection reported headaches. The side effect was not severe enough to discontinue treatment. [857]
Nitric Oxide (NO)	**Negative:** In cerebral ischemia, NO can contribute to neuronal death. NO reaction with O_2^- forms reactive intermediates which can then react with proteins, DNA, and other compounds within the cell. The endogenous mechanisms for preventing NO toxicity, but at high NO concentrations these mechanisms are overwhelmed and neurotoxic effects are observed. [858]
Remdesivir (RDV)	**Neutral:** In COVID-19 patients, no significant negative neurological effects were reported with RDV. [859]<br>Does not appear to have significant effects on the CNS either positive or negative. [860]
Ruxolitinib (RUX)	**Positive:** In human cell lines, RUX inhibited HIV replication in macrophages, and reduced HIV activation of monocytes and macrophages. In a mouse model with HIV encephalitis, the drug was detected within the brain and reduced astrogliosis. [861]
Sarilumab	**Positive:** When used long-term (5 years) in RA patients, sarilumab exhibited no severe neurological adverse effects. [862]<br>In RA patients who switched from adalimumab to sarilumab, improvement in symptoms, including neurologic pain, was reported with no severe adverse neurological effects. [863]
Sirolimus (Rapamycin)	**Positive:** In mice with tuberous sclerosis complex, rapamycin prevented development of epilepsy if administered early enough, and if administered after the onset of epilepsy the drug suppressed seizures and increased survival time. [864]<br>In various CNS tumors, human case studies and early clinical trials have suggested that rapamycin may help to inhibit abnormal growth through mTOR inhibition. Animal models have also suggested a correlation between rapamycin and reduced learning and behavioral deficits but did not conclusively confirm that the deficits are due to rapamycin alone, and the human subjects didn't undergo neuropsychiatric evaluation. In the human subjects, no significant neurological adverse effects were noted. [865]
Sofosbuvir	**Positive:** A study suggests that sofosbuvir protects against SARS-CoV-2 infection in human brain organoids. [866]<br><br>**Negative:** Case study shows sofosbuvir induced syncope in two HCV patients. The study suggests that this may be indicative of other autonomic nervous system disturbances by Sofosbuvir, which should be investigated further. [867]<br>Headaches and fatigue appear as common adverse effects of sofosbuvir treatments. Headaches can be severe enough to cause poor adherence to treatment plans, causing issues with effectiveness. [868]
Statins	**Positive:** Simvastatin and lovastatin reduce migration of MS derived monocytes and lymphocytes across the blood brain barrier (BBB). In vitro, the mechanism involved alteration of the isoprenylation processes of BBB epithelial cells. Specifically, the statins reduced secretion of CCL2 and CXCL10 chemokines by the human BBB cells. [869]
Stavudine	**Positive:** In a trial with HIV patients, stavudine used with lamivudine reduced viral RNA count in the cerebrospinal fluid (CSF), indicating that it is effective in combating HIV infection within the CNS. This combination was found to be equally as effective as zidovudine plus lamivudine. [870]<br><br>**Negative:** Stavudine was found to reduce N-acetylaspartate (a marker of mitochondrial function) in the frontal lobe of HIV patients. Stavudine has a mitochondrial toxicity in the brain which worsens the longer the drug is administered. [871]<br>Peripheral neuropathies were observed in 20% of HIV patients treated with stavudine. Patients who were on stavudine for longer than two years had a 30% rate of occurrence, suggesting that the likelihood of peripheral neuropathy increases with duration of treatment. [872]<br>Stavudine in combination with didanosine and efavirenz had a higher rate of occurrence of neuropathy and paresthesia than emtricitabine in the same combination. [199]

Tenofovir	**Neutral:** Considered to have low penetration of the CNS (compared to other antiretroviral drugs). [873]<br>This study further supports the low penetration of tenofovir into the CNS; tenofovir is thus unlikely to directly cause negative (or positive) effects on the CNS. From the results of three large clinical trials, the most common neuropsychiatric adverse effects were headaches and depression. [874]<br><br>**Negative:** In patients with HIV who had never previously received antiviral therapy (ART), tenofovir induced sensory neuropathy (including symptomatic and asymptomatic) in 17% of patients. [875]<br>Headache and fatigue were among the most commonly reported adverse effects associated with tenofovir treatment in chronic hepatitis B. The effects were mild and did not require discontinuation of treatment. [876]
Tetrandrine (TET)	**Positive:** In a mouse model of cerebral ischemia, TET inhibited the NLRP3 inflammasome via upregulation of Sirtuin-1. TET reduced neurological deficits and cerebral water volume, providing an overall anti- inflammatory effect. [877]<br><br>**Negative:** At high concentrations, TET can become cytotoxic. In mouse neuroblastoma cells, 10uM of TET induced apoptosis through oxidative stress. Pretreatment with antioxidants was effective in preventing TET induced apoptosis. Appropriate levels of TET in the brain must be maintained for successful treatment, as its effects on cellular redox activity can become harmful at high concentrations. [878]
Thalidomide (THD)	**Negative:** When used to treat dermatologic disorders (prurigo nodularis and aphthous stomatitis), long-term use led to peripheral neuropathy. Only short- term use is recommended due to neurotoxic effects. [879]<br>In lupus patients, THD induced neuropathies are potentially irreversible, even after discontinuing treatment. [880]<br>Neuropathies caused by THD become an issue in long-term, but short- term use hasn't been linked to these same effects. Neuropathies appeared 10 months after starting treatment in the majority of patients. [881]<br>Doses of THD greater than 150 mg per day increases the likelihood of peripheral neuropathy. 39% of all patients in the study treated with THD experienced peripheral neuropathy. [882]<br>Long-term treatment (>1 year) using THD for patients with multiple myeloma has been shown to cause neurotoxicity. [883]<br>In a case study, a woman being treated with THD for multiple myeloma developed encephalopathy as a result of fatal hepatic failure. [769]
Tocilizumab (TCZ)	**Positive:** In a case study of two patients with epilepsy, TCZ was effective in reducing seizures. The most likely mechanism proposed is by blocking binding of IL-6, a pro-inflammatory marker that may be involved in refractory epilepsy. [884]<br>TCZ inhibits the transcription of many different cytokines, including IL-1 and IL-6, and chemokines which are upregulated in amyotrophic lateral sclerosis (ALS). TCZ also decreased secretion of IL-1β, IL-6, and TNFα. Essentially, TCZ has anti-inflammatory action in the CNS. [885]<br>Has been used successfully to treat T-cell mediated CRS. [886]<br>In an observational study of patients with RA, TCZ caused a clinically significant reduction in symptoms of fatigue, pain, and depression. [887]<br><br>**Neutral:** In a clinical study of 27 COVID patients, only 3 showed neurological adverse effects, which may not necessarily be due to the drug but rather COVID itself. This was a small study without a control group. [888]<br><br>**Negative:** A case report found that TCZ contributed to an acute-onset of cerebral multifocal microangiopathy in a RA patient. [889]

Umifenovir (Arbidol)	**Positive:** Clinically severe psychiatric adverse effects were observed only in one patient (0.83%) taking umifenovir in a clinical trial on COVID-19 patients comparing umifenovir with favipiravir. Dizziness occurred in five (4.17%) patients. No other neurological effects were reported. This study did not have a placebo control group. [218]<br>Umifenovir has in vitro activity (in Vero cells) against the virus that causes tick borne encephalitis. In vivo results were not obtained. [890]
Valganciclovir/ Ganciclovir	**Positive:** Can be used to treat CNS CMV infections, as the drug can penetrate into the CNS. However, CSF concentrations only reach 15% of plasma levels, so high dosages may be required. Neurologic adverse effects are rare. [891]<br><br>**Negative:** Headache, insomnia, and peripheral neuropathy are among the most commonly reported adverse effects during maintenance therapy of CMV with Valganciclovir. These adverse effects generally weren't severe enough to discontinue treatment. [892]
Zidovudine (AZT)	**Positive:** AZT is effective in reducing viral RNA concentrations within the CSF of HIV patients. [870]<br>AZT has been shown to help reduce neurological symptoms of HIV/AIDS patients. It has improved outcomes for children with progressive encephalopathy caused by HIV, as well as reducing HIV-related changes to brain tissue as observed at autopsy. [893]<br><br>**Negative:** Adverse neurological effects of AZT are rare but can be serious, including seizures, dose-reduction encephalopathy, and myopathy. The likelihood of these adverse effects occurring increases the longer the drug is administered. [354]

References

1. Yang, W., Fan, Q. H., & Li, W. *A Fully Transparent, Flexible μECoG Array Based on Highly Conductive and Anti-reflective PEDOT: PSS-ITO-Ag-ITO Thin Films*. in *15th IEEE International Conference on Nano/Micro Engineered & Molecular Systems (IEEE-NEMS 2020)*. 2020. IEEE.
2. Almeida, C.A., et al., *Cytokine profiling in abacavir hypersensitivity patients.* Antivir Ther, 2008. **13**(2): p. 281-8.
3. Niu, X., et al., *Regulatory immune responses induced by IL-1 receptor antagonist in rheumatoid arthritis.* Molecular Immunology, 2011. **49**(1): p. 290-296.
4. Clark, K.E.N., et al., *Safety of intravenous anakinra in COVID-19 with evidence of hyperinflammation, a case series.* Rheumatology Advances in Practice, 2020. **4**(2).
5. Aouba, A., et al., *Targeting the inflammatory cascade with anakinra in moderate to severe COVID-19 pneumonia: case series.* Annals of the Rheumatic Diseases, 2020. **79**(10): p. 1381.
6. Khan, A., et al., *A pilot clinical trial of recombinant human angiotensin-converting enzyme 2 in acute respiratory distress syndrome.* Critical Care, 2017. **21**(1): p. 234.
7. Angelidi, A.M., M.J. Belanger, and C.S. Mantzoros, *Commentary: COVID-19 and diabetes mellitus: What we know, how our patients should be treated now, and what should happen next.* Metabolism: clinical and experimental, 2020. **107**: p. 154245-154245.
8. Monteil, V., et al., *Inhibition of SARS-CoV-2 Infections in Engineered Human Tissues Using Clinical-Grade Soluble Human ACE2.* Cell, 2020. **181**(4): p. 905-913.e7.
9. Zoufaly, A., et al., *Human recombinant soluble ACE2 in severe COVID-19.* The Lancet Respiratory Medicine, 2020. **8**(11): p. 1154-1158.
10. Carr, A.C. and S. Maggini, *Vitamin C and Immune Function.* Nutrients, 2017. **9**(11): p. 1211.
11. Härtel, C., et al., *Effects of vitamin C on intracytoplasmic cytokine production in human whole blood monocytes and lymphocytes.* Cytokine, 2004. **27**(4): p. 101-106.
12. Kanoh, S. and B.K. Rubin, *Mechanisms of Action and Clinical Application of Macrolides as Immunomodulatory Medications.* Clinical Microbiology Reviews, 2010. **23**(3): p. 590.
13. Shinkai, M., M.O. Henke, and B.K. Rubin, *Macrolide antibiotics as immunomodulatory medications: Proposed mechanisms of action.* Pharmacology & Therapeutics, 2008. **117**(3): p. 393-405.
14. Kikuchi, T. and A. Watanabe, *Baloxavir heralds a new era in influenza virus biology.* Respiratory Investigation, 2019. **57**(1): p. 1-2.
15. Ison, M.G., et al., *Early treatment with baloxavir marboxil in high-risk adolescent and adult outpatients with uncomplicated influenza (CAPSTONE-2): a randomised, placebo-controlled, phase 3 trial.* The Lancet Infectious Diseases, 2020. **20**(10): p. 1204-1214.
16. Kubo, S., et al., *Janus Kinase Inhibitor Baricitinib Modulates Human Innate and Adaptive Immune System.* Frontiers in immunology, 2018. **9**: p. 1510-1510.
17. Bronte, V., et al., *Baricitinib restrains the immune dysregulation in patients with severe COVID-19.* J Clin Invest, 2020. **130**(12): p. 6409-6416.
18. Praveen, D., R.C. Puvvada, and V.A. M, *Janus kinase inhibitor baricitinib is not an ideal option for management of COVID-19.* International Journal of Antimicrobial Agents, 2020. **55**(5): p. 105967.
19. Dropulic, L.K. and J.I. Cohen, *Update on New Antivirals Under Development for the Treatment of Double-Stranded DNA Virus Infections.* Clinical Pharmacology & Therapeutics, 2010. **88**(5): p. 610-619.
20. Toth, K., et al., *Hexadecyloxypropyl-cidofovir, CMX001, prevents adenovirus-induced mortality in a permissive, immunosuppressed animal model.* Proc Natl Acad Sci U S A, 2008. **105**(20): p. 7293-7.
21. Yamaya, M., et al., *The serine protease inhibitor camostat inhibits influenza virus replication and cytokine production in primary cultures of human tracheal epithelial cells.* Pulmonary Pharmacology & Therapeutics, 2015. **33**: p. 66-74.
22. van den Borne, B.E., et al., *Chloroquine and hydroxychloroquine equally affect tumor necrosis factor-alpha, interleukin 6, and interferon-gamma production by peripheral blood mononuclear cells.* J Rheumatol, 1997. **24**(1): p. 55-60.

23. Schrezenmeier, E. and T. Dörner, *Mechanisms of action of hydroxychloroquine and chloroquine: implications for rheumatology.* Nature Reviews Rheumatology, 2020. **16**(3): p. 155-166.
24. Leung, Y.Y., L.L. Yao Hui, and V.B. Kraus, *Colchicine--Update on mechanisms of action and therapeutic uses.* Seminars in arthritis and rheumatism, 2015. **45**(3): p. 341-350.
25. Farhad, S., P. Farhad, and A. Sobhan, *The Impact of Colchicine on The COVID-19 Patients; A Clinical Trial Study.* Research Square, 2020.
26. Deftereos, S.G., et al., *Effect of Colchicine vs Standard Care on Cardiac and Inflammatory Biomarkers and Clinical Outcomes in Patients Hospitalized With Coronavirus Disease 2019: The GRECCO-19 Randomized Clinical Trial.* JAMA Network Open, 2020. **3**(6): p. e2013136-e2013136.
27. Montón, C., et al., *Role of glucocorticoids on inflammatory response in nonimmunosuppressed patients with pneumonia: a pilot study.* Eur Respir J, 1999. **14**(1): p. 218-20.
28. Franchimont, D., et al., *Inhibition of Th1 Immune Response by Glucocorticoids: Dexamethasone Selectively Inhibits IL-12-Induced Stat4 Phosphorylation in T Lymphocytes.* The Journal of Immunology, 2000. **164**(4): p. 1768.
29. Villar, J., et al., *Efficacy of dexamethasone treatment for patients with the acute respiratory distress syndrome caused by COVID-19: study protocol for a randomized controlled superiority trial.* Trials, 2020. **21**(1): p. 717.
30. Franchi, S., et al., *The Effects of Alcoholism Pharmacotherapy on Immune Responses in Alcohol-Dependent Patients.* International Journal of Immunopathology and Pharmacology, 2010. **23**(3): p. 847-855.
31. Hu, J.J., et al., *FDA-approved disulfiram inhibits pyroptosis by blocking gasdermin D pore formation.* Nature Immunology, 2020. **21**(7): p. 736-745.
32. Klein, R.S. and J.B. Rubin, *Immune and nervous system CXCL12 and CXCR4: parallel roles in patterning and plasticity.* Trends Immunol, 2004. **25**(6): p. 306-14.
33. Zhou, W., et al., *Targeting CXCL12/CXCR4 Axis in Tumor Immunotherapy.* Current medicinal chemistry, 2019. **26**(17): p. 3026-3041.
34. Rao, N.V., et al., *Low anticoagulant heparin targets multiple sites of inflammation, suppresses heparin-induced thrombocytopenia, and inhibits interaction of RAGE with its ligands.* Am J Physiol Cell Physiol, 2010. **299**(1): p. C97-110.
35. Lakshmi, T.S.R., et al., *Efficacy of desulfated heparin mitigating inflammation in rat burn wound model.* Journal of Biomedical Materials Research Part B: Applied Biomaterials, 2011. **97B**(2): p. 215-223.
36. Shi, C., et al., *The Potential of Low Molecular Weight Heparin to Mitigate Cytokine Storm in Severe COVID-19 Patients: A Retrospective Cohort Study.* Clinical and Translational Science, 2020. **13**(6): p. 1087-1095.
37. Castillo-Mancilla, J.R., et al., *Reduced immune activation during tenofovir-emtricitabine therapy in HIV-negative individuals.* Journal of acquired immune deficiency syndromes (1999), 2015. **68**(5): p. 495-501.
38. Masho, S.W., C.-L. Wang, and D.E. Nixon, *Review of tenofovir-emtricitabine.* Therapeutics and clinical risk management, 2007. **3**(6): p. 1097-1104.
39. Meng, J.-P., et al., *Entecavir downregulates interleukin-37 in patients with chronic active hepatitis B infection.* Journal of International Medical Research, 2020. **48**(1): p. 0300060519884157.
40. Yan, H., X. Zhang, and Y. Lv, *The Effect of Entecavir Therapy on Immune Status in Chronic Hepatitis B Patients.* Iran J Immunol, 2019. **16**(1): p. 84-91.
41. Kayaaslan, B. and R. Guner, *Adverse effects of oral antiviral therapy in chronic hepatitis B.* World journal of hepatology, 2017. **9**(5): p. 227-241.
42. Chaisson, N.F. and P.M. Hassoun, *Systemic sclerosis-associated pulmonary arterial hypertension.* Chest, 2013. **144**(4): p. 1346-1356.
43. Hata, A.N. and R.M. Breyer, *Pharmacology and signaling of prostaglandin receptors: Multiple roles in inflammation and immune modulation.* Pharmacology & Therapeutics, 2004. **103**(2): p. 147-166.
44. Mather, J.F., R.L. Seip, and R.G. McKay, *Impact of Famotidine Use on Clinical Outcomes of Hospitalized Patients With COVID-19.* Am J Gastroenterol, 2020. **115**(10): p. 1617-1623.

45. Hahm, K.B., et al., *Comparison of immunomodulative effects of the histamine-2 receptor antagonists cimetidine, ranitidine, and famotidine on peripheral blood mononuclear cells in gastric cancer patients.* Scand J Gastroenterol, 1995. **30**(3): p. 265-71.
46. Furuta, Y., et al., *Favipiravir (T-705), a novel viral RNA polymerase inhibitor.* Antiviral Research, 2013. **100**(2): p. 446-454.
47. Guedj, J., et al., *Antiviral efficacy of favipiravir against Ebola virus: A translational study in cynomolgus macaques.* PLoS Med, 2018. **15**(3): p. e1002535.
48. Oestereich, L., et al., *Successful treatment of advanced Ebola virus infection with T-705 (favipiravir) in a small animal model.* Antiviral Research, 2014. **105**: p. 17-21.
49. Yamamura, H., et al., *Effect of favipiravir and an anti-inflammatory strategy for COVID-19.* Crit Care, 2020. **24**(1): p. 413.
50. Atallah, B., et al., *Traveling for heart transplantation and returning with COVID-19: a logistical, clinical, and pharmacotherapeutic challenge from the Middle East.* Drugs Ther Perspect, 2020: p. 1-6.
51. Verbon, A., et al., *IC14, an Anti-CD14 Antibody, Inhibits Endotoxin-Mediated Symptoms and Inflammatory Responses in Humans.* The Journal of Immunology, 2001. **166**(5): p. 3599.
52. Axtelle, T. and J. Pribble, *An overview of clinical studies in healthy subjects and patients with severe sepsis with IC14, a CD14-specific chimeric monoclonal antibody.* Journal of Endotoxin Research, 2003. **9**(6): p. 385-389.
53. Lampson, B.L., et al., *Idelalisib given front-line for treatment of chronic lymphocytic leukemia causes frequent immune-mediated hepatotoxicity.* Blood, 2016. **128**(2): p. 195-203.
54. Martinelli, S., et al., *Idelalisib impairs T-cell-mediated immunity in chronic lymphocytic leukemia.* Haematologica, 2018. **103**(12): p. e598-e601.
55. Price, G.E., A. Gaszewska-Mastarlarz, and D. Moskophidis, *The role of alpha/beta and gamma interferons in development of immunity to influenza A virus in mice.* J Virol, 2000. **74**(9): p. 3996-4003.
56. Gutterman, J.U., *Cytokine therapeutics: lessons from interferon alpha.* Proceedings of the National Academy of Sciences of the United States of America, 1994. **91**(4): p. 1198-1205.
57. Zhou, Q., et al., *Interferon-α2b Treatment for COVID-19.* Front Immunol, 2020. **11**: p. 1061.
58. Taylor, J.L. and S.E. Grossberg, *The effects of interferon-alpha on the production and action of other cytokines.* Semin Oncol, 1998. **25**(1 Suppl 1): p. 23-9.
59. Sajid, M.S., et al., *Effect of ivermectin on the cellular and humoral immune responses of rabbits.* Life Sciences, 2007. **80**(21): p. 1966-1970.
60. Blakley, B.R. and C.G. Rousseaux, *Effect of ivermectin on the immune response in mice.* Am J Vet Res, 1991. **52**(4): p. 593-5.
61. Caly, L., et al., *The FDA-approved drug ivermectin inhibits the replication of SARS-CoV-2 in vitro.* Antiviral Research, 2020. **178**: p. 104787.
62. Patterson, B.K., et al., *Disruption of the CCL5/RANTES-CCR5 Pathway Restores Immune Homeostasis and Reduces Plasma Viral Load in Critical COVID-19.* medRxiv, 2020: p. 2020.05.02.20084673.
63. Chen, L., et al., *HIV protease inhibitor lopinavir-induced TNF-α and IL-6 expression is coupled to the unfolded protein response and ERK signaling pathways in macrophages.* Biochemical Pharmacology, 2009. **78**(1): p. 70-77.
64. Fan, P.T., et al., *Effect of corticosteroids on the human immune response: comparison of one and three daily 1 gm intravenous pulses of methylprednisolone.* J Lab Clin Med, 1978. **91**(4): p. 625-34.
65. So, C., et al., *High-dose, short-term corticosteroids for ARDS caused by COVID-19: a case series.* Respirol Case Rep, 2020. **8**(6): p. e00596.
66. Li, C., et al., *Nafamostat mesilate improves function recovery after stroke by inhibiting neuroinflammation in rats.* Brain Behav Immun, 2016. **56**: p. 230-45.
67. Schwertz, H., et al., *Serine Protease Inhibitor Nafamostat Given Before Reperfusion Reduces Inflammatory Myocardial Injury by Complement and Neutrophil Inhibition.* Journal of Cardiovascular Pharmacology, 2008. **52**(2).

68. Liang, L., et al., *Inhibitory effects of niclosamide on inflammation and migration of fibroblast-like synoviocytes from patients with rheumatoid arthritis.* Inflammation Research, 2015. **64**(3): p. 225-233.
69. Kim, K.S., et al., *Induction of interleukin-1 beta (IL-1β) is a critical component of lung inflammation during influenza A (H1N1) virus infection.* J Med Virol, 2015. **87**(7): p. 1104-12.
70. Thi Tran, U. and T. Kitami, *Niclosamide activates the NLRP3 inflammasome by intracellular acidification and mitochondrial inhibition.* Communications Biology, 2019. **2**(1): p. 2.
71. Schmitz, N., et al., *Interleukin-1 is responsible for acute lung immunopathology but increases survival of respiratory influenza virus infection.* J Virol, 2005. **79**(10): p. 6441-8.
72. Rossignol, J.-F., *Nitazoxanide, a new drug candidate for the treatment of Middle East respiratory syndrome coronavirus.* Journal of infection and public health, 2016. **9**(3): p. 227-230.
73. Clerici, M., et al., *The anti-infective Nitazoxanide shows strong immumodulating effects (155.21).* The Journal of Immunology, 2011. **186**(1 Supplement): p. 155.21.
74. Jasenosky, L.D., et al., *The FDA-Approved Oral Drug Nitazoxanide Amplifies Host Antiviral Responses and Inhibits Ebola Virus.* iScience, 2019. **19**: p. 1279-1290.
75. Bogdan, C., *Nitric oxide and the immune response.* Nature Immunology, 2001. **2**(10): p. 907-916.
76. Madelain, V., et al., *Ebola viral dynamics in nonhuman primates provides insights into virus immuno-pathogenesis and antiviral strategies.* Nature communications, 2018. **9**(1): p. 4013-4013.
77. Warren, T.K., et al., *Therapeutic efficacy of the small molecule GS-5734 against Ebola virus in rhesus monkeys.* Nature, 2016. **531**(7594): p. 381-385.
78. Sheahan, T.P., et al., *Comparative therapeutic efficacy of remdesivir and combination lopinavir, ritonavir, and interferon beta against MERS-CoV.* Nature Communications, 2020. **11**(1): p. 222.
79. Akinosoglou, K., et al., *Remdesivir and tocilizumab: Mix or match.* Journal of Medical Virology, 2020. **n/a**(n/a).
80. Kenderian, S.S., et al., *Ruxolitinib Prevents Cytokine Release Syndrome after Car T-Cell Therapy Without Impairing the Anti-Tumor Effect in a Xenograft Model.* Biology of Blood and Marrow Transplantation, 2017. **23**(3): p. S19-S20.
81. Cao, Y., et al., *Ruxolitinib in treatment of severe coronavirus disease 2019 (COVID-19): A multicenter, single-blind, randomized controlled trial.* Journal of Allergy and Clinical Immunology, 2020. **146**(1): p. 137-146.e3.
82. La Rosée, F., et al., *The Janus kinase 1/2 inhibitor ruxolitinib in COVID-19 with severe systemic hyperinflammation.* Leukemia, 2020. **34**(7): p. 1805-1815.
83. Verstovsek, S., et al., *A Double-Blind, Placebo-Controlled Trial of Ruxolitinib for Myelofibrosis.* New England Journal of Medicine, 2012. **366**(9): p. 799-807.
84. Srirangan, S. and E.H. Choy, *The role of interleukin 6 in the pathophysiology of rheumatoid arthritis.* Therapeutic advances in musculoskeletal disease, 2010. **2**(5): p. 247-256.
85. Fleischmann, R., et al., *Long-term safety of sarilumab in rheumatoid arthritis: an integrated analysis with up to 7 years' follow-up.* Rheumatology, 2020. **59**(2): p. 292-302.
86. Fleischmann, R., et al., *Sarilumab and Nonbiologic Disease-Modifying Antirheumatic Drugs in Patients With Active Rheumatoid Arthritis and Inadequate Response or Intolerance to Tumor Necrosis Factor Inhibitors.* Arthritis & Rheumatology, 2017. **69**(2): p. 277-290.
87. Burmester, G.R., et al., *Efficacy and safety of sarilumab monotherapy versus adalimumab monotherapy for the treatment of patients with active rheumatoid arthritis (MONARCH): a randomised, double-blind, parallel-group phase III trial.* Annals of the Rheumatic Diseases, 2017. **76**(5): p. 840.
88. Montesarchio, V., et al., *Outcomes and biomarker analyses among patients with COVID-19 treated with interleukin 6 (IL-6) receptor antagonist sarilumab at a single institution in Italy.* Journal for immunotherapy of cancer, 2020. **8**(2): p. e001089.
89. Huizinga, T.W.J., et al., *Sarilumab, a fully human monoclonal antibody against IL-6Rα in patients with rheumatoid arthritis and an inadequate response to methotrexate: efficacy and safety results from the randomised SARIL-RA-MOBILITY Part A trial.* Annals of the Rheumatic Diseases, 2014. **73**(9): p. 1626-1634.

90. Säemann, M.D., et al., *The Multifunctional Role of mTOR in Innate Immunity: Implications for Transplant Immunity.* American Journal of Transplantation, 2009. **9**(12): p. 2655-2661.
91. Weichhart, T., et al., *The TSC-mTOR Signaling Pathway Regulates the Innate Inflammatory Response.* Immunity, 2008. **29**(4): p. 565-577.
92. Säemann, M.D. and G. Remuzzi, *Time to rethink immunosuppression by mTOR inhibitors?* Nature Reviews Nephrology, 2009. **5**(11): p. 611-612.
93. Steinebrunner, N., et al., *Early virological response may predict treatment response in sofosbuvir-based combination therapy of chronic hepatitis c in a multi-center "real-life" cohort.* BMC Gastroenterol, 2015. **15**: p. 97.
94. Kwak, B., et al., *Statins as a newly recognized type of immunomodulator.* Nature Medicine, 2000. **6**(12): p. 1399-1402.
95. Loppnow, H., et al., *Statins potently reduce the cytokine-mediated IL-6 release in SMC/MNC cocultures.* Journal of Cellular and Molecular Medicine, 2011. **15**(4): p. 994-1004.
96. Ubels, F.L., et al., *Effects of initial and long-term lipid-lowering therapy on vascular wall characteristics.* Atherosclerosis, 2001. **154**(1): p. 155-61.
97. Novaro, G.M., et al., *Effect of hydroxymethylglutaryl coenzyme a reductase inhibitors on the progression of calcific aortic stenosis.* Circulation, 2001. **104**(18): p. 2205-9.
98. Trabattoni, D., et al., *Modulation of human immunodeficiency virus (HIV)-specific immune response by using efavirenz, nelfinavir, and stavudine in a rescue therapy regimen for HIV-infected, drug-experienced patients.* Clin Diagn Lab Immunol, 2002. **9**(5): p. 1114-8.
99. Lagathu, C., et al., *Antiretroviral drugs with adverse effects on adipocyte lipid metabolism and survival alter the expression and secretion of proinflammatory cytokines and adiponectin in vitro.* Antivir Ther, 2004. **9**(6): p. 911-20.
100. Tavassoli, N., et al., *Effects of discontinuing stavudine or protease inhibitor therapy on human immunodeficiency virus-related fat redistribution evaluated by dual-energy x-ray absorptiometry.* Pharmacotherapy, 2006. **26**(2): p. 154-61.
101. Biswas, N., et al., *Effects of tenofovir on cytokines and nucleotidases in HIV-1 target cells and the mucosal tissue environment in the female reproductive tract.* Antimicrobial agents and chemotherapy, 2014. **58**(11): p. 6444-6453.
102. Melchjorsen, J., et al., *Tenofovir selectively regulates production of inflammatory cytokines and shifts the IL-12/IL-10 balance in human primary cells.* J Acquir Immune Defic Syndr, 2011. **57**(4): p. 265-75.
103. Zou, H., T. He, and X. Chen, *Tetrandrine inhibits differentiation of proinflammatory subsets of T helper cells but spares de novo differentiation of iTreg cells.* International Immunopharmacology, 2019. **69**: p. 307-312.
104. Steinman, L., *A brief history of T(H)17, the first major revision in the T(H)1/T(H)2 hypothesis of T cell-mediated tissue damage.* Nat Med, 2007. **13**(2): p. 139-45.
105. Afzali, B., et al., *The role of T helper 17 (Th17) and regulatory T cells (Treg) in human organ transplantation and autoimmune disease.* Clin Exp Immunol, 2007. **148**(1): p. 32-46.
106. Kim, D.E., et al., *Natural Bis-Benzylisoquinoline Alkaloids-Tetrandrine, Fangchinoline, and Cepharanthine, Inhibit Human Coronavirus OC43 Infection of MRC-5 Human Lung Cells.* Biomolecules, 2019. **9**(11): p. 696.
107. Wu, D. and X.O. Yang, *TH17 responses in cytokine storm of COVID-19: An emerging target of JAK2 inhibitor Fedratinib.* Journal of Microbiology, Immunology and Infection, 2020. **53**(3): p. 368-370.
108. Huang, C., et al., *Clinical features of patients infected with 2019 novel coronavirus in Wuhan, China.* The Lancet, 2020. **395**(10223): p. 497-506.
109. Quach, H., et al., *Mechanism of action of immunomodulatory drugs (IMiDS) in multiple myeloma.* Leukemia, 2010. **24**(1): p. 22-32.
110. Sampaio, E.P., et al., *Thalidomide selectively inhibits tumor necrosis factor alpha production by stimulated human monocytes.* J Exp Med, 1991. **173**(3): p. 699-703.
111. Amato, R.J., et al., *Thalidomide is an inhibitor of angiogenesis.* Proceedings of the National Academy of Sciences, 1994. **91**(9): p. 4082.

112. Zhou, S., et al., *Thalidomide-a notorious sedative to a wonder anticancer drug.* Current medicinal chemistry, 2013. **20**(33): p. 4102-4108.
113. Sampaio, E.P., et al., *The Influence of Thalidomide on the Clinical and Immunologic Manifestation of Erythema Nodosum Leprosum.* The Journal of Infectious Diseases, 1993. **168**(2): p. 408-414.
114. Zorat, F., et al., *The clinical and biological effects of thalidomide in patients with myelodysplastic syndromes.* British Journal of Haematology, 2001. **115**(4): p. 881-894.
115. Gullestad, L., et al., *Effect of Thalidomide on Cardiac Remodeling in Chronic Heart Failure.* Circulation, 2005. **112**(22): p. 3408-3414.
116. Lee, D.W., et al., *Current concepts in the diagnosis and management of cytokine release syndrome.* Blood, 2014. **124**(2): p. 188-195.
117. Reagan, J.L., et al., *Cellular immunotherapy for refractory hematological malignancies.* J Transl Med, 2013. **11**: p. 150.
118. Grupp, S.A., et al., *Chimeric antigen receptor-modified T cells for acute lymphoid leukemia.* N Engl J Med, 2013. **368**(16): p. 1509-1518.
119. Mihara, M., et al., *Tocilizumab inhibits signal transduction mediated by both mIL-6R and sIL-6R, but not by the receptors of other members of IL-6 cytokine family.* Int Immunopharmacol, 2005. **5**(12): p. 1731-40.
120. Kaye, A.G. and R. Siegel, *The efficacy of IL-6 inhibitor Tocilizumab in reducing severe COVID-19 mortality: a systematic review.* PeerJ, 2020. **8**: p. e10322.
121. Qian, S., et al., *Tocilizumab exerts anti-inflammatory activity in six critically ill COVID-19 patients: a retrospective analysis.* Ann Transl Med, 2020. **8**(14): p. 881.
122. Biran, N., et al., *Tocilizumab among patients with COVID-19 in the intensive care unit: a multicentre observational study.* Lancet Rheumatol, 2020. **2**(10): p. e603-e612.
123. Guillen, L., et al., *Preemptive interleukin-6 blockade in patients with COVID-19.* Sci Rep, 2020. **10**(1): p. 16826.
124. Price, C.C., et al., *Tocilizumab Treatment for Cytokine Release Syndrome in Hospitalized Patients With Coronavirus Disease 2019: Survival and Clinical Outcomes.* Chest, 2020. **158**(4): p. 1397-1408.
125. Scheller, J., et al., *The pro- and anti-inflammatory properties of the cytokine interleukin-6.* Biochim Biophys Acta, 2011. **1813**(5): p. 878-88.
126. Morel, J., et al., *Risk factors of serious infections in patients with rheumatoid arthritis treated with tocilizumab in the French Registry REGATE.* Rheumatology (Oxford), 2017. **56**(10): p. 1746-1754.
127. Schiff, M.H., et al., *Integrated safety in tocilizumab clinical trials.* Arthritis Res Ther, 2011. **13**(5): p. R141.
128. Campbell, L., et al., *Risk of adverse events including serious infections in rheumatoid arthritis patients treated with tocilizumab: a systematic literature review and meta-analysis of randomized controlled trials.* Rheumatology (Oxford), 2011. **50**(3): p. 552-62.
129. Deng, H.-Y., et al., *Efficacy of arbidol on lethal hantaan virus infections in suckling mice and in vitro.* Acta pharmacologica Sinica, 2009. **30**(7): p. 1015-1024.
130. Liu, Q., et al., *Antiviral and anti-inflammatory activity of arbidol hydrochloride in influenza A (H1N1) virus infection.* Acta pharmacologica Sinica, 2013. **34**(8): p. 1075-1083.
131. Glushkov, R.G., et al., *[Mechanisms of arbidole's immunomodulating action].* Vestn Ross Akad Med Nauk, 1999(3): p. 36-40.
132. Lian, N., et al., *Umifenovir treatment is not associated with improved outcomes in patients with coronavirus disease 2019: a retrospective study.* Clinical microbiology and infection : the official publication of the European Society of Clinical Microbiology and Infectious Diseases, 2020. **26**(7): p. 917-921.
133. Huang, D., et al., *Efficacy and safety of umifenovir for coronavirus disease 2019 (COVID-19): A systematic review and meta-analysis.* Journal of medical virology, 2020: p. 10.1002/jmv.26256.
134. Noble, S. and D. Faulds, *Ganciclovir. An update of its use in the prevention of cytomegalovirus infection and disease in transplant recipients.* Drugs, 1998. **56**(1): p. 115-46.
135. Curran, M. and S. Noble, *Valganciclovir.* Drugs, 2001. **61**(8): p. 1145-1150.

136. Dalakas, M.C., et al., *Mitochondrial Myopathy Caused by Long-Term Zidovudine Therapy.* New England Journal of Medicine, 1990. **322**(16): p. 1098-1105.
137. Fischl, M.A., et al., *The Efficacy of Azidothymidine (AZT) in the Treatment of Patients with AIDS and AIDS-Related Complex.* New England Journal of Medicine, 1987. **317**(4): p. 185-191.
138. Team, D.T., *Twenty-four-week safety and tolerability of nevirapine vs. abacavir in combination with zidovudine/lamivudine as first-line antiretroviral therapy: a randomized double-blind trial (NORA).* Trop Med Int Health, 2008. **13**(1): p. 6-16.
139. Del Amo, J., et al., *Incidence and Severity of COVID-19 in HIV-Positive Persons Receiving Antiretroviral Therapy : A Cohort Study.* Ann Intern Med, 2020. **173**(7): p. 536-541.
140. Keiser, P., et al., *Comparison of symptoms of influenza A with abacavir-associated hypersensitivity reaction.* Int J STD AIDS, 2003. **14**(7): p. 478-81.
141. Mertens, M. and J.A. Singh, *Anakinra for rheumatoid arthritis: a systematic review.* J Rheumatol, 2009. **36**(6): p. 1118-25.
142. Cavalli, G., et al., *Interleukin-1 blockade with high-dose anakinra in patients with COVID-19, acute respiratory distress syndrome, and hyperinflammation: a retrospective cohort study.* Lancet Rheumatol, 2020. **2**(6): p. e325-e331.
143. Huet, T., et al., *Anakinra for severe forms of COVID-19: a cohort study.* Lancet Rheumatol, 2020. **2**(7): p. e393-e400.
144. Iglesias-Julián, E., et al., *High dose subcutaneous Anakinra to treat acute respiratory distress syndrome secondary to cytokine storm syndrome among severely ill COVID-19 patients.* J Autoimmun, 2020. **115**: p. 102537.
145. Zoufaly, A., et al., *Human recombinant soluble ACE2 in severe COVID-19.* Lancet Respir Med, 2020. **8**(11): p. 1154-1158.
146. Hemilä, H. and P. Louhiala, *Vitamin C may affect lung infections.* J R Soc Med, 2007. **100**(11): p. 495-8.
147. Rodrigues da Silva, M., et al., *Beneficial effects of ascorbic acid to treat lung fibrosis induced by paraquat.* PLoS One, 2018. **13**(11): p. e0205535.
148. Hemilä, H. and E. Chalker, *Vitamin C Can Shorten the Length of Stay in the ICU: A Meta-Analysis.* Nutrients, 2019. **11**(4).
149. Cai, Y., et al., *Effectiveness and safety of macrolides in cystic fibrosis patients: a meta-analysis and systematic review.* J Antimicrob Chemother, 2011. **66**(5): p. 968-78.
150. Albert, R.K., et al., *Azithromycin for prevention of exacerbations of COPD.* N Engl J Med, 2011. **365**(8): p. 689-98.
151. Shimizu, T. and S. Shimizu, *Azithromycin inhibits mucus hypersecretion from airway epithelial cells.* Mediators Inflamm, 2012. **2012**: p. 265714.
152. Segal, L.N., et al., *Randomised, double-blind, placebo-controlled trial with azithromycin selects for anti-inflammatory microbial metabolites in the emphysematous lung.* Thorax, 2017. **72**(1): p. 13-22.
153. Gautret, P., et al., *Hydroxychloroquine and azithromycin as a treatment of COVID-19: results of an open-label non-randomized clinical trial.* Int J Antimicrob Agents, 2020. **56**(1): p. 105949.
154. Gautret, P., et al., *Clinical and microbiological effect of a combination of hydroxychloroquine and azithromycin in 80 COVID-19 patients with at least a six-day follow up: A pilot observational study.* Travel Med Infect Dis, 2020. **34**: p. 101663.
155. Furtado, R.H.M., et al., *Azithromycin in addition to standard of care versus standard of care alone in the treatment of patients admitted to the hospital with severe COVID-19 in Brazil (COALITION II): a randomised clinical trial.* Lancet, 2020. **396**(10256): p. 959-967.
156. Hayden, F.G., et al., *Baloxavir Marboxil for Uncomplicated Influenza in Adults and Adolescents.* N Engl J Med, 2018. **379**(10): p. 913-923.
157. Kikuchi, T. and A. Watanabe, *Baloxavir heralds a new era in influenza virus biology.* Respir Investig, 2019. **57**(1): p. 1-2.
158. Kiso, M., et al., *Treatment of Highly Pathogenic H7N9 Virus-Infected Mice with Baloxavir Marboxil.* Viruses, 2019. **11**(11).

159. Fukao, K., et al., *Combination treatment with the cap-dependent endonuclease inhibitor baloxavir marboxil and a neuraminidase inhibitor in a mouse model of influenza A virus infection.* J Antimicrob Chemother, 2019. **74**(3): p. 654-662.
160. Wang, X., et al., *The anti-influenza virus drug, arbidol is an efficient inhibitor of SARS-CoV-2 in vitro.* Cell Discov, 2020. **6**: p. 28.
161. Lou, Y., et al., *Clinical Outcomes and Plasma Concentrations of Baloxavir Marboxil and Favipiravir in COVID-19 Patients: An Exploratory Randomized, Controlled Trial.* Eur J Pharm Sci, 2020: p. 105631.
162. Uehara, T., et al., *Treatment-Emergent Influenza Variant Viruses With Reduced Baloxavir Susceptibility: Impact on Clinical and Virologic Outcomes in Uncomplicated Influenza.* J Infect Dis, 2020. **221**(3): p. 346-355.
163. Taylor, P.C., et al., *Cardiovascular Safety During Treatment With Baricitinib in Rheumatoid Arthritis.* Arthritis Rheumatol, 2019. **71**(7): p. 1042-1055.
164. Lo Caputo, S., et al., *Baricitinib: A chance to treat COVID-19?* J Med Virol, 2020.
165. Kalil, A.C., et al., *Baricitinib plus Remdesivir for Hospitalized Adults with Covid-19.* N Engl J Med, 2020.
166. Sudhindra, P., et al., *Brincidofovir (CMX001) for the Treatment of Severe Adenoviral Pneumonia in Kidney Transplant Recipient.* Cureus, 2019. **11**(8): p. e5296.
167. Van Genechten, T., et al., *Successful Treatment of Adenovirus Infection with Brincidofovir in an Immunocompromised Patient after Hematological Stem Cell Transplantation.* Case Rep Infect Dis, 2020. **2020**: p. 5981289.
168. Liekens, S., et al., *The broad-spectrum anti-DNA virus agent cidofovir inhibits lung metastasis of virus-independent, FGF2-driven tumors.* Oncotarget, 2015. **6**(7): p. 4633-48.
169. Kitagawa, M., et al., *Pharmaceutical development for treating pancreatic diseases.* Pancreas, 1998. **16**(3): p. 427-31.
170. Bardou, O., et al., *Membrane-anchored Serine Protease Matriptase Is a Trigger of Pulmonary Fibrogenesis.* Am J Respir Crit Care Med, 2016. **193**(8): p. 847-60.
171. Zhou, Y., et al., *Protease inhibitors targeting coronavirus and filovirus entry.* Antiviral Res, 2015. **116**: p. 76-84.
172. Shang, J., et al., *Cell entry mechanisms of SARS-CoV-2.* Proc Natl Acad Sci U S A, 2020. **117**(21): p. 11727-11734.
173. Hoffmann, M., et al., *SARS-CoV-2 Cell Entry Depends on ACE2 and TMPRSS2 and Is Blocked by a Clinically Proven Protease Inhibitor.* Cell, 2020. **181**(2): p. 271-280.e8.
174. Ota, S., et al., *Acute eosinophilic pneumonia caused by camostat mesilate: The first case report.* Respir Med Case Rep, 2016. **19**: p. 21-3.
175. Vincent, M.J., et al., *Chloroquine is a potent inhibitor of SARS coronavirus infection and spread.* Virol J, 2005. **2**: p. 69.
176. Wang, N., et al., *Chloroquine and hydroxychloroquine as ACE2 blockers to inhibit viropexis of 2019-nCoV Spike pseudotyped virus.* Phytomedicine, 2020. **79**: p. 153333.
177. Hoffmann, M., et al., *Chloroquine does not inhibit infection of human lung cells with SARS-CoV-2.* Nature, 2020. **585**(7826): p. 588-590.
178. Kashour, Z., et al., *Efficacy of chloroquine or hydroxychloroquine in COVID-19 patients: a systematic review and meta-analysis.* J Antimicrob Chemother, 2020.
179. Addrizzo-Harris, D.J., et al., *Mechanisms of colchicine effect in the treatment of asbestosis and idiopathic pulmonary fibrosis.* Lung, 2002. **180**(2): p. 61-72.
180. Lopes, M.I.F., et al., *Beneficial effects of colchicine for moderate to severe COVID-19: an interim analysis of a randomized, double-blinded, placebo controlled clinical trial.* medRxiv, 2020.
181. Douglas, W.W., et al., *Colchicine versus prednisone in the treatment of idiopathic pulmonary fibrosis. A randomized prospective study. Members of the Lung Study Group.* Am J Respir Crit Care Med, 1998. **158**(1): p. 220-5.
182. Falk, J.A., O.A. Minai, and Z. Mosenifar, *Inhaled and systemic corticosteroids in chronic obstructive pulmonary disease.* Proc Am Thorac Soc, 2008. **5**(4): p. 506-12.
183. Stern, A., et al., *Corticosteroids for pneumonia.* Cochrane Database Syst Rev, 2017. **12**: p. CD007720.

184. Majmundar, M., et al., *Efficacy of corticosteroids in non-intensive care unit patients with COVID-19 pneumonia from the New York Metropolitan region.* PLoS One, 2020. **15**(9): p. e0238827.
185. Sterne, J.A.C., et al., *Association Between Administration of Systemic Corticosteroids and Mortality Among Critically Ill Patients With COVID-19: A Meta-analysis.* JAMA, 2020. **324**(13): p. 1330-1341.
186. Ernst, P., N. Saad, and S. Suissa, *Inhaled corticosteroids in COPD: the clinical evidence.* Eur Respir J, 2015. **45**(2): p. 525-37.
187. Mokra, D., et al., *Corticosteroids in Acute Lung Injury: The Dilemma Continues.* Int J Mol Sci, 2019. **20**(19).
188. Singh, A.K., et al., *Role of corticosteroid in the management of COVID-19: A systemic review and a Clinician's perspective.* Diabetes Metab Syndr, 2020. **14**(5): p. 971-978.
189. Russell, C., J. Millar, and J. Baillie, *Clinical evidence does not support corticosteroid treatment for 2019-nCoV lung injury.* The Lancet, 2020. **395**(10223): p. 473-475.
190. Yoder, M.C., R. Chua, and R. Tepper, *Effect of dexamethasone on pulmonary inflammation and pulmonary function of ventilator-dependent infants with bronchopulmonary dysplasia.* Am Rev Respir Dis, 1991. **143**(5 Pt 1): p. 1044-8.
191. Villar, J., et al., *Dexamethasone treatment for the acute respiratory distress syndrome: a multicentre, randomised controlled trial.* Lancet Respir Med, 2020. **8**(3): p. 267-276.
192. Horby, P., et al., *Dexamethasone in Hospitalized Patients with Covid-19 - Preliminary Report.* N Engl J Med, 2020.
193. Tomazini, B.M., et al., *Effect of Dexamethasone on Days Alive and Ventilator-Free in Patients With Moderate or Severe Acute Respiratory Distress Syndrome and COVID-19: The CoDEX Randomized Clinical Trial.* JAMA, 2020. **324**(13): p. 1307-1316.
194. Kim, J.Y., et al., *Disulfiram induces anoikis and suppresses lung colonization in triple-negative breast cancer via calpain activation.* Cancer Lett, 2017. **386**: p. 151-160.
195. Nechushtan, H., et al., *A phase IIb trial assessing the addition of disulfiram to chemotherapy for the treatment of metastatic non-small cell lung cancer.* Oncologist, 2015. **20**(4): p. 366-7.
196. Lin, M.H., et al., *Disulfiram can inhibit MERS and SARS coronavirus papain-like proteases via different modes.* Antiviral Res, 2018. **150**: p. 155-163.
197. Griffin, K.L., et al., *2-O, 3-O-desulfated heparin inhibits neutrophil elastase-induced HMGB-1 secretion and airway inflammation.* Am J Respir Cell Mol Biol, 2014. **50**(4): p. 684-9.
198. Sharma, L., et al., *Partially-desulfated heparin improves survival in Pseudomonas pneumonia by enhancing bacterial clearance and ameliorating lung injury.* J Immunotoxicol, 2014. **11**(3): p. 260-7.
199. Saag, M.S., et al., *Efficacy and safety of emtricitabine vs stavudine in combination therapy in antiretroviral-naive patients: a randomized trial.* JAMA, 2004. **292**(2): p. 180-9.
200. Park, S.J., et al., *Antiviral Efficacies of FDA-Approved Drugs against SARS-CoV-2 Infection in Ferrets.* mBio, 2020. **11**(3).
201. Auld, S.C., et al., *Declines in Lung Function After Antiretroviral Therapy Initiation in Adults With Human Immunodeficiency Virus and Tuberculosis: A Potential Manifestation of Respiratory Immune Reconstitution Inflammatory Syndrome.* Clin Infect Dis, 2020. **70**(8): p. 1750-1753.
202. Su, L., et al., *Drug repurposing of anti-infective clinical drugs: Discovery of two potential anti-cytokine storm agents.* Biomed Pharmacother, 2020. **131**: p. 110643.
203. Nemoto, M., et al., *Hepatitis B Virus-associated Vasculitis: Multiple Cavitary Masses in the Lung Mimicking Granulomatous Polyangiitis.* Intern Med, 2019. **58**(20): p. 3013-3017.
204. Kayaaslan, B. and R. Guner, *Adverse effects of oral antiviral therapy in chronic hepatitis B.* World J Hepatol, 2017. **9**(5): p. 227-241.
205. De Wet, C.J., et al., *Inhaled prostacyclin is safe, effective, and affordable in patients with pulmonary hypertension, right heart dysfunction, and refractory hypoxemia after cardiothoracic surgery.* J Thorac Cardiovasc Surg, 2004. **127**(4): p. 1058-67.
206. Saito, Y., et al., *Epoprostenol sodium for treatment of pulmonary arterial hypertension.* Vasc Health Risk Manag, 2015. **11**: p. 265-70.

207. Buckley, M.S., et al., *Comparison of Fixed-Dose Inhaled Epoprostenol and Inhaled Nitric Oxide for Acute Respiratory Distress Syndrome in Critically Ill Adults.* J Intensive Care Med, 2020: p. 885066620906800.
208. Klings, E.S., et al., *Systemic sclerosis-associated pulmonary hypertension: short- and long-term effects of epoprostenol (prostacyclin).* Arthritis Rheum, 1999. **42**(12): p. 2638-45.
209. Moezinia, C.J., et al., *Iloprost for COVID-19-related vasculopathy.* Lancet Rheumatol, 2020. **2**(10): p. e582-e583.
210. Sonti, R., C.W. Pike, and N. Cobb, *Responsiveness of Inhaled Epoprostenol in Respiratory Failure due to COVID-19.* J Intensive Care Med, 2020: p. 885066620976525.
211. DeGrado, J.R., et al., *Evaluation of the Efficacy and Safety of Inhaled Epoprostenol and Inhaled Nitric Oxide for Refractory Hypoxemia in Patients With Coronavirus Disease 2019.* Crit Care Explor, 2020. **2**(10): p. e0259.
212. Hogan Ii, R.B., et al., *Dual-histamine receptor blockade with cetirizine - famotidine reduces pulmonary symptoms in COVID-19 patients.* Pulm Pharmacol Ther, 2020. **63**: p. 101942.
213. Freedberg, D.E., et al., *Famotidine Use Is Associated With Improved Clinical Outcomes in Hospitalized COVID-19 Patients: A Propensity Score Matched Retrospective Cohort Study.* Gastroenterology, 2020. **159**(3): p. 1129-1131.e3.
214. Lee, S.W., et al., *The impact of acid-suppressing drugs to the patients with chronic obstructive pulmonary disease: A nationwide, population-based, cohort study.* J Res Med Sci, 2015. **20**(3): p. 263-7.
215. Furuta, Y., T. Komeno, and T. Nakamura, *Favipiravir (T-705), a broad spectrum inhibitor of viral RNA polymerase.* Proc Jpn Acad Ser B Phys Biol Sci, 2017. **93**(7): p. 449-463.
216. Safronetz, D., et al., *Antiviral efficacy of favipiravir against two prominent etiological agents of hantavirus pulmonary syndrome.* Antimicrob Agents Chemother, 2013. **57**(10): p. 4673-80.
217. Cai, Q., et al., *Experimental Treatment with Favipiravir for COVID-19: An Open-Label Control Study.* Engineering (Beijing), 2020.
218. Chen, C., et al., *Favipiravir versus Arbidol for COVID-19: A Randomized Clinical Trial.* medRxiv, 2020.
219. Dörner, T., *Therapy: Hydroxychloroquine in SLE: old drug, new perspectives.* Nat Rev Rheumatol, 2010. **6**(1): p. 10-1.
220. Morand, E.F., P.I. McCloud, and G.O. Littlejohn, *Continuation of long term treatment with hydroxychloroquine in systemic lupus erythematosus and rheumatoid arthritis.* Ann Rheum Dis, 1992. **51**(12): p. 1318-21.
221. Li, Y., et al., *Hydroxychloroquine induced lung cancer suppression by enhancing chemo-sensitization and promoting the transition of M2-TAMs to M1-like macrophages.* J Exp Clin Cancer Res, 2018. **37**(1): p. 259.
222. McDonnell, M., et al., *Experience of hydroxychloroquine in the treatment of pulmonary sarcoidosis in the west of Ireland: An insight into clinical practice.* European Respiratory Journal, 2011. **38**: p. 3736.
223. Yao, X., et al., *In Vitro Antiviral Activity and Projection of Optimized Dosing Design of Hydroxychloroquine for the Treatment of Severe Acute Respiratory Syndrome Coronavirus 2 (SARS-CoV-2).* Clin Infect Dis, 2020. **71**(15): p. 732-739.
224. Geleris, J., et al., *Observational Study of Hydroxychloroquine in Hospitalized Patients with Covid-19.* N Engl J Med, 2020. **382**(25): p. 2411-2418.
225. Boulware, D.R., et al., *A Randomized Trial of Hydroxychloroquine as Postexposure Prophylaxis for Covid-19.* N Engl J Med, 2020. **383**(6): p. 517-525.
226. Verbon, A., et al., *IC14, an anti-CD14 antibody, inhibits endotoxin-mediated symptoms and inflammatory responses in humans.* J Immunol, 2001. **166**(5): p. 3599-605.
227. Axtelle, T. and J. Pribble, *IC14, a CD14 specific monoclonal antibody, is a potential treatment for patients with severe sepsis.* J Endotoxin Res, 2001. **7**(4): p. 310-4.
228. Ruokonen, E., et al., *Septic shock and multiple organ failure.* Crit Care Med, 1991. **19**(9): p. 1146-51.
229. Haustraete, E., et al., *Idelalisib-related pneumonitis.* Eur Respir J, 2016. **47**(4): p. 1280-3.

230. Wang, B., et al., *Subcutaneous injection of IFN alpha-2b for COVID-19: an observational study.* BMC Infect Dis, 2020. **20**(1): p. 723.
231. Pereda, R., et al., *Therapeutic Effectiveness of Interferon-α2b Against COVID-19: The Cuban Experience.* J Interferon Cytokine Res, 2020. **40**(9): p. 438-442.
232. Goritzka, M., et al., *Alpha/beta interferon receptor signaling amplifies early proinflammatory cytokine production in the lung during respiratory syncytial virus infection.* J Virol, 2014. **88**(11): p. 6128-36.
233. Midturi, J., et al., *Spectrum of pulmonary toxicity associated with the use of interferon therapy for hepatitis C: case report and review of the literature.* Clin Infect Dis, 2004. **39**(11): p. 1724-9.
234. Anderson, P., M. Höglund, and S. Rödjer, *Pulmonary side effects of interferon-alpha therapy in patients with hematological malignancies.* Am J Hematol, 2003. **73**(1): p. 54-8.
235. Nakamura, F., et al., *A case of interstitial pneumonitis associated with natural alpha-interferon therapy for myelofibrosis.* Acta Haematol, 1997. **97**(4): p. 222-4.
236. Yufu, Y., et al., *Interstitial pneumonia caused by interferon-alpha in chronic myelogenous leukemia.* Am J Hematol, 1994. **47**(3): p. 253.
237. Pietropaoli, A., J. Modrak, and M. Utell, *Interferon-alpha therapy associated with the development of sarcoidosis.* Chest, 1999. **116**(2): p. 569-72.
238. Abdi, E.A., et al., *Pulmonary sarcoidosis following interferon therapy for advanced renal cell carcinoma.* Cancer, 1987. **59**(5): p. 896-900.
239. Melotti, A., et al., *The river blindness drug Ivermectin and related macrocyclic lactones inhibit WNT-TCF pathway responses in human cancer.* EMBO Mol Med, 2014. **6**(10): p. 1263-78.
240. Caly, L., et al., *The FDA-approved drug ivermectin inhibits the replication of SARS-CoV-2 in vitro.* Antiviral Res, 2020. **178**: p. 104787.
241. *FDA approves new HIV drug after priority review.* AIDS Patient Care STDS, 2008. **22**(2): p. 159.
242. **CytoDyn, I.** *Leronlimab (PRO 140) Combined With Carboplatin in Patients With CCR5+ mTNBC*. 2020.
243. CytoDyn, I., *Study to Evaluate the Efficacy and Safety of Leronlimab for Mild to Moderate COVID-19*. 2020, ClinicalTrials.gov.
244. CytoDyn, I., *Study to Evaluate the Efficacy and Safety of Leronlimab for Patients with Severe or Critical Coronavirus Disease 2019 (COVID-19)*. 2020, ClinicalTrials.gov.
245. Yang, B., et al., *Clinical Characteristics and Outcomes of COVID-19 Patients Receiving Compassionate Use Leronlimab.* Clin Infect Dis, 2020.
246. Patterson, B.K., et al., *Disruption of the CCL5/RANTES-CCR5 Pathway Restores Immune Homeostasis and Reduces Plasma Viral Load in Critical COVID-19.* medRxiv, 2020.
247. Kohlmeier, J.E., et al., *The chemokine receptor CCR5 plays a key role in the early memory CD8+ T cell response to respiratory virus infections.* Immunity, 2008. **29**(1): p. 101-13.
248. Amsellem, V., et al., *CCR5 as a treatment target in pulmonary arterial hypertension.* Circulation, 2014. **130**(11): p. 880-891.
249. Dawson, T.C., et al., *Contrasting effects of CCR5 and CCR2 deficiency in the pulmonary inflammatory response to influenza A virus.* Am J Pathol, 2000. **156**(6): p. 1951-9.
250. Chan, J.F., et al., *Treatment With Lopinavir/Ritonavir or Interferon-β1b Improves Outcome of MERS-CoV Infection in a Nonhuman Primate Model of Common Marmoset.* J Infect Dis, 2015. **212**(12): p. 1904-13.
251. Osborne, V., et al., *Lopinavir-Ritonavir in the Treatment of COVID-19: A Dynamic Systematic Benefit-Risk Assessment.* Drug Saf, 2020. **43**(8): p. 809-821.
252. Cao, B., et al., *A Trial of Lopinavir-Ritonavir in Adults Hospitalized with Severe Covid-19.* N Engl J Med, 2020. **382**(19): p. 1787-1799.
253. Li, Y., et al., *Efficacy and Safety of Lopinavir/Ritonavir or Arbidol in Adult Patients with Mild/Moderate COVID-19: An Exploratory Randomized Controlled Trial.* Med (N Y), 2020.
254. Tu, G.W., et al., *Glucocorticoid attenuates acute lung injury through induction of type 2 macrophage.* J Transl Med, 2017. **15**(1): p. 181.
255. Atri, C., F.Z. Guerfali, and D. Laouini, *Role of Human Macrophage Polarization in Inflammation during Infectious Diseases.* Int J Mol Sci, 2018. **19**(6).

256. Edalatifard, M., et al., *Intravenous methylprednisolone pulse as a treatment for hospitalised severe COVID-19 patients: results from a randomised controlled clinical trial.* Eur Respir J, 2020.
257. Meduri, G.U., et al., *Prolonged glucocorticoid treatment is associated with improved ARDS outcomes: analysis of individual patients' data from four randomized trials and trial-level meta-analysis of the updated literature.* Intensive Care Med, 2016. **42**(5): p. 829-840.
258. Mac Sweeney, R. and D. McAuley, *Prolonged glucocorticoid treatment in acute respiratory distress syndrome - Authors' reply.* The Lancet, 2017. **389**(10078): p. 1516-1517.
259. Jeronimo, C.M.P., et al., *Methylprednisolone as Adjunctive Therapy for Patients Hospitalized With COVID-19 (Metcovid): A Randomised, Double-Blind, Phase IIb, Placebo-Controlled Trial.* Clin Infect Dis, 2020.
260. Choe, K.H., et al., *Methylprednisolone causes matrix metalloproteinase-dependent emphysema in adult rats.* Am J Respir Crit Care Med, 2003. **167**(11): p. 1516-21.
261. Uchiba, M., et al., *Effect of nafamostat mesilate on pulmonary vascular injury induced by lipopolysaccharide in rats.* Am J Respir Crit Care Med, 1997. **155**(2): p. 711-8.
262. Sendo, T., et al., *A potent tryptase inhibitor nafamostat mesilate dramatically suppressed pulmonary dysfunction induced in rats by a radiographic contrast medium.* Br J Pharmacol, 2003. **138**(5): p. 959-67.
263. Mander, S., et al., *Nafamostat mesilate negatively regulates the metastasis of triple-negative breast cancer cells.* Arch Pharm Res, 2018. **41**(2): p. 229-242.
264. Hoffmann, M., et al., *Nafamostat Mesylate Blocks Activation of SARS-CoV-2: New Treatment Option for COVID-19.* Antimicrob Agents Chemother, 2020. **64**(6).
265. Doi, K., et al., *Nafamostat mesylate treatment in combination with favipiravir for patients critically ill with Covid-19: a case series.* Crit Care, 2020. **24**(1): p. 392.
266. Wu, C.J., et al., *Inhibition of severe acute respiratory syndrome coronavirus replication by niclosamide.* Antimicrob Agents Chemother, 2004. **48**(7): p. 2693-6.
267. Cabrita, I., et al., *Niclosamide repurposed for the treatment of inflammatory airway disease.* JCI Insight, 2019. **4**(15).
268. You, S., et al., *Disruption of STAT3 by niclosamide reverses radioresistance of human lung cancer.* Mol Cancer Ther, 2014. **13**(3): p. 606-16.
269. Lee, S.L., et al., *Niclosamide enhances ROS-mediated cell death through c-Jun activation.* Biomed Pharmacother, 2014. **68**(5): p. 619-24.
270. Brunaugh, A.D., et al., *Broad-Spectrum, Patient-Adaptable Inhaled Niclosamide-Lysozyme Particles are Efficacious Against Coronaviruses in Lethal Murine Infection Models.* bioRxiv, 2020.
271. Miner, K., et al., *Drug Repurposing: The Anthelmintics Niclosamide and Nitazoxanide Are Potent TMEM16A Antagonists That Fully Bronchodilate Airways.* Front Pharmacol, 2019. **10**: p. 51.
272. Haffizulla, J., et al., *Effect of nitazoxanide in adults and adolescents with acute uncomplicated influenza: a double-blind, randomised, placebo-controlled, phase 2b/3 trial.* Lancet Infect Dis, 2014. **14**(7): p. 609-18.
273. Son, J., et al., *Nitazoxanide and JIB-04 have broad-spectrum antiviral activity and inhibit SARS-CoV-2 replication in cell culture and coronavirus pathogenesis in a pig model.* bioRxiv, 2020.
274. Wang, M., et al., *Remdesivir and chloroquine effectively inhibit the recently emerged novel coronavirus (2019-nCoV) in vitro.* Cell Res, 2020. **30**(3): p. 269-271.
275. Meneses Calderón, J., et al., *Nitazoxanide against COVID-19 in three explorative scenarios.* J Infect Dev Ctries, 2020. **14**(9): p. 982-986.
276. Gerlach, H., et al., *Time-course and dose-response of nitric oxide inhalation for systemic oxygenation and pulmonary hypertension in patients with adult respiratory distress syndrome.* Eur J Clin Invest, 1993. **23**(8): p. 499-502.
277. Rossaint, R., et al., *Inhaled nitric oxide for the adult respiratory distress syndrome.* N Engl J Med, 1993. **328**(6): p. 399-405.
278. Akerström, S., et al., *Nitric oxide inhibits the replication cycle of severe acute respiratory syndrome coronavirus.* J Virol, 2005. **79**(3): p. 1966-9.

279. Chen, L., et al., *Inhalation of nitric oxide in the treatment of severe acute respiratory syndrome: a rescue trial in Beijing.* Clin Infect Dis, 2004. **39**(10): p. 1531-5.
280. Hedenstierna, G., et al., *Nitric oxide dosed in short bursts at high concentrations may protect against Covid 19.* Nitric Oxide, 2020. **103**: p. 1-3.
281. Longobardo, A., et al., *Inhaled nitric oxide minimally improves oxygenation in COVID-19 related acute respiratory distress syndrome.* Br J Anaesth, 2020.
282. Williamson, B.N., et al., *Clinical benefit of remdesivir in rhesus macaques infected with SARS-CoV-2.* Nature, 2020. **585**(7824): p. 273-276.
283. Frediansyah, A., et al., *Remdesivir and its antiviral activity against COVID-19: A systematic review.* Clin Epidemiol Glob Health, 2020.
284. Madelain, V., et al., *Ebola viral dynamics in nonhuman primates provides insights into virus immuno-pathogenesis and antiviral strategies.* Nat Commun, 2018. **9**(1): p. 4013.
285. Pruijssers, A.J., et al., *Remdesivir Inhibits SARS-CoV-2 in Human Lung Cells and Chimeric SARS-CoV Expressing the SARS-CoV-2 RNA Polymerase in Mice.* Cell Rep, 2020. **32**(3): p. 107940.
286. Roshanshad, A., et al., *The efficacy of remdesivir in coronavirus disease 2019 (COVID-19): a systemic review.* Iranian Journal of Microbiology, 2020. **12**(5).
287. Grein, J., et al., *Compassionate Use of Remdesivir for Patients with Severe Covid-19.* N Engl J Med, 2020. **382**(24): p. 2327-2336.
288. Beigel, J.H., et al., *Remdesivir for the Treatment of Covid-19 - Final Report.* N Engl J Med, 2020. **383**(19): p. 1813-1826.
289. Wang, Y., et al., *Remdesivir in adults with severe COVID-19: a randomised, double-blind, placebo-controlled, multicentre trial.* Lancet, 2020. **395**(10236): p. 1569-1578.
290. Tabarroki, A., et al., *Ruxolitinib leads to improvement of pulmonary hypertension in patients with myelofibrosis.* Leukemia, 2014. **28**(7): p. 1486-93.
291. Neubauer, A., et al., *Ruxolitinib for the treatment of SARS-CoV-2 induced acute respiratory distress syndrome (ARDS).* Leukemia, 2020. **34**(8): p. 2276-2278.
292. Cao, Y., et al., *Ruxolitinib in treatment of severe coronavirus disease 2019 (COVID-19): A multicenter, single-blind, randomized controlled trial.* J Allergy Clin Immunol, 2020. **146**(1): p. 137-146.e3.
293. Felgenhauer, U., et al., *Inhibition of SARS-CoV-2 by type I and type III interferons.* J Biol Chem, 2020. **295**(41): p. 13958-13964.
294. Low, A.T., et al., *Pulmonary arterial hypertension exacerbated by ruxolitinib.* Haematologica, 2015. **100**(6): p. e244-5.
295. Kerget, B., et al., *Acute respiratory distress syndrome; A rare complication caused by usage of ruxolitinib.* Respir Med Case Rep, 2017. **22**: p. 243-245.
296. Tamura, Y., et al., *Ectopic upregulation of membrane-bound IL6R drives vascular remodeling in pulmonary arterial hypertension.* J Clin Invest, 2018. **128**(5): p. 1956-1970.
297. Della-Torre, E., et al., *Interleukin-6 blockade with sarilumab in severe COVID-19 pneumonia with systemic hyperinflammation: an open-label cohort study.* Ann Rheum Dis, 2020. **79**(10): p. 1277-1285.
298. Pham, P.T., et al., *Sirolimus-associated pulmonary toxicity.* Transplantation, 2004. **77**(8): p. 1215-20.
299. Vlahakis, N.E., O.B. Rickman, and T. Morgenthaler, *Sirolimus-associated diffuse alveolar hemorrhage.* Mayo Clin Proc, 2004. **79**(4): p. 541-5.
300. Li, J., S.G. Kim, and J. Blenis, *Rapamycin: one drug, many effects.* Cell Metab, 2014. **19**(3): p. 373-9.
301. Kramer, E.L., et al., *Rapamycin decreases airway remodeling and hyperreactivity in a transgenic model of noninflammatory lung disease.* J Appl Physiol (1985), 2011. **111**(6): p. 1760-7.
302. McCormack, F.X., et al., *Efficacy and safety of sirolimus in lymphangioleiomyomatosis.* N Engl J Med, 2011. **364**(17): p. 1595-606.
303. Yates, D.H., *mTOR treatment in lymphangioleiomyomatosis: the role of everolimus.* Expert Rev Respir Med, 2016. **10**(3): p. 249-60.
304. Kennedy, B.K. and J.K. Pennypacker, *Mammalian Target of Rapamycin: A Target for (Lung) Diseases and Aging.* Ann Am Thorac Soc, 2016. **13 Suppl 5**: p. S398-S401.

305. Mitani, A., et al., *Restoration of Corticosteroid Sensitivity in Chronic Obstructive Pulmonary Disease by Inhibition of Mammalian Target of Rapamycin.* Am J Respir Crit Care Med, 2016. **193**(2): p. 143-53.
306. Wang, C.H., et al., *Adjuvant treatment with a mammalian target of rapamycin inhibitor, sirolimus, and steroids improves outcomes in patients with severe H1N1 pneumonia and acute respiratory failure.* Crit Care Med, 2014. **42**(2): p. 313-21.
307. Li, M., et al., *Efficacy and safety of mTOR inhibitors (rapamycin and its analogues) for tuberous sclerosis complex: a meta-analysis.* Orphanet J Rare Dis, 2019. **14**(1): p. 39.
308. Ahya, V.N., et al., *Increased risk of venous thromboembolism with a sirolimus-based immunosuppression regimen in lung transplantation.* J Heart Lung Transplant, 2011. **30**(2): p. 175-81.
309. Malouf, M.A., et al., *An investigator-driven study of everolimus in surgical lung biopsy confirmed idiopathic pulmonary fibrosis.* Respirology, 2011. **16**(5): p. 776-83.
310. Sacramento, C.Q., et al., *The in vitro antiviral activity of the anti-hepatitis C virus (HCV) drugs daclatasvir and sofosbuvir agains SARS-CoV-2.* bioRxiv, 2020.
311. Jockusch, S., et al., *Sofosbuvir terminated RNA is more resistant to SARS-CoV-2 proofreader than RNA terminated by Remdesivir.* Sci Rep, 2020. **10**(1): p. 16577.
312. Sadeghi, A., et al., *Sofosbuvir and daclatasvir compared with standard of care in the treatment of patients admitted to hospital with moderate or severe coronavirus infection (COVID-19): a randomized controlled trial.* J Antimicrob Chemother, 2020. **75**(11): p. 3379-3385.
313. Renard, S., et al., *Severe Pulmonary Arterial Hypertension in Patients Treated for Hepatitis C With Sofosbuvir.* Chest, 2016. **149**(3): p. e69-73.
314. Helmers, R.A., et al., *Serious Pulmonary Toxicity Secondary to Novel Hepatitis C Antiviral Therapy in a Liver Transplant Recipient.* Mayo Clin Proc, 2015. **90**(9): p. 1294-7.
315. Zhang, X.J., et al., *In-Hospital Use of Statins Is Associated with a Reduced Risk of Mortality among Individuals with COVID-19.* Cell Metab, 2020. **32**(2): p. 176-187.e4.
316. De Spiegeleer, A., et al., *The Effects of ARBs, ACEis, and Statins on Clinical Outcomes of COVID-19 Infection Among Nursing Home Residents.* J Am Med Dir Assoc, 2020. **21**(7): p. 909-914.e2.
317. Saad, N., et al., *Statins and the risk of interstitial lung disease: a cohort study.* Thorax, 2013. **68**(4): p. 361-4.
318. Kim, S.Y., et al., *A Case of Statin-Induced Interstitial Pneumonitis due to Rosuvastatin.* Tuberc Respir Dis (Seoul), 2015. **78**(3): p. 281-5.
319. Fernández, A.B., et al., *Statins and interstitial lung disease: a systematic review of the literature and of food and drug administration adverse event reports.* Chest, 2008. **134**(4): p. 824-30.
320. Li, L., et al., *Tenofovir alafenamide fumarate attenuates bleomycin-induced pulmonary fibrosis by upregulating the NS5ATP9 and TGF-β1/Smad3 signaling pathway.* Respir Res, 2019. **20**(1): p. 163.
321. Ju, J., et al., *Nucleotide analogues as inhibitors of SARS-CoV Polymerase.* Pharmacol Res Perspect, 2020. **8**(6): p. e00674.
322. Vizcarra, P., et al., *Description of COVID-19 in HIV-infected individuals: a single-centre, prospective cohort.* Lancet HIV, 2020. **7**(8): p. e554-e564.
323. Xie, Q.M., et al., *Pharmacological actions of tetrandrine in inflammatory pulmonary diseases.* Acta Pharmacol Sin, 2002. **23**(12): p. 1107-13.
324. Bhagya, N. and K. Chandrashekar, *Tetrandrine and cancer - An overview on the molecular approach.* Biomed Pharmacother, 2018. **97**: p. 624-632.
325. Chen, Z., et al., *Tetrandrine suppresses lung cancer growth and induces apoptosis, potentially via the VEGF/HIF-1α/ICAM-1 signaling pathway.* Oncol Lett, 2018. **15**(5): p. 7433-7437.
326. Jin, H., et al., *Pulmonary toxicity and metabolic activation of tetrandrine in CD-1 mice.* Chem Res Toxicol, 2011. **24**(12): p. 2142-52.
327. Chen, C., et al., *Thalidomide combined with low-dose short-term glucocorticoid in the treatment of critical Coronavirus Disease 2019.* Clin Transl Med, 2020. **10**(2): p. e35.
328. Zhu, H., et al., *Anti-inflammatory effect of thalidomide on H1N1 influenza virus-induced pulmonary injury in mice.* Inflammation, 2014. **37**(6): p. 2091-8.

329. Horton, M.R., et al., *Thalidomide for the treatment of cough in idiopathic pulmonary fibrosis: a randomized trial.* Ann Intern Med, 2012. **157**(6): p. 398-406.
330. Judson, M.A., et al., *The effect of thalidomide on corticosteroid-dependent pulmonary sarcoidosis.* Sarcoidosis Vasc Diffuse Lung Dis, 2006. **23**(1): p. 51-7.
331. Fazzi, P., et al., *Thalidomide for improving cutaneous and pulmonary sarcoidosis in patients resistant or with contraindications to corticosteroids.* Biomed Pharmacother, 2012. **66**(4): p. 300-7.
332. Khalsa, S.K., C.C. Roberts, and M.S. Underhill, *Acute Pulmonary Toxicity from Thalidomide in a Patient with Multiple Myeloma.* Radiol Case Rep, 2007. **2**(2): p. 41-3.
333. Khalil, A., A. Kamar, and G. Nemer, *Thalidomide-Revisited: Are COVID-19 Patients Going to Be the Latest Victims of Yet Another Theoretical Drug-Repurposing?* Front Immunol, 2020. **11**: p. 1248.
334. Michot, J.M., et al., *Tocilizumab, an anti-IL-6 receptor antibody, to treat COVID-19-related respiratory failure: a case report.* Ann Oncol, 2020. **31**(7): p. 961-964.
335. Xu, X., et al., *Effective treatment of severe COVID-19 patients with tocilizumab.* Proc Natl Acad Sci U S A, 2020. **117**(20): p. 10970-10975.
336. Mastroianni, A., et al., *Subcutaneous tocilizumab treatment in patients with severe COVID-19-related cytokine release syndrome: An observational cohort study.* EClinicalMedicine, 2020. **24**: p. 100410.
337. Guaraldi, G., et al., *Tocilizumab in patients with severe COVID-19: a retrospective cohort study.* Lancet Rheumatol, 2020. **2**(8): p. e474-e484.
338. Toniati, P., et al., *Tocilizumab for the treatment of severe COVID-19 pneumonia with hyperinflammatory syndrome and acute respiratory failure: A single center study of 100 patients in Brescia, Italy.* Autoimmun Rev, 2020. **19**(7): p. 102568.
339. Leisman, D.E., et al., *Cytokine elevation in severe and critical COVID-19: a rapid systematic review, meta-analysis, and comparison with other inflammatory syndromes.* Lancet Respir Med, 2020.
340. Gupta, S., et al., *Association Between Early Treatment With Tocilizumab and Mortality Among Critically Ill Patients With COVID-19.* JAMA Intern Med, 2020.
341. Sinha, P., et al., *Early administration of interleukin-6 inhibitors for patients with severe COVID-19 disease is associated with decreased intubation, reduced mortality, and increased discharge.* Int J Infect Dis, 2020. **99**: p. 28-33.
342. Stone, J.H., et al., *Efficacy of Tocilizumab in Patients Hospitalized with Covid-19.* N Engl J Med, 2020. **383**(24): p. 2333-2344.
343. Silva, S., et al., *Tocilizumab-induced pulmonary fibrosis in a patient with rheumatoid arthritis.* Clin Med (Lond), 2020. **20**(Suppl 2): p. s57.
344. Proskurnina, E.V., et al., *Antioxidant Potential of Antiviral Drug Umifenovir.* Molecules, 2020. **25**(7).
345. Liu, Q., et al., *Antiviral and anti-inflammatory activity of arbidol hydrochloride in influenza A (H1N1) virus infection.* Acta Pharmacol Sin, 2013. **34**(8): p. 1075-83.
346. Zhong, Q., et al., *Antiviral activity of Arbidol against Coxsackie virus B5 in vitro and in vivo.* Arch Virol, 2009. **154**(4): p. 601-7.
347. Brooks, M.J., et al., *Antiviral activity of arbidol, a broad-spectrum drug for use against respiratory viruses, varies according to test conditions.* J Med Virol, 2012. **84**(1): p. 170-81.
348. Yang, C., et al., *Effectiveness of Arbidol for COVID-19 Prevention in Health Professionals.* Front Public Health, 2020. **8**: p. 249.
349. Lian, N., et al., *Umifenovir treatment is not associated with improved outcomes in patients with coronavirus disease 2019: a retrospective study.* Clin Microbiol Infect, 2020. **26**(7): p. 917-921.
350. Johansson, I., et al., *Lower incidence of CMV infection and acute rejections with valganciclovir prophylaxis in lung transplant recipients.* BMC Infect Dis, 2013. **13**: p. 582.
351. Monforte, V., et al., *A multicenter study of valganciclovir prophylaxis up to day 120 in CMV-seropositive lung transplant recipients.* Am J Transplant, 2009. **9**(5): p. 1134-41.
352. DiMaio, M.F., et al., *Effect of zidovudine on human fetal lung development.* Pediatr AIDS HIV Infect, 1995. **6**(2): p. 83-90.
353. Benbrik, E., et al., *Cellular and mitochondrial toxicity of zidovudine (AZT), didanosine (ddI) and zalcitabine (ddC) on cultured human muscle cells.* J Neurol Sci, 1997. **149**(1): p. 19-25.

354. Rachlis, A. and M.M. Fanning, *Zidovudine toxicity. Clinical features and management.* Drug Saf, 1993. **8**(4): p. 312-20.
355. Martínez, E., et al., *Abacavir-based therapy does not affect biological mechanisms associated with cardiovascular dysfunction.* AIDS, 2010. **24**(3).
356. Obel, N., et al., *Abacavir and risk of myocardial infarction in HIV-infected patients on highly active antiretroviral therapy: a population-based nationwide cohort study.* HIV Medicine, 2010. **11**(2): p. 130-136.
357. Group, D.A.D.S., et al., *Use of nucleoside reverse transcriptase inhibitors and risk of myocardial infarction in HIV-infected patients enrolled in the D:A:D study: a multi-cohort collaboration.* Lancet (London, England), 2008. **371**(9622): p. 1417-1426.
358. Hsue, P.Y., et al., *Association of abacavir and impaired endothelial function in treated and suppressed HIV-infected patients.* Aids, 2009. **23**(15): p. 2021-7.
359. Ikonomidis, I., et al., *Inhibition of Interleukin-1 by Anakinra Improves Vascular and Left Ventricular Function in Patients With Rheumatoid Arthritis.* Circulation, 2008. **117**(20): p. 2662-2669.
360. Ling, Y.H., et al., *Anakinra reduces blood pressure and renal fibrosis in one kidney/DOCA/salt-induced hypertension.* Pharmacological Research, 2017. **116**: p. 77-86.
361. Cavalli, G. and C.A. Dinarello, *Anakinra Therapy for Non-cancer Inflammatory Diseases.* Frontiers in Pharmacology, 2018. **9**(1157).
362. Cavalli, G., et al., *Treating Life-Threatening Myocarditis by Blocking Interleukin-1*.* Critical Care Medicine, 2016. **44**(8): p. e751-e754.
363. Movva, R., et al., *Anakinra for myocarditis in juvenile idiopathic arthritis.* Texas Heart Institute journal, 2013. **40**(5): p. 623-625.
364. Trpkov, C., et al., *Rapid Response to Cytokine Storm Inhibition using Anakinra in a Patient with COVID-19 Myocarditis.* CJC Open, 2020.
365. Unsoeld, B., et al., *Abstract 4835: Angiotensin-Converting-Enzyme 2 (rhACE2) Potently Attenuates the Negative Hemodynamic Effects of Angiotensin II (ATII) and Improves Post-Myocardial Infarction (MI) Remodeling.* Circulation, 2008. **118**(suppl_18): p. S_947-S_947.
366. Liu, P., et al., *Novel ACE2-Fc chimeric fusion provides long-lasting hypertension control and organ protection in mouse models of systemic renin angiotensin system activation.* Kidney International, 2018. **94**(1): p. 114-125.
367. Khan, A., et al., *A pilot clinical trial of recombinant human angiotensin-converting enzyme 2 in acute respiratory distress syndrome.* Crit Care, 2017. **21**(1): p. 234.
368. Solzbach, U., et al., *Vitamin C Improves Endothelial Dysfunction of Epicardial Coronary Arteries in Hypertensive Patients.* Circulation, 1997. **96**(5): p. 1513-1519.
369. Aguirre, R. and J.M. May, *Inflammation in the vascular bed: importance of vitamin C.* Pharmacol Ther, 2008. **119**(1): p. 96-103.
370. Moser, M.A. and O.K. Chun, *Vitamin C and Heart Health: A Review Based on Findings from Epidemiologic Studies.* Int J Mol Sci, 2016. **17**(8).
371. Gong, F., et al., *The therapeutic effect of intravenous immunoglobulins and vitamin C on the progression of experimental autoimmune myocarditis in the mouse.* Med Sci Monit, 2007. **13**(11): p. Br240-246.
372. Furtado, R.H.M., et al., *Azithromycin in addition to standard of care versus standard of care alone in the treatment of patients admitted to the hospital with severe COVID-19 in Brazil (COALITION II): a randomised clinical trial.* Lancet, 2020. **396**(10256): p. 959-967.
373. Vouri, S.M., T.N. Thai, and A.G. Winterstein, *An evaluation of co-use of chloroquine or hydroxychloroquine plus azithromycin on cardiac outcomes: A pharmacoepidemiological study to inform use during the COVID19 pandemic.* Res Social Adm Pharm, 2021. **17**(1): p. 2012-2017.
374. Ray, W.A., et al., *Azithromycin and the risk of cardiovascular death.* The New England journal of medicine, 2012. **366**(20): p. 1881-1890.
375. Nidorf, S.M., et al., *Low-Dose Colchicine for Secondary Prevention of Cardiovascular Disease.* Journal of the American College of Cardiology, 2013. **61**(4): p. 404-410.

376. Kezerashvili, A., et al., *Azithromycin as a cause of QT-interval prolongation and torsade de pointes in the absence of other known precipitating factors.* J Interv Card Electrophysiol, 2007. **18**(3): p. 243-6.
377. Pursnani, A., et al., *Hypersensitivity Myocarditis Associated With Azithromycin Exposure.* Annals of Internal Medicine, 2009. **150**(3): p. 225-226.
378. Hayden, F.G. and N. Shindo, *Influenza virus polymerase inhibitors in clinical development.* Current opinion in infectious diseases, 2019. **32**(2): p. 176-186.
379. Qiu, C., et al., *Baricitinib induces LDL-C and HDL-C increases in rheumatoid arthritis: a meta-analysis of randomized controlled trials.* Lipids in Health and Disease, 2019. **18**(1): p. 54.
380. Marty, F.M., et al., *CMX001 to Prevent Cytomegalovirus Disease in Hematopoietic-Cell Transplantation.* New England Journal of Medicine, 2013. **369**(13): p. 1227-1236.
381. Coppini, R., et al., *Pharmacological Inhibition of Serine Proteases to Reduce Cardiac Inflammation and Fibrosis in Atrial Fibrillation.* Frontiers in Pharmacology, 2019. **10**(1420).
382. Tonnesmann, E., R. Kandolf, and T. Lewalter, *Chloroquine cardiomyopathy - a review of the literature.* Immunopharmacol Immunotoxicol, 2013. **35**(3): p. 434-42.
383. Chatre, C., et al., *Cardiac Complications Attributed to Chloroquine and Hydroxychloroquine: A Systematic Review of the Literature.* Drug Safety, 2018. **41**(10): p. 919-931.
384. van den Broek, M.P.H., et al., *Chloroquine-induced QTc prolongation in COVID-19 patients.* Neth Heart J, 2020. **28**(7-8): p. 406-409.
385. Szekely, Y., et al., *Chloroquine-induced torsades de pointes in a patient with coronavirus disease 2019.* Heart rhythm, 2020. **17**(9): p. 1452-1455.
386. Crittenden, D.B., et al., *Colchicine Use Is Associated with Decreased Prevalence of Myocardial Infarction in Patients with Gout.* The Journal of Rheumatology, 2012. **39**(7): p. 1458.
387. Tardif, J.C., et al., *Efficacy and Safety of Low-Dose Colchicine after Myocardial Infarction.* N Engl J Med, 2019. **381**(26): p. 2497-2505.
388. Nidorf, S.M., et al., *Colchicine in Patients with Chronic Coronary Disease.* N Engl J Med, 2020. **383**(19): p. 1838-1847.
389. Lopes, M.I.F., et al., *Beneficial effects of colchicine for moderate to severe COVID-19: an interim analysis of a randomized, double-blinded, placebo controlled clinical trial.* medRxiv, 2020: p. 2020.08.06.20169573.
390. Salehzadeh, F., F. Pourfarzi, and S. Ataei, *The Impact of Colchicine on The COVID-19 Patients; A Clinical Trial Study.* 2020.
391. Smilde, B.J., et al., *Colchicine aggravates coxsackievirus B3 infection in mice.* International Journal of Cardiology, 2016. **216**: p. 58-65.
392. Montseny, J.-J., A. Meyrier, and R.K. Gherardi, *Colchicine toxicity in patients with chronic renal failure.* Nephrology Dialysis Transplantation, 1996. **11**(10): p. 2055-2058.
393. Eleftheriou, G., et al., *Colchicine-induced toxicity in a heart transplant patient with chronic renal failure.* Clinical Toxicology, 2008. **46**(9): p. 827-830.
394. WEIL, M.H., *The Cardiovascular Effects of Corticosteroids.* Circulation, 1962. **25**(4): p. 718-725.
395. Huiart, L., et al., *Low-dose inhaled corticosteroids and the risk of acute myocardial infarction in COPD.* European Respiratory Journal, 2005. **25**(4): p. 634.
396. Belhadjer, Z., et al., *Addition of Corticosteroids to Immunoglobulins Is Associated With Recovery of Cardiac Function in Multi-Inflammatory Syndrome in Children.* Circulation, 2020. **142**(23): p. 2282-2284.
397. Tomasoni, D., et al., *Impact of heart failure on the clinical course and outcomes of patients hospitalized for COVID-19. Results of the Cardio-COVID-Italy multicentre study.* Eur J Heart Fail, 2020.
398. Liu, Q., Y.-h. Zhou, and Z.-q. Yang, *The cytokine storm of severe influenza and development of immunomodulatory therapy.* Cellular & Molecular Immunology, 2016. **13**(1): p. 3-10.
399. Sholter, D.E. and P.W. Armstrong, *Adverse effects of corticosteroids on the cardiovascular system.* Can J Cardiol, 2000. **16**(4): p. 505-11.
400. Andrews, R.C. and B.R. Walker, *Glucocorticoids and insulin resistance: old hormones, new targets.* Clin Sci (Lond), 1999. **96**(5): p. 513-23.

401. van der Hooft, C.S., et al., *Corticosteroids and the Risk of Atrial Fibrillation.* Archives of Internal Medicine, 2006. **166**(9): p. 1016-1020.
402. Ciuca, C., et al., *Myocarditis and coronary aneurysms in a child with acute respiratory syndrome coronavirus 2.* ESC Heart Fail, 2020.
403. Werner, J.C., et al., *Hypertrophic cardiomyopathy associated with dexamethasone therapy for bronchopulmonary dysplasia.* The Journal of Pediatrics, 1992. **120**(2, Part 1): p. 286-291.
404. Ehrmann, D.A., et al., *Impaired β-cell compensation to dexamethasone-induced hyperglycemia in women with polycystic ovary syndrome.* American Journal of Physiology-Endocrinology and Metabolism, 2004. **287**(2): p. E241-E246.
405. Bernal-Mizrachi, C., et al., *Dexamethasone induction of hypertension and diabetes is PPAR-α dependent in LDL receptor–null mice.* Nature Medicine, 2003. **9**(8): p. 1069-1075.
406. Bennet, L., et al., *Temporal changes in fetal cardiovascular, behavioural, metabolic and endocrine responses to maternally administered dexamethasone in the late gestation fetal sheep.* BJOG: An International Journal of Obstetrics & Gynaecology, 1999. **106**(4): p. 331-339.
407. Derks, J.B., et al., *A comparative study of cardiovascular, endocrine and behavioural effects of betamethasone and dexamethasone administration to fetal sheep.* The Journal of Physiology, 1997. **499**(1): p. 217-226.
408. Huffman, J.C. and T.A. Stern, *Disulfiram Use in an Elderly Man With Alcoholism and Heart Disease: A Discussion.* Primary care companion to the Journal of clinical psychiatry, 2003. **5**(1): p. 41-44.
409. Kumaraswamy, G., et al., *A study of cardiovascular complications of disulfiram-ethanol reaction.* National Journal of Physiology, Pharmacy and Pharmacology, 2013. **3**(1): p. 35-42.
410. Barry, W.H., et al., *Nonanticoagulant heparin reduces myocyte Na+ and Ca2+ loading during simulated ischemia and decreases reperfusion injury.* American journal of physiology. Heart and circulatory physiology, 2010. **298**(1): p. H102-H111.
411. Noels, H., C. Weber, and R.R. Koenen, *Chemokines as Therapeutic Targets in Cardiovascular Disease.* Arteriosclerosis, Thrombosis, and Vascular Biology, 2019. **39**(4): p. 583-592.
412. Döring, Y., et al., *The CXCL12/CXCR4 chemokine ligand/receptor axis in cardiovascular disease.* Frontiers in physiology, 2014. **5**: p. 212-212.
413. Liehn, E.A., et al., *Double-edged role of the CXCL12/CXCR4 axis in experimental myocardial infarction.* J Am Coll Cardiol, 2011. **58**(23): p. 2415-23.
414. Lim, S.G., et al., *A Double-blind Placebo-Controlled Study of Emtricitabine in Chronic Hepatitis B.* Archives of Internal Medicine, 2006. **166**(1): p. 49-56.
415. Aberg, J.A., *Cardiovascular complications in HIV management: past, present, and future.* Journal of acquired immune deficiency syndromes (1999), 2009. **50**(1): p. 54-64.
416. Lybaek, D. and C.S. Larsen, *Syncope as a probable side effect to combination antiretroviral therapy initiated during primary HIV-1 infection.* Sex Health, 2008. **5**(1): p. 69-71.
417. Fung, J., et al., *Nucleoside/nucleotide analogues in the treatment of chronic hepatitis B.* Journal of Antimicrobial Chemotherapy, 2011. **66**(12): p. 2715-2725.
418. Kerbaul, F., et al., *How prostacyclin improves cardiac output in right heart failure in conjunction with pulmonary hypertension.* Am J Respir Crit Care Med, 2007. **175**(8): p. 846-50.
419. Page, R.L., et al., *Drugs That May Cause or Exacerbate Heart Failure.* Circulation, 2016. **134**(6): p. e32-e69.
420. Califf, R.M., et al., *A randomized controlled trial of epoprostenol therapy for severe congestive heart failure: The Flolan International Randomized Survival Trial (FIRST).* Am Heart J, 1997. **134**(1): p. 44-54.
421. Nielsen, C.B., J.N. Bech, and E.B. Pedersen, *Epoprostenol increases plasma level of atrial natriuretic peptide in humans.* Clin Sci (Lond), 1996. **90**(2): p. 87-9.
422. Halabi, A. and W. Kirch, *Cardiovascular Effects of Omeprazole and Famotidine.* Scandinavian Journal of Gastroenterology, 1992. **27**(9): p. 753-756.
423. Chinello, P., et al., *QTc interval prolongation during favipiravir therapy in an Ebolavirus-infected patient.* PLoS neglected tropical diseases, 2017. **11**(12): p. e0006034-e0006034.

424. Michaud, V., et al., *Risk Assessment of Drug-Induced Long QT Syndrome for Some COVID-19 Repurposed Drugs.* Clin Transl Sci, 2020.
425. Shapiro, M. and Y. Levy, *The association between hydroxychloroquine treatment and cardiovascular morbidity among rheumatoid arthritis patients.* Oncotarget, 2017. **9**(5): p. 6615-6622.
426. Joyce, E., A. Fabre, and N. Mahon, *Hydroxychloroquine cardiotoxicity presenting as a rapidly evolving biventricular cardiomyopathy: key diagnostic features and literature review.* European heart journal. Acute cardiovascular care, 2013. **2**(1): p. 77-83.
427. Mercuro, N.J., et al., *Risk of QT Interval Prolongation Associated With Use of Hydroxychloroquine With or Without Concomitant Azithromycin Among Hospitalized Patients Testing Positive for Coronavirus Disease 2019 (COVID-19).* JAMA Cardiol, 2020. **5**(9): p. 1036-1041.
428. Chorin, E., et al., *QT interval prolongation and torsade de pointes in patients with COVID-19 treated with hydroxychloroquine/azithromycin.* Heart Rhythm, 2020. **17**(9): p. 1425-1433.
429. Saleh, M., et al., *Effect of Chloroquine, Hydroxychloroquine, and Azithromycin on the Corrected QT Interval in Patients With SARS-CoV-2 Infection.* Circ Arrhythm Electrophysiol, 2020. **13**(6): p. e008662.
430. Jain, S., et al., *Enhanced electrocardiographic monitoring of patients with Coronavirus Disease 2019.* Heart rhythm, 2020. **17**(9): p. 1417-1422.
431. Jankelson, L., et al., *QT prolongation, torsades de pointes, and sudden death with short courses of chloroquine or hydroxychloroquine as used in COVID-19: A systematic review.* Heart Rhythm, 2020. **17**(9): p. 1472-1479.
432. Olson, N.C., et al., *Soluble CD14, Ischemic Stroke, and Coronary Heart Disease Risk in a Prospective Study: The REGARDS Cohort.* Journal of the American Heart Association, 2020. **9**(6): p. e014241.
433. Al-Kindi, S.G., et al., *Soluble CD14 and Risk of Heart Failure and Its Subtypes in Older Adults.* Journal of Cardiac Failure, 2020. **26**(5): p. 410-419.
434. Genth-Zotz, S., et al., *The anti-CD14 antibody IC14 suppresses ex vivo endotoxin stimulated tumor necrosis factor-alpha in patients with chronic heart failure.* Eur J Heart Fail, 2006. **8**(4): p. 366-72.
435. Salles, G., et al., *Efficacy and safety of idelalisib in patients with relapsed, rituximab- and alkylating agent-refractory follicular lymphoma: a subgroup analysis of a phase 2 study.* Haematologica, 2017. **102**(4): p. e156-e159.
436. Pereda, R., et al., *Therapeutic effectiveness of interferon alpha 2b treatment for COVID-19 patient recovery.* medRxiv, 2020: p. 2020.07.28.20157974.
437. Daliento, L., et al., *Successful treatment of enterovirus-induced myocarditis with interferon-α.* The Journal of Heart and Lung Transplantation, 2003. **22**(2): p. 214-217.
438. Sonnenblick, M. and A. Rosin, *Cardiotoxicity of Interferon*: A Review of 44 Cases.* Chest, 1991. **99**(3): p. 557-561.
439. Sparsa, A., et al., *[Systemic adverse reactions with ivermectin treatment of scabies].* Ann Dermatol Venereol, 2006. **133**(10): p. 784-7.
440. Goa, K.L., D. McTavish, and S.P. Clissold, *Ivermectin.* Drugs, 1991. **42**(4): p. 640-658.
441. Dobaczewski, M., et al., *CCR5 signaling suppresses inflammation and reduces adverse remodeling of the infarcted heart, mediating recruitment of regulatory T cells.* Am J Pathol, 2010. **176**(5): p. 2177-87.
442. Cao, B., et al., *A Trial of Lopinavir-Ritonavir in Adults Hospitalized with Severe Covid-19.* The New England journal of medicine, 2020. **382**(19): p. 1787-1799.
443. Shree, V., et al., *Potentially fatal severe brady arrythmias related to Lopinavir-Ritonavir in a COVID 19 patient.* J Microbiol Immunol Infect, 2020.
444. Soliman, E.Z., et al., *Boosted protease inhibitors and the electrocardiographic measures of QT and PR durations.* AIDS, 2011. **25**(3).
445. Charbit, B., et al., *Relationship between HIV protease inhibitors and QTc interval duration in HIV-infected patients: a cross-sectional study.* British Journal of Clinical Pharmacology, 2009. **67**(1): p. 76-82.
446. Zhong, D.-s., et al., *HIV Protease Inhibitor Ritonavir Induces Cytotoxicity of Human Endothelial Cells.* Arteriosclerosis, Thrombosis, and Vascular Biology, 2002. **22**(10): p. 1560-1566.

447. Busuttil, R.W., W.J. George, and R.L. Hewitt, *Protective effect of methylprednisolone on the heart during ischemic arrest.* The Journal of Thoracic and Cardiovascular Surgery, 1975. **70**(6): p. 955-965.
448. Watanabe, N., et al., *Acute necrotizing eosinophilic myocarditis successfully treated by high dose methylprednisolone.* Jpn Circ J, 2001. **65**(10): p. 923-6.
449. Jühl, U., B.E. Strauer, and H.P. Schultheiss, *Methylprednisolone in chronic myocarditis.* Postgraduate medical journal, 1994. **70 Suppl 1**: p. S35-42.
450. Li, A., et al., *Management of COVID-19 myopericarditis with reversal of cardiac dysfunction after blunting of cytokine storm: a case report.* Eur Heart J Case Rep, 2020. **4**(Fi1): p. 1-6.
451. Naneishvili, T., et al., *Fulminant myocarditis as an early presentation of SARS-CoV-2.* BMJ Case Rep, 2020. **13**(9).
452. Romano, C., et al., *Atrial fibrillation following therapy with high-dose i.v. methylprednisolone: A brief case-based review.* Eur J Rheumatol, 2017. **4**(3): p. 231-233.
453. Kim, H.S., et al., *Cardiac arrest caused by nafamostat mesilate.* Kidney Res Clin Pract, 2016. **35**(3): p. 187-9.
454. Chen, W., et al., *Niclosamide: Beyond an antihelminthic drug.* Cellular signalling, 2018. **41**: p. 89-96.
455. Li, Y., et al., *Multi-targeted therapy of cancer by niclosamide: A new application for an old drug.* Cancer Lett, 2014. **349**(1): p. 8-14.
456. Foulquier, S., et al., *WNT Signaling in Cardiac and Vascular Disease.* Pharmacological Reviews, 2018. **70**(1): p. 68.
457. Li, S.L., et al., *Niclosamide ethanolamine inhibits artery constriction.* Pharmacol Res, 2017. **115**: p. 78-86.
458. Täubel, J., et al., *Analyzing the relationship of QT interval and exposure to nitazoxanide, a prospective candidate for influenza antiviral therapy--A formal TQT study.* J Clin Pharmacol, 2014. **54**(9): p. 987-94.
459. Gao, Y., et al. *Effect of Nitazoxanide on cardiovascular system , respiratory system and nervous system*. 2014.
460. Ignarro, L.J., et al., *Endothelium-derived relaxing factor produced and released from artery and vein is nitric oxide.* Proc Natl Acad Sci U S A, 1987. **84**(24): p. 9265-9.
461. d'Uscio, L.V., et al., *Long-term vitamin C treatment increases vascular tetrahydrobiopterin levels and nitric oxide synthase activity.* Circ Res, 2003. **92**(1): p. 88-95.
462. Münzel, T., et al., *Vascular consequences of endothelial nitric oxide synthase uncoupling for the activity and expression of the soluble guanylyl cyclase and the cGMP-dependent protein kinase.* Arterioscler Thromb Vasc Biol, 2005. **25**(8): p. 1551-7.
463. Jones, S.P. and R. Bolli, *The ubiquitous role of nitric oxide in cardioprotection.* J Mol Cell Cardiol, 2006. **40**(1): p. 16-23.
464. Zamanian, R.T., et al., *Outpatient Inhaled Nitric Oxide in a Patient with Vasoreactive Idiopathic Pulmonary Arterial Hypertension and COVID-19 Infection.* Am J Respir Crit Care Med, 2020. **202**(1): p. 130-132.
465. Ishiyama, S., et al., *Nitric Oxide Contributes to the Progression of Myocardial Damage in Experimental Autoimmune Myocarditis in Rats.* Circulation, 1997. **95**(2): p. 489-496.
466. Yu, W.-L., et al., *A Double-Edged Sword—Cardiovascular Concerns of Potential Anti-COVID-19 Drugs.* Cardiovascular Drugs and Therapy, 2020.
467. Crotti, L. and E. Arbelo, *COVID-19 treatments, QT interval, and arrhythmic risk: The need for an international registry on arrhythmias.* Heart rhythm, 2020. **17**(9): p. 1423-1424.
468. Mulangu, S., et al., *A Randomized, Controlled Trial of Ebola Virus Disease Therapeutics.* New England Journal of Medicine, 2019. **381**(24): p. 2293-2303.
469. Zampino, R., et al., *Liver injury in remdesivir-treated COVID-19 patients.* Hepatology International, 2020. **14**(5): p. 881-883.
470. Durante-Mangoni, E., et al., *Early experience with remdesivir in SARS-CoV-2 pneumonia.* Infection, 2020. **48**(5): p. 779-782.
471. Gupta, A.K., et al., *Cardiac Adverse Events With Remdesivir in COVID-19 Infection.* Cureus, 2020. **12**(10): p. e11132.

472. Miyawaki, H., et al., *Long-term Effects of the Janus Kinase 1/2 Inhibitor Ruxolitinib on Pulmonary Hypertension and the Cardiac Function in a Patient with Myelofibrosis.* Internal medicine (Tokyo, Japan), 2020. **59**(2): p. 229-233.
473. Mascarenhas, J. and R. Hoffman, *A comprehensive review and analysis of the effect of ruxolitinib therapy on the survival of patients with myelofibrosis.* Blood, 2013. **121**(24): p. 4832-4837.
474. Fontes, J.A., N.R. Rose, and D. Čiháková, *The varying faces of IL-6: From cardiac protection to cardiac failure.* Cytokine, 2015. **74**(1): p. 62-68.
475. Sciarretta, S., et al., *New Insights Into the Role of mTOR Signaling in the Cardiovascular System.* Circulation research, 2018. **122**(3): p. 489-505.
476. Miller, L.W., *Cardiovascular toxicities of immunosuppressive agents.* Am J Transplant, 2002. **2**(9): p. 807-18.
477. Alvarez-Alvarez, R.J., et al., *Venous thromboembolism in heart transplant recipients: incidence, recurrence and predisposing factors.* J Heart Lung Transplant, 2015. **34**(2): p. 167-74.
478. Thibodeau, J.T., et al., *Sirolimus use and incidence of venous thromboembolism in cardiac transplant recipients.* Clinical Transplantation, 2012. **26**(6): p. 953-959.
479. Mazzitelli, M., et al., *Evaluation of cardiac function by global longitudinal strain before and after treatment with sofosbuvir-based regimens in HCV infected patients.* BMC Infectious Diseases, 2018. **18**(1): p. 518.
480. Bagate, F., et al., *0428: Cardiac arrhythmia after initiation of sofosbuvir-including regimen in patients with chronic hepatitis C virus infection.* Archives of Cardiovascular Diseases Supplements, 2016. **8**(1): p. 76.
481. Millard, D.C., et al., *Identification of Drug–Drug Interactions In Vitro: A Case Study Evaluating the Effects of Sofosbuvir and Amiodarone on hiPSC-Derived Cardiomyocytes.* Toxicological Sciences, 2016. **154**(1): p. 174-182.
482. Back, D.J. and D.M. Burger, *Interaction between amiodarone and sofosbuvir-based treatment for hepatitis C virus infection: potential mechanisms and lessons to be learned.* Gastroenterology, 2015. **149**(6): p. 1315-7.
483. Hundemer, G.L., et al., *Use of sofosbuvir-based direct-acting antiviral therapy for hepatitis C viral infection in patients with severe renal insufficiency.* Infectious Diseases, 2015. **47**(12): p. 924-929.
484. Cox-North, P., et al., *Sofosbuvir-based regimens for the treatment of chronic hepatitis C in severe renal dysfunction.* Hepatol Commun, 2017. **1**(3): p. 248-255.
485. Nissen, S.E., et al., *Statin Therapy, LDL Cholesterol, C-Reactive Protein, and Coronary Artery Disease.* New England Journal of Medicine, 2005. **352**(1): p. 29-38.
486. Jain, M.K. and P.M. Ridker, *Anti-Inflammatory Effects of Statins: Clinical Evidence and Basic Mechanisms.* Nature Reviews Drug Discovery, 2005. **4**(12): p. 977-987.
487. Lazzerini, P.E., P.L. Capecchi, and F. Laghi-Pasini, *Statins as a New Therapeutic Perspective in Myocarditis and Postmyocarditis Dilated Cardiomyopathy.* Cardiovascular Drugs and Therapy, 2013. **27**(5): p. 365-369.
488. Zhang, X.-J., et al., *In-Hospital Use of Statins Is Associated with a Reduced Risk of Mortality among Individuals with COVID-19.* Cell Metabolism, 2020. **32**(2): p. 176-187.e4.
489. Bavinger, C., et al., *Risk of cardiovascular disease from antiretroviral therapy for HIV: a systematic review.* PloS one, 2013. **8**(3): p. e59551-e59551.
490. Chen, R., et al., *Association of Tenofovir Use With Risk of Incident Heart Failure in HIV‐Infected Patients.* Journal of the American Heart Association, 2017. **6**(4): p. e005387.
491. Choi, A.I., et al., *Cardiovascular risks associated with abacavir and tenofovir exposure in HIV-infected persons.* AIDS (London, England), 2011. **25**(10): p. 1289-1298.
492. Aragonès, G., et al., *The deleterious influence of tenofovir-based therapies on the progression of atherosclerosis in HIV-infected patients.* Mediators Inflamm, 2012. **2012**: p. 372305.
493. Teng, G., et al., *Tetrandrine reverses human cardiac myofibroblast activation and myocardial fibrosis.* American Journal of Physiology-Heart and Circulatory Physiology, 2015. **308**(12): p. H1564-H1574.

494. Pinelli, A., et al., *Pretreatment with tetrandrine has protective effects against isoproterenol-induced myocardial infarction in rabbits.* In Vivo, 2010. **24**(3): p. 265-70.
495. Shen, Y.-C., C.-F. Chen, and Y.-J. Sung, *Tetrandrine ameliorates ischaemia-reperfusion injury of rat myocardium through inhibition of neutrophil priming and activation.* British Journal of Pharmacology, 1999. **128**(7): p. 1593-1601.
496. Gullestad, L., et al., *Effect of thalidomide in patients with chronic heart failure.* American Heart Journal, 2002. **144**(5): p. 847-850.
497. Gullestad, L., et al., *Effect of thalidomide on cardiac remodeling in chronic heart failure: results of a double-blind, placebo-controlled study.* Circulation, 2005. **112**(22): p. 3408-14.
498. Ueland, T., et al., *Inflammatory cytokines as biomarkers in heart failure.* Clin Chim Acta, 2015. **443**: p. 71-7.
499. Ghobrial, I.M. and S.V. Rajkumar, *Management of thalidomide toxicity.* The journal of supportive oncology, 2003. **1**(3): p. 194-205.
500. Arboscello, E., et al., *Thalidomide-induced cardiotoxicity in multiple myeloma patients: An underestimated but clinically relevant issue.* Journal of Clinical Oncology, 2010. **28**(15_suppl): p. e18544-e18544.
501. Navarro, G., et al., *Tocilizumab in rheumatoid arthritis: a meta-analysis of efficacy and selected clinical conundrums.* Semin Arthritis Rheum, 2014. **43**(4): p. 458-69.
502. Coyle, J., et al., *A Recovered Case of COVID-19 Myocarditis and ARDS Treated With Corticosteroids, Tocilizumab, and Experimental AT-001.* JACC: Case Reports, 2020. **2**(9): p. 1331-1336.
503. Bacchiega, B.C., et al., *Interleukin 6 Inhibition and Coronary Artery Disease in a High‐Risk Population: A Prospective Community‐Based Clinical Study.* Journal of the American Heart Association, 2017. **6**(3): p. e005038.
504. Giles, J.T., et al., *Cardiovascular Safety of Tocilizumab Versus Etanercept in Rheumatoid Arthritis: A Randomized Controlled Trial.* Arthritis Rheumatol, 2020. **72**(1): p. 31-40.
505. Cacciapaglia, F., et al., *Lipids and Atherogenic Indices Fluctuation in Rheumatoid Arthritis Patients on Long-Term Tocilizumab Treatment.* Mediators of Inflammation, 2018. **2018**.
506. Antinori, S., et al., *Tocilizumab for cytokine storm syndrome in COVID-19 pneumonia: an increased risk for candidemia?* Autoimmunity reviews, 2020. **19**(7): p. 102564-102564.
507. Salvarani, C., et al., *Effect of Tocilizumab vs Standard Care on Clinical Worsening in Patients Hospitalized With COVID-19 Pneumonia: A Randomized Clinical Trial.* JAMA Internal Medicine, 2020.
508. Naksuk, N., S. Lazar, and T.B. Peeraphatdit, *Cardiac safety of off-label COVID-19 drug therapy: a review and proposed monitoring protocol.* European heart journal. Acute cardiovascular care, 2020. **9**(3): p. 215-221.
509. Zhong, Q., et al., *Antiviral activity of Arbidol against Coxsackie virus B5 in vitro and in vivo.* Archives of virology, 2009. **154**(4): p. 601-607.
510. Luna, E., et al., *Effect of Cytomegalovirus Infection on Survival of Older Kidney Transplant Patients (D+/R+): Impact of Valganciclovir Prophylaxis Versus Preemptive Therapy.* Transplant Proc, 2016. **48**(9): p. 2931-2937.
511. Padala, S.K., A. Kumar, and S. Padala, *Fulminant Cytomegalovirus Myocarditis in an Immunocompetent Host: Resolution with Oral Valganciclovir.* Texas Heart Institute Journal, 2014. **41**(5): p. 523-529.
512. Wilkins, C.E., D.J. Sexton, and H.A. McAllister, *HIV-associated myocarditis treated with zidovudine (AZT).* Texas Heart Institute journal, 1989. **16**(1): p. 44-45.
513. Papparella, I., et al., *Vitamin C prevents zidovudine-induced NAD(P)H oxidase activation and hypertension in the rat.* Cardiovascular Research, 2007. **73**(2): p. 432-438.
514. Domanski, M.J., et al., *Effect of zidovudine and didanosine treatment on heart function in children infected with human immunodeficiency virus.* The Journal of Pediatrics, 1995. **127**(1): p. 137-146.
515. Herskowitz, A., et al., *Cardiomyopathy Associated with Antiretroviral Therapy in Patients with HIV Infection: A Report of Six Cases.* Annals of Internal Medicine, 1992. **116**(4): p. 311-313.

516. Barbaro, G., et al., *Incidence of Dilated Cardiomyopathy and Detection of HIV in Myocardial Cells of HIV-Positive Patients.* New England Journal of Medicine, 1998. **339**(16): p. 1093-1099.
517. Flandre, P., et al., *Risk factors of chronic kidney disease in HIV-infected patients.* Clin J Am Soc Nephrol, 2011. **6**(7): p. 1700-7.
518. Rusai, K., et al., *Administration of interleukin-1 receptor antagonist ameliorates renal ischemia-reperfusion injury.* Transpl Int, 2008. **21**(6): p. 572-80.
519. Mulders-Manders, C.M., et al., *Peri- and Postoperative Treatment with the Interleukin-1 Receptor Antagonist Anakinra Is Safe in Patients Undergoing Renal Transplantation: Case Series and Review of the Literature.* Front Pharmacol, 2017. **8**: p. 342.
520. Ling, Y.H., et al., *Anakinra reduces blood pressure and renal fibrosis in one kidney/DOCA/salt-induced hypertension.* Pharmacol Res, 2017. **116**: p. 77-86.
521. Chen, L.J., et al., *Angiotensin-converting enzyme 2 ameliorates renal fibrosis by blocking the activation of mTOR/ERK signaling in apolipoprotein E-deficient mice.* Peptides, 2016. **79**: p. 49-57.
522. Liu, P., et al., *Novel ACE2-Fc chimeric fusion provides long-lasting hypertension control and organ protection in mouse models of systemic renin angiotensin system activation.* Kidney Int, 2018. **94**(1): p. 114-125.
523. Lin, T.J., et al., *Optimal dosage and early intervention of L-ascorbic acid inhibiting K.* J Trace Elem Med Biol, 2018. **48**: p. 1-7.
524. Lin, W.V., et al., *Ascorbic acid-induced oxalate nephropathy: a case report and discussion of pathologic mechanisms.* CEN Case Rep, 2019. **8**(1): p. 67-70.
525. Fontana, F., et al., *Oxalate Nephropathy Caused by Excessive Vitamin C Administration in 2 Patients With COVID-19.* Kidney Int Rep, 2020. **5**(10): p. 1815-1822.
526. McHugh, G.J., M.L. Graber, and R.C. Freebairn, *Fatal vitamin C-associated acute renal failure.* Anaesth Intensive Care, 2008. **36**(4): p. 585-8.
527. Mashour, S., J.F. Turner, and R. Merrell, *Acute renal failure, oxalosis, and vitamin C supplementation: a case report and review of the literature.* Chest, 2000. **118**(2): p. 561-3.
528. Rathi, S., W. Kern, and K. Lau, *Vitamin C-induced hyperoxaluria causing reversible tubulointerstitial nephritis and chronic renal failure: a case report.* J Med Case Rep, 2007. **1**: p. 155.
529. McAllister, C.J., et al., *Renal failure secondary to massive infusion of vitamin C.* JAMA, 1984. **252**(13): p. 1684.
530. Gandhi, S., et al., *Calcium-channel blocker-clarithromycin drug interactions and acute kidney injury.* JAMA, 2013. **310**(23): p. 2544-53.
531. Sakurai, T., et al., *Imaging Mass Microscopy of Kidneys from Azithromycin-Treated Rats with Phospholipidosis.* Am J Pathol, 2018. **188**(9): p. 1993-2003.
532. Ng, K.E., *Xofluza (Baloxavir Marboxil) for the Treatment Of Acute Uncomplicated Influenza.* P T, 2019. **44**(1): p. 9-11.
533. Tuttle, K.R., et al., *JAK1/JAK2 inhibition by baricitinib in diabetic kidney disease: results from a Phase 2 randomized controlled clinical trial.* Nephrol Dial Transplant, 2018. **33**(11): p. 1950-1959.
534. Faure, E., et al., *Case report: Brincidofovir-induced reversible severe acute kidney injury in 2 solid-organ transplant for treatment of cytomegalovirus infection.* Medicine (Baltimore), 2016. **95**(44): p. e5226.
535. Vora, S.B., A.W. Brothers, and J.A. Englund, *Renal Toxicity in Pediatric Patients Receiving Cidofovir for the Treatment of Adenovirus Infection.* J Pediatric Infect Dis Soc, 2017. **6**(4): p. 399-402.
536. Lacy, S.A., et al., *Effect of oral probenecid coadministration on the chronic toxicity and pharmacokinetics of intravenous cidofovir in cynomolgus monkeys.* Toxicol Sci, 1998. **44**(2): p. 97-106.
537. Onbe, T., et al., *Effect of proteinase inhibitor camostat mesilate on nephrotic syndrome with diabetic nephropathy.* J Diabet Complications, 1991. **5**(2-3): p. 167-8.
538. Ueda, M., et al., *The serine protease inhibitor camostat mesilate attenuates the progression of chronic kidney disease through its antioxidant effects.* Nephron, 2015. **129**(3): p. 223-32.
539. Maekawa, A., et al., *Camostat mesilate inhibits prostasin activity and reduces blood pressure and renal injury in salt-sensitive hypertension.* J Hypertens, 2009. **27**(1): p. 181-9.

540. Kakizoe, Y., et al., *A serine protease inhibitor attenuates aldosterone-induced kidney injuries via the suppression of plasmin activity.* J Pharmacol Sci, 2016. **132**(2): p. 145-153.
541. Ngaha, E.O., *Some biochemical changes in the rat during repeated chloroquine administration.* Toxicol Lett, 1982. **10**(2-3): p. 145-9.
542. Albay, D., et al., *Chloroquine-induced lipidosis mimicking Fabry disease.* Mod Pathol, 2005. **18**(5): p. 733-8.
543. Thorogood, N., et al., *The risk of antimalarials in patients with renal failure.* Postgrad Med J, 2007. **83**(986): p. e8.
544. Solak, Y., et al., *Colchicine in Renal Medicine: New Virtues of an Ancient Friend.* Blood Purif, 2017. **43**(1-3): p. 125-135.
545. Itano, S., et al., *Colchicine attenuates renal fibrosis in a murine unilateral ureteral obstruction model.* Mol Med Rep, 2017. **15**(6): p. 4169-4175.
546. Medani, S. and C. Wall, *Colchicine toxicity in renal patients - Are we paying attention?* Clin Nephrol, 2016. **86**(2): p. 100-5.
547. Cai, Q., et al., *Severe Adverse Effects Associated With Corticosteroid Treatment in Patients With IgA Nephropathy.* Kidney Int Rep, 2017. **2**(4): p. 603-609.
548. Jacob, K.A., et al., *Intraoperative High-Dose Dexamethasone and Severe AKI after Cardiac Surgery.* J Am Soc Nephrol, 2015. **26**(12): p. 2947-51.
549. Sato, Y., et al., *Heterogeneous fibroblasts underlie age-dependent tertiary lymphoid tissues in the kidney.* JCI Insight, 2016. **1**(11): p. e87680.
550. Johnson, T.S., et al., *Transglutaminase inhibition reduces fibrosis and preserves function in experimental chronic kidney disease.* J Am Soc Nephrol, 2007. **18**(12): p. 3078-88.
551. Huang, L., et al., *Transglutaminase inhibition ameliorates experimental diabetic nephropathy.* Kidney Int, 2009. **76**(4): p. 383-94.
552. Palanski, B.A. and C. Khosla, *Cystamine and Disulfiram Inhibit Human Transglutaminase 2 via an Oxidative Mechanism.* Biochemistry, 2018. **57**(24): p. 3359-3363.
553. Wang, A., et al., *CXCR4/CXCL12 hyperexpression plays a pivotal role in the pathogenesis of lupus.* J Immunol, 2009. **182**(7): p. 4448-58.
554. Solomon, M.M., et al., *Changes in renal function associated with oral emtricitabine/tenofovir disoproxil fumarate use for HIV pre-exposure prophylaxis.* AIDS, 2014. **28**(6): p. 851-9.
555. Godinho, R., et al., *Severe rhabdomyolysis-induced acute kidney injury following concomitant use of Genvoya® (EVG/COBI/FTC/TAF) and simvastatin; a case report.* BMC Nephrol, 2019. **20**(1): p. 69.
556. Tsai, M.C., et al., *Comparison of renal safety and efficacy of telbivudine, entecavir and tenofovir treatment in chronic hepatitis B patients: real world experience.* Clin Microbiol Infect, 2016. **22**(1): p. 95.e1-95.e7.
557. Uteng, M., et al., *Editor's Highlight: Comparative Renal Safety Assessment of the Hepatitis B Drugs, Adefovir, Tenofovir, Telbivudine and Entecavir in Rats.* Toxicol Sci, 2017. **155**(1): p. 283-297.
558. Nielsen, C.B., J.N. Bech, and E.B. Pedersen, *Effects of prostacyclin on renal haemodynamics, renal tubular function and vasoactive hormones in healthy humans. A placebo-controlled dose-response study.* Br J Clin Pharmacol, 1997. **44**(5): p. 471-6.
559. Ogawa, N., et al., *Plasma levels of natriuretic peptides and development of chronic kidney disease.* BMC Nephrol, 2015. **16**: p. 171.
560. Koitka, A., et al., *Angiotensin converting enzyme 2 in the kidney.* Clin Exp Pharmacol Physiol, 2008. **35**(4): p. 420-5.
561. Abraham, P.A., et al., *The effect of famotidine on renal function in patients with renal insufficiency.* Br J Clin Pharmacol, 1987. **24**(3): p. 385-9.
562. Kerber, R., et al., *Laboratory Findings, Compassionate Use of Favipiravir, and Outcome in Patients With Ebola Virus Disease, Guinea, 2015-A Retrospective Observational Study.* J Infect Dis, 2019. **220**(2): p. 195-202.
563. Dirim, A.B., et al., *Fatal SARS-CoV-2 infection in a renal transplant recipient.* CEN Case Rep, 2020. **9**(4): p. 409-412.

564. Thammathiwat, T., et al., *A case of successful treatment of severe COVID-19 pneumonia with favipiravir and tocilizumab in post-kidney transplant recipient.* Transpl Infect Dis, 2020: p. e13388.
565. Pons-Estel, G.J., et al., *Protective effect of hydroxychloroquine on renal damage in patients with lupus nephritis: LXV, data from a multiethnic US cohort.* Arthritis Rheum, 2009. **61**(6): p. 830-9.
566. Lafyatis, R., M. York, and A. Marshak-Rothstein, *Antimalarial agents: closing the gate on Toll-like receptors?* Arthritis Rheum, 2006. **54**(10): p. 3068-70.
567. Smith, K.D., *Lupus nephritis: toll the trigger!* J Am Soc Nephrol, 2006. **17**(12): p. 3273-5.
568. Karres, I., et al., *Chloroquine inhibits proinflammatory cytokine release into human whole blood.* Am J Physiol, 1998. **274**(4): p. R1058-64.
569. Baccala, R., et al., *TLR-dependent and TLR-independent pathways of type I interferon induction in systemic autoimmunity.* Nat Med, 2007. **13**(5): p. 543-51.
570. Wallace, D.J., *Does hydroxychloroquine sulfate prevent clot formation in systemic lupus erythematosus?* Arthritis Rheum, 1987. **30**(12): p. 1435-6.
571. Petri, M., *Hydroxychloroquine use in the Baltimore Lupus Cohort: effects on lipids, glucose and thrombosis.* Lupus, 1996. **5 Suppl 1**: p. S16-22.
572. Gerstein, H.C., et al., *The effectiveness of hydroxychloroquine in patients with type 2 diabetes mellitus who are refractory to sulfonylureas--a randomized trial.* Diabetes Res Clin Pract, 2002. **55**(3): p. 209-19.
573. Petri, M., *Thrombosis and systemic lupus erythematosus: the Hopkins Lupus Cohort perspective.* Scand J Rheumatol, 1996. **25**(4): p. 191-3.
574. Tang, T.T., et al., *Hydroxychloroquine attenuates renal ischemia/reperfusion injury by inhibiting cathepsin mediated NLRP3 inflammasome activation.* Cell Death Dis, 2018. **9**(3): p. 351.
575. Zhou, J., et al., *Renal CD14 expression correlates with the progression of cystic kidney disease.* Kidney Int, 2010. **78**(6): p. 550-60.
576. D'Aveni-Piney, M., et al., *Idelalisib in a patient with refractory Waldenström's macroglobulinemia complicated by anuric renal failure: a case report.* J Med Case Rep, 2018. **12**(1): p. 164.
577. Wanchoo, R., et al., *Renal involvement in chronic lymphocytic leukemia.* Clin Kidney J, 2018. **11**(5): p. 670-680.
578. Fosså, S.D., *Interferon in metastatic renal cell carcinoma.* Semin Oncol, 2000. **27**(2): p. 187-93.
579. Markowitz, G.S., et al., *Treatment with IFN-{alpha}, -{beta}, or -{gamma} is associated with collapsing focal segmental glomerulosclerosis.* Clin J Am Soc Nephrol, 2010. **5**(4): p. 607-15.
580. Fairhurst, A.M., et al., *Systemic IFN-alpha drives kidney nephritis in B6.Sle123 mice.* Eur J Immunol, 2008. **38**(7): p. 1948-60.
581. Zhu, M., Y. Li, and Z. Zhou, *Antibiotic ivermectin preferentially targets renal cancer through inducing mitochondrial dysfunction and oxidative damage.* Biochem Biophys Res Commun, 2017. **492**(3): p. 373-378.
582. Segerer, S., et al., *Expression of the C-C chemokine receptor 5 in human kidney diseases.* Kidney Int, 1999. **56**(1): p. 52-64.
583. Lefebvre, E., et al., *Antifibrotic Effects of the Dual CCR2/CCR5 Antagonist Cenicriviroc in Animal Models of Liver and Kidney Fibrosis.* PLoS One, 2016. **11**(6): p. e0158156.
584. Kiser, J.J., et al., *The effect of lopinavir/ritonavir on the renal clearance of tenofovir in HIV-infected patients.* Clin Pharmacol Ther, 2008. **83**(2): p. 265-72.
585. Rollot, F., et al., *Tenofovir-related Fanconi syndrome with nephrogenic diabetes insipidus in a patient with acquired immunodeficiency syndrome: the role of lopinavir-ritonavir-didanosine.* Clin Infect Dis, 2003. **37**(12): p. e174-6.
586. Shafi, T., et al., *Ritonavir-induced acute kidney injury: kidney biopsy findings and review of literature.* Clin Nephrol, 2011. **75 Suppl 1**: p. 60-4.
587. Ryom, L., et al., *Association between antiretroviral exposure and renal impairment among HIV-positive persons with normal baseline renal function: the D:A:D study.* J Infect Dis, 2013. **207**(9): p. 1359-69.
588. You, Y., et al., *Methylprednisolone attenuates lipopolysaccharide-induced Fractalkine expression in kidney of Lupus-prone MRL/lpr mice through the NF-kappaB pathway.* BMC Nephrol, 2015. **16**: p. 148.

589. Sakemi, T., et al., *Effects of the methylprednisolone pulse therapy on renal function.* Am J Nephrol, 1991. **11**(1): p. 48-53.
590. Choi, J.Y., et al., *Nafamostat Mesilate as an Anticoagulant During Continuous Renal Replacement Therapy in Patients With High Bleeding Risk: A Randomized Clinical Trial.* Medicine (Baltimore), 2015. **94**(52): p. e2392.
591. Minakata, D., et al., *Comparison of gabexate mesilate and nafamostat mesilate for disseminated intravascular coagulation associated with hematological malignancies.* Int J Hematol, 2019. **109**(2): p. 141-146.
592. Iwashita, K., et al., *Inhibition of prostasin secretion by serine protease inhibitors in the kidney.* J Am Soc Nephrol, 2003. **14**(1): p. 11-6.
593. Yu, X., et al., *Niclosamide Exhibits Potent Anticancer Activity and Synergizes with Sorafenib in Human Renal Cell Cancer Cells.* Cell Physiol Biochem, 2018. **47**(3): p. 957-971.
594. Chang, X., et al., *The antihelmenthic phosphate niclosamide impedes renal fibrosis by inhibiting homeodomain-interacting protein kinase 2 expression.* Kidney Int, 2017. **92**(3): p. 612-624.
595. Han, P., et al., *Niclosamide ethanolamine improves kidney injury in db/db mice.* Diabetes Res Clin Pract, 2018. **144**: p. 25-33.
596. Liu, Y., et al., *Extracellular ATP drives breast cancer cell migration and metastasis via S100A4 production by cancer cells and fibroblasts.* Cancer Lett, 2018. **430**: p. 1-10.
597. Ghusson, N. and G. Vasquez, *Successfully Treated Norovirus- and Sapovirus-Associated Diarrhea in Three Renal Transplant Patients.* Case Rep Infect Dis, 2018. **2018**: p. 6846873.
598. El-Din Shams, G., A. El-Alim Fouad, and N. Naiem, *Nitazoxanide Adverse Effects on Biochemical Markers of Liver & Kidney Injury and Antioxidant Enzymes on Rats.* International Journal of Pharmaceutical Research & Allied Sciences, 2018. **7**(4).
599. Ahmad, A., et al., *Role of Nitric Oxide in the Cardiovascular and Renal Systems.* Int J Mol Sci, 2018. **19**(9).
600. Chintala, M.S., et al., *Inhibition of endothelial derived relaxing factor (EDRF) aggravates ischemic acute renal failure in anesthetized rats.* Naunyn Schmiedebergs Arch Pharmacol, 1993. **348**(3): p. 305-10.
601. Thakare, S., et al., *Safety of Remdesivir in Patients with Acute or Chronic Kidney Disease.* Kidney Int Rep, 2020.
602. Adamsick, M.L., et al., *Remdesivir in Patients with Acute or Chronic Kidney Disease and COVID-19.* J Am Soc Nephrol, 2020. **31**(7): p. 1384-1386.
603. Gérard, A., et al., *Remdesivir and acute renal failure: a potential safety signal from disproportionality analysis of the WHO safety database.* Clin Pharmacol Ther, 2020.
604. Strati, P., et al., *Ruxolitinib therapy is associated with improved renal function in patients with primary myelofibrosis.* Ann Hematol, 2019. **98**(7): p. 1611-1616.
605. Tefferi, A. and A. Pardanani, *Serious adverse events during ruxolitinib treatment discontinuation in patients with myelofibrosis.* Mayo Clin Proc, 2011. **86**(12): p. 1188-91.
606. Lee, E.B., *A review of sarilumab for the treatment of rheumatoid arthritis.* Immunotherapy, 2018. **10**(1): p. 57-65.
607. Yang, Y., et al., *Rapamycin prevents early steps of the development of diabetic nephropathy in rats.* Am J Nephrol, 2007. **27**(5): p. 495-502.
608. Inoki, K., et al., *mTORC1 activation in podocytes is a critical step in the development of diabetic nephropathy in mice.* J Clin Invest, 2011. **121**(6): p. 2181-96.
609. Ebrahim, N., et al., *Mesenchymal Stem Cell-Derived Exosomes Ameliorated Diabetic Nephropathy by Autophagy Induction through the mTOR Signaling Pathway.* Cells, 2018. **7**(12).
610. Morelon, E., et al., *Sirolimus: a new promising immunosuppressive drug. Towards a rationale for its use in renal transplantation.* Nephrol Dial Transplant, 2001. **16**(1): p. 18-20.
611. Huber, T.B., G. Walz, and E.W. Kuehn, *mTOR and rapamycin in the kidney: signaling and therapeutic implications beyond immunosuppression.* Kidney Int, 2011. **79**(5): p. 502-11.
612. Fervenza, F.C., et al., *Acute rapamycin nephrotoxicity in native kidneys of patients with chronic glomerulopathies.* Nephrol Dial Transplant, 2004. **19**(5): p. 1288-92.

613. Saxena, V., et al., *Safety and efficacy of sofosbuvir-containing regimens in hepatitis C-infected patients with impaired renal function.* Liver Int, 2016. **36**(6): p. 807-16.
614. Verdoodt, A., et al., *Do Statins Induce or Protect from Acute Kidney Injury and Chronic Kidney Disease: An Update Review in 2018.* J Transl Int Med, 2018. **6**(1): p. 21-25.
615. Cohen, A.J., et al., *Impact of Statin Intake on Kidney Stone Formation.* Urology, 2019. **124**: p. 57-61.
616. Nelson, M., et al., *Fanconi syndrome and lactic acidosis associated with stavudine and lamivudine therapy.* AIDS, 2008. **22**(11): p. 1374-6.
617. Shaer, A.J. and A. Rastegar, *Lactic acidosis in the setting of antiretroviral therapy for the acquired immunodeficiency syndrome. A case report and review of the literature.* Am J Nephrol, 2000. **20**(4): p. 332-8.
618. Koch, R.O., et al., *Acute hepatic failure and lactate acidosis associated with antiretroviral treatment for HIV.* Wien Klin Wochenschr, 2003. **115**(3-4): p. 135-40.
619. Fernandez-Fernandez, B., et al., *Tenofovir nephrotoxicity: 2011 update.* AIDS Res Treat, 2011. **2011**: p. 354908.
620. Herlitz, L.C., et al., *Tenofovir nephrotoxicity: acute tubular necrosis with distinctive clinical, pathological, and mitochondrial abnormalities.* Kidney Int, 2010. **78**(11): p. 1171-7.
621. Chen, R., et al., *Association of Tenofovir Use With Risk of Incident Heart Failure in HIV-Infected Patients.* J Am Heart Assoc, 2017. **6**(4).
622. Mocroft, A., et al., *Estimated glomerular filtration rate, chronic kidney disease and antiretroviral drug use in HIV-positive patients.* AIDS, 2010. **24**(11): p. 1667-78.
623. Bonino, B., et al., *Uric acid in CKD: has the jury come to the verdict?* J Nephrol, 2020. **33**(4): p. 715-724.
624. Tsai, C.W., et al., *Serum Uric Acid and Progression of Kidney Disease: A Longitudinal Analysis and Mini-Review.* PLoS One, 2017. **12**(1): p. e0170393.
625. O'Donnell, K., et al., *Proteinuria is a Predictor of Renal Functional Decline in Patients with Kidney Cancer.* J Urol, 2016. **196**(3): p. 658-63.
626. Möller, C.C., et al., *Induction of TRPC6 channel in acquired forms of proteinuric kidney disease.* J Am Soc Nephrol, 2007. **18**(1): p. 29-36.
627. Yu, J., et al., *Tetrandrine Suppresses Transient Receptor Potential Cation Channel Protein 6 Overexpression- Induced Podocyte Damage via Blockage of RhoA/ROCK1 Signaling.* Drug Des Devel Ther, 2020. **14**: p. 361-370.
628. Chen, T., B. Ji, and Y. Chen, *Tetrandrine triggers apoptosis and cell cycle arrest in human renal cell carcinoma cells.* J Nat Med, 2014. **68**(1): p. 46-52.
629. Chen, S., et al., *Tetrandrine inhibits migration and invasion of human renal cell carcinoma by regulating Akt/NF-κB/MMP-9 signaling.* PLoS One, 2017. **12**(3): p. e0173725.
630. Tosi, P., et al., *Thalidomide alone or in combination with dexamethasone in patients with advanced, relapsed, or refractory multiple myeloma and renal failure.* European Journal of Haematology, 2004. **73**(2): p. 98-103.
631. Amirshahrokhi, K. and A.R. Khalili, *Thalidomide ameliorates cisplatin-induced nephrotoxicity by inhibiting renal inflammation in an experimental model.* Inflammation, 2015. **38**(2): p. 476-84.
632. Santos, M.L.C., et al., *Nephrotoxicity in cancer treatment: An overview.* World J Clin Oncol, 2020. **11**(4): p. 190-204.
633. Jones, S.A., et al., *Interleukin-6 in renal disease and therapy.* Nephrol Dial Transplant, 2015. **30**(4): p. 564-74.
634. Komaba, H., et al., *Interleukin-6 receptor inhibition with tocilizumab in various renal involvements associated with multicentric Castleman's disease: a report of three cases.* NDT Plus, 2008. **1**(6): p. 423-426.
635. Maeshima, A., et al., *Efficacy of tocilizumab, a humanized neutralizing antibody against interleukin-6 receptor, in progressive renal injury associated with Castleman's disease.* CEN Case Rep, 2012. **1**(1): p. 7-11.

636. Magro-Checa, C., et al., *Successful use of tocilizumab in a patient with nephrotic syndrome due to a rapidly progressing AA amyloidosis secondary to latent tuberculosis.* Amyloid, 2011. **18**(4): p. 235-9.
637. Gupta, S., et al., *Tocilizumab Use in a Chronic Hemodialysis Patient for the Management of COVID-19-Associated Pneumonia and Acute Respiratory Distress Syndrome.* Case Rep Nephrol, 2020. **2020**: p. 8829309.
638. Stephen, S., Y.A. Park, and A. Chrysostomou, *Clinical benefits of Tocilizumab in COVID-19-related cytokine release syndrome in a patient with end-stage kidney disease on haemodialysis in Australia.* Nephrology (Carlton), 2020. **25**(11): p. 845-849.
639. Pereira, M.R., et al., *Tocilizumab for severe COVID-19 in solid organ transplant recipients: a matched cohort study.* Am J Transplant, 2020. **20**(11): p. 3198-3205.
640. Mella, A., et al., *Case series of six kidney transplanted patients with COVID-19 pneumonia treated with tocilizumab.* Transpl Infect Dis, 2020: p. e13348.
641. Blaising, J., S.J. Polyak, and E.I. Pécheur, *Arbidol as a broad-spectrum antiviral: an update.* Antiviral Res, 2014. **107**: p. 84-94.
642. Cheng, Y., et al., *Kidney disease is associated with in-hospital death of patients with COVID-19.* Kidney Int, 2020. **97**(5): p. 829-838.
643. Kabat-Koperska, J., et al., *Three clinical cases of nonrespiratory acidosis in kidney transplant recipients receiving anti-CMV therapy.* Ann Transplant, 2012. **17**(2): p. 135-40.
644. Boillat Blanco, N., et al., *Impact of a preemptive strategy after 3 months of valganciclovir cytomegalovirus prophylaxis in kidney transplant recipients.* Transplantation, 2011. **91**(2): p. 251-5.
645. Gao, X., et al., *The HIV protease inhibitor darunavir prevents kidney injury via HIV-independent mechanisms.* Sci Rep, 2019. **9**(1): p. 15857.
646. Sundar, K., et al., *Zidovudine-induced fatal lactic acidosis and hepatic failure in patients with acquired immunodeficiency syndrome: report of two patients and review of the literature.* Crit Care Med, 1997. **25**(8): p. 1425-30.
647. Clay, P.G., R.C. Rathbun, and L.N. Slater, *Management protocol for abacavir-related hypersensitivity reaction.* Ann Pharmacother, 2000. **34**(2): p. 247-9.
648. Mounzer, K., et al., *HLA-B*57:01 screening and hypersensitivity reaction to abacavir between 1999 and 2016 in the OPERA.* AIDS Res Ther, 2019. **16**(1): p. 1.
649. Song, B., et al., *An Animal Model of Abacavir-Induced HLA-Mediated Liver Injury.* Toxicol Sci, 2018. **162**(2): p. 713-723.
650. Blas-García, A., et al., *The purine analogues abacavir and didanosine increase acetaminophen-induced hepatotoxicity by enhancing mitochondrial dysfunction.* J Antimicrob Chemother, 2016. **71**(4): p. 916-26.
651. Gehrke, N., et al., *Hepatocyte-specific deletion of IL1-RI attenuates liver injury by blocking IL-1 driven autoinflammation.* J Hepatol, 2018. **68**(5): p. 986-995.
652. Petrasek, J., et al., *IL-1 receptor antagonist ameliorates inflammasome-dependent alcoholic steatohepatitis in mice.* J Clin Invest, 2012. **122**(10): p. 3476-89.
653. Mahamid, M., R. Mader, and R. Safadi, *Hepatotoxicity of tocilizumab and anakinra in rheumatoid arthritis: management decisions.* Clin Pharmacol, 2011. **3**: p. 39-43.
654. Mak, K.Y., et al., *ACE2 Therapy Using Adeno-associated Viral Vector Inhibits Liver Fibrosis in Mice.* Mol Ther, 2015. **23**(9): p. 1434-43.
655. Singh, S. and S.V. Rana, *Ascorbic acid improves mitochondrial function in liver of arsenic-treated rat.* Toxicol Ind Health, 2010. **26**(5): p. 265-72.
656. Wei, J., et al., *Association between Dietary Vitamin C Intake and Non-Alcoholic Fatty Liver Disease: A Cross-Sectional Study among Middle-Aged and Older Adults.* PLoS One, 2016. **11**(1): p. e0147985.
657. Abdel-Hamid, N.I., M.F. El-Azab, and Y.M. Moustafa, *Macrolide antibiotics differentially influence human HepG2 cytotoxicity and modulate intrinsic/extrinsic apoptotic pathways in rat hepatocellular carcinoma model.* Naunyn Schmiedebergs Arch Pharmacol, 2017. **390**(4): p. 379-395.
658. Lockwood, A.M., S. Cole, and M. Rabinovich, *Azithromycin-induced liver injury.* Am J Health Syst Pharm, 2010. **67**(10): p. 810-4.

659. Sarges, P., J.M. Steinberg, and J.H. Lewis, *Drug-Induced Liver Injury: Highlights from a Review of the 2015 Literature.* Drug Saf, 2016. **39**(9): p. 801-21.
660. Koshimichi, H., et al., *Safety, Tolerability, and Pharmacokinetics of the Novel Anti-influenza Agent Baloxavir Marboxil in Healthy Adults: Phase I Study Findings.* Clin Drug Investig, 2018. **38**(12): p. 1189-1196.
661. Keystone, E.C., et al., *Safety and efficacy of baricitinib at 24 weeks in patients with rheumatoid arthritis who have had an inadequate response to methotrexate.* Ann Rheum Dis, 2015. **74**(2): p. 333-40.
662. Tollefson, A.E., et al., *Cidofovir and brincidofovir reduce the pathology caused by systemic infection with human type 5 adenovirus in immunosuppressed Syrian hamsters, while ribavirin is largely ineffective in this model.* Antiviral Res, 2014. **112**: p. 38-46.
663. Albarazanji, K., et al., *Intestinal serine protease inhibition increases FGF21 and improves metabolism in obese mice.* Am J Physiol Gastrointest Liver Physiol, 2019. **316**(5): p. G653-G667.
664. Okuno, M., et al., *Prevention of rat hepatic fibrosis by the protease inhibitor, camostat mesilate, via reduced generation of active TGF-beta.* Gastroenterology, 2001. **120**(7): p. 1784-800.
665. Kumagai, J., et al., *Autoimmune hepatitis following drug-induced liver injury in an elderly patient.* Clin J Gastroenterol, 2016. **9**(3): p. 156-9.
666. Dai, C., et al., *Chloroquine ameliorates carbon tetrachloride-induced acute liver injury in mice via the concomitant inhibition of inflammation and induction of apoptosis.* Cell Death Dis, 2018. **9**(12): p. 1164.
667. Fang, H., et al., *Dual role of chloroquine in liver ischemia reperfusion injury: reduction of liver damage in early phase, but aggravation in late phase.* Cell Death Dis, 2013. **4**: p. e694.
668. Kershenobich, D., et al., *Treatment of cirrhosis with colchicine. A double-blind randomized trial.* Gastroenterology, 1979. **77**(3): p. 532-6.
669. Arrieta, O., et al., *Colchicine delays the development of hepatocellular carcinoma in patients with hepatitis virus-related liver cirrhosis.* Cancer, 2006. **107**(8): p. 1852-8.
670. Salehzadeh, F., F. Pourfarzi, and S. Ataei, *The Impact of Colchicine on The COVID-19 Patients; A Clinical Trial Study.* Research Square, 2020.
671. Eleftheriou, G., et al., *Colchicine-induced toxicity in a heart transplant patient with chronic renal failure.* Clin Toxicol (Phila), 2008. **46**(9): p. 827-30.
672. Abbott, C.E., R. Xu, and S.H. Sigal, *Colchicine-Induced Hepatotoxicity.* ACG Case Rep J, 2017. **4**: p. e120.
673. Stine, J.G. and J.H. Lewis, *Current and future directions in the treatment and prevention of drug-induced liver injury: a systematic review.* Expert Rev Gastroenterol Hepatol, 2016. **10**(4): p. 517-36.
674. Shimizu, H., et al., *Corticosteroid dose increase is a risk factor for nonalcoholic fatty liver disease and contralateral osteonecrosis of the femoral head: a case report.* BMC Musculoskelet Disord, 2019. **20**(1): p. 88.
675. Zhang, X.Q., et al., *Efficacy of short-term dexamethasone therapy in acute-on-chronic pre-liver failure.* Hepatol Res, 2011. **41**(1): p. 46-53.
676. Donald, S., et al., *Complete protection by high-dose dexamethasone against the hepatotoxicity of the novel antitumor drug yondelis (ET-743) in the rat.* Cancer Res, 2003. **63**(18): p. 5902-8.
677. Hassan, H.M., et al., *Dexamethasone Pretreatment Alleviates Isoniazid/Lipopolysaccharide Hepatotoxicity: Inhibition of Inflammatory and Oxidative Stress.* Front Pharmacol, 2017. **8**: p. 133.
678. Gabbia, D., et al., *Dexamethasone counteracts hepatic inflammation and oxidative stress in cholestatic rats via CAR activation.* PLoS One, 2018. **13**(9): p. e0204336.
679. Lettéron, P., et al., *Glucocorticoids inhibit mitochondrial matrix acyl-CoA dehydrogenases and fatty acid beta-oxidation.* Am J Physiol, 1997. **272**(5 Pt 1): p. G1141-50.
680. Gupta, A.P., et al., *Pancreastatin inhibitor activates AMPK pathway via GRP78 and ameliorates dexamethasone induced fatty liver disease in C57BL/6 mice.* Biomed Pharmacother, 2019. **116**: p. 108959.
681. Badawy, A.A. and S. Bano, *Elevation of Kynurenine Metabolites in Rat Liver and Serum: A Potential Additional Mechanism of the Alcohol Aversive and Anti-cancer Effects of Disulfiram?* Alcohol Alcohol, 2016. **51**(1): p. 20-6.

682. Zhou, B., et al., *Disulfiram combined with copper induces immunosuppression via PD-L1 stabilization in hepatocellular carcinoma.* Am J Cancer Res, 2019. **9**(11): p. 2442-2455.
683. Berlin, R.G., *Disulfiram hepatotoxicity: a consideration of its mechanism and clinical spectrum.* Alcohol Alcohol, 1989. **24**(3): p. 241-6.
684. Björnsson, E., H. Nordlinder, and R. Olsson, *Clinical characteristics and prognostic markers in disulfiram-induced liver injury.* J Hepatol, 2006. **44**(4): p. 791-7.
685. Wald, O., et al., *Involvement of the CXCL12/CXCR4 pathway in the advanced liver disease that is associated with hepatitis C virus or hepatitis B virus.* Eur J Immunol, 2004. **34**(4): p. 1164-74.
686. Gish, R.G., et al., *Safety and antiviral activity of emtricitabine (FTC) for the treatment of chronic hepatitis B infection: a two-year study.* J Hepatol, 2005. **43**(1): p. 60-6.
687. Lim, S.G., et al., *A double-blind placebo-controlled study of emtricitabine in chronic hepatitis B.* Arch Intern Med, 2006. **166**(1): p. 49-56.
688. Patil, R., et al., *Acute Liver Toxicity due to Efavirenz/Emtricitabine/Tenofovir.* Case Reports Hepatol, 2015. **2015**: p. 280353.
689. Lv, B., et al., *Entecavir promotes CD34$^+$ stem cell proliferation in the peripheral blood and liver of chronic hepatitis B and liver cirrhosis patients.* Discov Med, 2014. **18**(100): p. 227-36.
690. Lange, C.M., et al., *Severe lactic acidosis during treatment of chronic hepatitis B with entecavir in patients with impaired liver function.* Hepatology, 2009. **50**(6): p. 2001-6.
691. Manns, M.P., et al., *Long-term safety and tolerability of entecavir in patients with chronic hepatitis B in the rollover study ETV-901.* Expert Opin Drug Saf, 2012. **11**(3): p. 361-8.
692. Choi, J., et al., *Risk of Hepatocellular Carcinoma in Patients Treated With Entecavir vs Tenofovir for Chronic Hepatitis B: A Korean Nationwide Cohort Study.* JAMA Oncol, 2019. **5**(1): p. 30-36.
693. Noda, Y., R.D. Hughes, and R. Williams, *Effect of prostacyclin (PGI2) and a prostaglandin analogue BW 245C on galactosamine-induced hepatic necrosis.* J Hepatol, 1986. **2**(1): p. 53-64.
694. Mohammad, M., et al., *Famotidine inhibits glycogen synthase kinase-3beta: an investigation by docking simulation and experimental validation.* J Enzyme Inhib Med Chem, 2013. **28**(4): p. 690-4.
695. Gupta, N., C. Patel, and M. Panda, *Hepatitis following famotidine: a case report.* Cases J, 2009. **2**(1): p. 89.
696. Sangawa, H., et al., *Mechanism of action of T-705 ribosyl triphosphate against influenza virus RNA polymerase.* Antimicrob Agents Chemother, 2013. **57**(11): p. 5202-8.
697. Baranovich, T., et al., *T-705 (favipiravir) induces lethal mutagenesis in influenza A H1N1 viruses in vitro.* J Virol, 2013. **87**(7): p. 3741-51.
698. Wallace, D.J., et al., *Cholesterol-lowering effect of hydroxychloroquine in patients with rheumatic disease: reversal of deleterious effects of steroids on lipids.* Am J Med, 1990. **89**(3): p. 322-6.
699. Qiao, X., et al., *Hydroxychloroquine Improves Obesity-Associated Insulin Resistance and Hepatic Steatosis by Regulating Lipid Metabolism.* Front Pharmacol, 2019. **10**: p. 855.
700. Falcão, M.B., et al., *Case Report: Hepatotoxicity Associated with the Use of Hydroxychloroquine in a Patient with COVID-19.* Am J Trop Med Hyg, 2020. **102**(6): p. 1214-1216.
701. Abdel Galil, S.M., *Hydroxychloroquine-induced toxic hepatitis in a patient with systemic lupus erythematosus: a case report.* Lupus, 2015. **24**(6): p. 638-40.
702. Ogawa, Y., et al., *Soluble CD14 levels reflect liver inflammation in patients with nonalcoholic steatohepatitis.* PLoS One, 2013. **8**(6): p. e65211.
703. Brown, J.R., et al., *Idelalisib, an inhibitor of phosphatidylinositol 3-kinase p110δ, for relapsed/refractory chronic lymphocytic leukemia.* Blood, 2014. **123**(22): p. 3390-7.
704. Herzer, K., et al., *IFN-alpha-induced apoptosis in hepatocellular carcinoma involves promyelocytic leukemia protein and TRAIL independently of p53.* Cancer Res, 2009. **69**(3): p. 855-62.
705. D'Amico, E., et al., *Primary biliary cirrhosis induced by interferon-alpha therapy for hepatitis C virus infection.* Dig Dis Sci, 1995. **40**(10): p. 2113-6.
706. Maeda, T., et al., *Exacerbation of primary biliary cirrhosis during interferon-alpha 2b therapy for chronic active hepatitis C.* Dig Dis Sci, 1995. **40**(6): p. 1226-30.

707. Leclerc, S., et al., *Sarcoidosis and interferon therapy: report of five cases and review of the literature.* Eur J Intern Med, 2003. **14**(4): p. 237-243.
708. Akay, B.N., et al., *Cutaneous, pulmonary and hepatic sarcoidosis associated with autoimmune complications during interferon-alpha treatment for hepatitis C virus infection.* J Eur Acad Dermatol Venereol, 2006. **20**(4): p. 442-5.
709. Berardi, S., et al., *High incidence of allograft dysfunction in liver transplanted patients treated with pegylated-interferon alpha-2b and ribavirin for hepatitis C recurrence: possible de novo autoimmune hepatitis?* Gut, 2007. **56**(2): p. 237-42.
710. Kogure, T., et al., *Fulminant hepatic failure in a case of autoimmune hepatitis in hepatitis C during peg-interferon-alpha 2b plus ribavirin treatment.* World J Gastroenterol, 2007. **13**(32): p. 4394-7.
711. Fiel, M.I., et al., *Development of hepatic granulomas in patients receiving pegylated interferon therapy for recurrent hepatitis C virus post liver transplantation.* Transpl Infect Dis, 2008. **10**(3): p. 184-9.
712. Veit, O., et al., *First case of ivermectin-induced severe hepatitis.* Trans R Soc Trop Med Hyg, 2006. **100**(8): p. 795-7.
713. Kumar, B.S., et al., *A Wonder Drug in the Arsenal against COVID-19: Medication Evidence from Ivermectin.* Journal of Advances in Medicine and Medical Research, 2020. **32**(10): p. 30-37.
714. Wald, O., et al., *The CCR5Delta32 allele is associated with reduced liver inflammation in hepatitis C virus infection.* Eur J Immunogenet, 2004. **31**(6): p. 249-52.
715. Bruno, R., et al., *Hepatotoxicity and antiretroviral therapy with protease inhibitors: A review.* Dig Liver Dis, 2006. **38**(6): p. 363-73.
716. Sulkowski, M.S., *Drug-induced liver injury associated with antiretroviral therapy that includes HIV-1 protease inhibitors.* Clin Infect Dis, 2004. **38 Suppl 2**: p. S90-7.
717. Cao, R., et al., *Prevention of HIV protease inhibitor-induced dysregulation of hepatic lipid metabolism by raltegravir via endoplasmic reticulum stress signaling pathways.* J Pharmacol Exp Ther, 2010. **334**(2): p. 530-9.
718. Núñez, M., *Hepatotoxicity of antiretrovirals: incidence, mechanisms and management.* J Hepatol, 2006. **44**(1 Suppl): p. S132-9.
719. Shehu, A.I., et al., *Pregnane X receptor activation potentiates ritonavir hepatotoxicity.* J Clin Invest, 2019. **129**(7): p. 2898-2903.
720. Motoyama, S., et al., *Methylprednisolone inhibits low-flow hypoxia-induced mitochondrial dysfunction in isolated perfused rat liver.* Crit Care Med, 2003. **31**(5): p. 1468-74.
721. Marinó, M., et al., *Acute and severe liver damage associated with intravenous glucocorticoid pulse therapy in patients with Graves' ophthalmopathy.* Thyroid, 2004. **14**(5): p. 403-6.
722. Le Moli, R., et al., *Determinants of liver damage associated with intravenous methylprednisolone pulse therapy in Graves' ophthalmopathy.* Thyroid, 2007. **17**(4): p. 357-62.
723. Candelli, M., et al., *Steatohepatitis during methylprednisolone therapy for ulcerative colitis exacerbation.* J Intern Med, 2003. **253**(3): p. 391-2.
724. Weissel, M. and W. Hauff, *Fatal liver failure after high-dose glucocorticoid pulse therapy in a patient with severe thyroid eye disease.* Thyroid, 2000. **10**(6): p. 521.
725. Chen, X., et al., *The Molecular Aspect of Antitumor Effects of Protease Inhibitor Nafamostat Mesylate and Its Role in Potential Clinical Applications.* Front Oncol, 2019. **9**: p. 852.
726. Ryu, H.G., et al., *Nafamostat mesilate attenuates Postreperfusion Syndrome during liver transplantation.* Am J Transplant, 2011. **11**(5): p. 977-83.
727. Pereboom, I.T., et al., *Aprotinin and nafamostat mesilate in liver surgery: effect on blood loss.* Dig Surg, 2007. **24**(4): p. 282-7.
728. Jin, X., et al., *The values of coagulation function in COVID-19 patients.* PLoS One, 2020. **15**(10): p. e0241329.
729. Tao, H., et al., *Niclosamide ethanolamine-induced mild mitochondrial uncoupling improves diabetic symptoms in mice.* Nat Med, 2014. **20**(11): p. 1263-9.
730. Bhagat, H.A., et al., *N-substituted phenylbenzamides of the niclosamide chemotype attenuate obesity related changes in high fat diet fed mice.* PLoS One, 2018. **13**(10): p. e0204605.

731. Rossignol, J.F., et al., *Improved virologic response in chronic hepatitis C genotype 4 treated with nitazoxanide, peginterferon, and ribavirin.* Gastroenterology, 2009. **136**(3): p. 856-62.
732. Rossignol, J.F. and C. Bréchot, *A Pilot Clinical Trial of Nitazoxanide in the Treatment of Chronic Hepatitis B.* Hepatol Commun, 2019. **3**(6): p. 744-747.
733. Siriussawakul, A., A. Zaky, and J.D. Lang, *Role of nitric oxide in hepatic ischemia-reperfusion injury.* World J Gastroenterol, 2010. **16**(48): p. 6079-86.
734. Lang, J.D., et al., *Inhaled NO accelerates restoration of liver function in adults following orthotopic liver transplantation.* J Clin Invest, 2007. **117**(9): p. 2583-91.
735. Wang, K., et al., *iNOS/NO signaling regulates apoptosis induced by glycochenodeoxycholate in hepatocytes.* Cell Signal, 2011. **23**(10): p. 1677-85.
736. Iwakiri, Y. and M.Y. Kim, *Nitric oxide in liver diseases.* Trends Pharmacol Sci, 2015. **36**(8): p. 524-36.
737. Li, Y.N. and Y. Su, *Remdesivir attenuates high fat diet (HFD)-induced NAFLD by regulating hepatocyte dyslipidemia and inflammation via the suppression of STING.* Biochem Biophys Res Commun, 2020. **526**(2): p. 381-388.
738. Yi, S.G., et al., *Early Experience With COVID-19 and Solid Organ Transplantation at a US High-volume Transplant Center.* Transplantation, 2020. **104**(11): p. 2208-2214.
739. Zampino, R., et al., *Liver injury in remdesivir-treated COVID-19 patients.* Hepatol Int, 2020. **14**(5): p. 881-883.
740. Pinto, G., et al., *Liver Injury Resolution after Ruxolitinib Treatment in Newly Diagnosed Primary Myelofibrosis.* Ann Hematologists Oncol, 2017. **4**(8): p. 1163.
741. Huizinga, T.W., et al., *Sarilumab, a fully human monoclonal antibody against IL-6Rα in patients with rheumatoid arthritis and an inadequate response to methotrexate: efficacy and safety results from the randomised SARIL-RA-MOBILITY Part A trial.* Ann Rheum Dis, 2014. **73**(9): p. 1626-34.
742. Diseases, N.I.o.D.a.D.a.K. *LiverTox: Clinical and Research Information on Drug-Induced Liver Injury [Internet].* 2012; Available from: https://www.ncbi.nlm.nih.gov/books/NBK547964/.
743. Qian, Q., et al., *Sirolimus reduces polycystic liver volume in ADPKD patients.* J Am Soc Nephrol, 2008. **19**(3): p. 631-8.
744. Massoud, O. and R.H. Wiesner, *The use of sirolimus should be restricted in liver transplantation.* J Hepatol, 2012. **56**(1): p. 288-90.
745. Dyson, J.K., et al., *Liver toxicity associated with sofosbuvir, an NS5A inhibitor and ribavirin use.* J Hepatol, 2016. **64**(1): p. 234-8.
746. Jose, J., *Statins and its hepatic effects: Newer data, implications, and changing recommendations.* J Pharm Bioallied Sci, 2016. **8**(1): p. 23-8.
747. Thapar, M., M.W. Russo, and H.L. Bonkovsky, *Statins and liver injury.* Gastroenterol Hepatol (N Y), 2013. **9**(9): p. 605-6.
748. Björnsson, E. and R. Olsson, *Suspected drug-induced liver fatalities reported to the WHO database.* Dig Liver Dis, 2006. **38**(1): p. 33-8.
749. Gérard, Y., et al., *Hyperlactataemia during antiretroviral therapy: incidences, clinical data and treatment.* Therapie, 2003. **58**(2): p. 153-8.
750. Mokrzycki, M.H., et al., *Lactic acidosis associated with stavudine administration: a report of five cases.* Clin Infect Dis, 2000. **30**(1): p. 198-200.
751. Cornejo-Juárez, P., J. Sierra-Madero, and P. Volkow-Fernández, *Metabolic acidosis and hepatic steatosis in two HIV-infected patients on stavudine (d4T) treatment.* Arch Med Res, 2003. **34**(1): p. 64-9.
752. Miller, K.D., et al., *Lactic acidosis and hepatic steatosis associated with use of stavudine: report of four cases.* Ann Intern Med, 2000. **133**(3): p. 192-6.
753. Clark, S.J., et al., *Acute liver failure associated with antiretroviral treatment for HIV: a report of six cases.* J Hepatol, 2002. **36**(2): p. 295-301.
754. Igoudjil, A., et al., *High concentrations of stavudine impair fatty acid oxidation without depleting mitochondrial DNA in cultured rat hepatocytes.* Toxicol In Vitro, 2008. **22**(4): p. 887-98.

755. Fromenty, B. and D. Pessayre, *Inhibition of mitochondrial beta-oxidation as a mechanism of hepatotoxicity.* Pharmacol Ther, 1995. **67**(1): p. 101-54.
756. Feig, J.L., et al., *The antiviral drug tenofovir, an inhibitor of Pannexin-1-mediated ATP release, prevents liver and skin fibrosis by downregulating adenosine levels in the liver and skin.* PLoS One, 2017. **12**(11): p. e0188135.
757. Zhao, J., et al., *TAF and TDF attenuate liver fibrosis through NS5ATP9, TGFβ1/Smad3, and NF-κB/NLRP3 inflammasome signaling pathways.* Hepatol Int, 2020. **14**(1): p. 145-160.
758. Alhankawi, D., et al., *Tenofovir Alafenamide (TAF) Induced Liver Failure.* Gut and Gastroenterology, 2018.
759. Zhang, Z., et al., *The plant alkaloid tetrandrine inhibits metastasis via autophagy-dependent Wnt/β-catenin and metastatic tumor antigen 1 signaling in human liver cancer cells.* J Exp Clin Cancer Res, 2018. **37**(1): p. 7.
760. Luan, F., X. He, and N. Zeng, *Tetrandrine: a review of its anticancer potentials, clinical settings, pharmacokinetics and drug delivery systems.* J Pharm Pharmacol, 2020.
761. Gong, X., et al., *Tetrandrine attenuates lipopolysaccharide-induced fulminant hepatic failure in D-galactosamine-sensitized mice.* Int Immunopharmacol, 2010. **10**(3): p. 357-63.
762. Li, F., et al., *Molecular mechanisms involved in drug-induced liver injury caused by urate-lowering Chinese herbs: A network pharmacology study and biology experiments.* PLoS One, 2019. **14**(5): p. e0216948.
763. Shi, J.P., et al., *Acute and sub-chronic toxicity of tetrandrine in intravenously exposed female BALB/c mice.* Chin J Integr Med, 2016. **22**(12): p. 925-931.
764. Chien, C. and R. Chien, *Thalidomide induces complete remission of advanced hepatocellular carcinoma.* Advances in Digestive Medicine, 2014. **1**(2): p. 64-67.
765. Enomoto, N., et al., *Protective effect of thalidomide on endotoxin-induced liver injury.* Alcohol Clin Exp Res, 2003. **27**(8 Suppl): p. 2S-6S.
766. Dabak, V. and P. Kuriakose, *Thalidomide-induced severe hepatotoxicity.* Cancer Chemother Pharmacol, 2009. **63**(4): p. 583-5.
767. Fowler, R. and K. Imrie, *Thalidomide-associated hepatitis: a case report.* Am J Hematol, 2001. **66**(4): p. 300-2.
768. Hanje, A.J., et al., *Thalidomide-induced severe hepatotoxicity.* Pharmacotherapy, 2006. **26**(7): p. 1018-22.
769. Hamadani, M., D.M. Benson, and E.A. Copelan, *Thalidomide-induced fulminant hepatic failure.* Mayo Clin Proc, 2007. **82**(5): p. 638.
770. Vilas-Boas, F., et al., *Thalidomide-induced acute cholestatic hepatitis: case report and review of the literature.* Gastroenterol Hepatol, 2012. **35**(8): p. 560-6.
771. Nishimoto, N., et al., *Study of active controlled tocilizumab monotherapy for rheumatoid arthritis patients with an inadequate response to methotrexate (SATORI): significant reduction in disease activity and serum vascular endothelial growth factor by IL-6 receptor inhibition therapy.* Mod Rheumatol, 2009. **19**(1): p. 12-9.
772. Serviddio, G., et al., *Tocilizumab and liver injury in patients with COVID-19.* Therap Adv Gastroenterol, 2020. **13**: p. 1756284820959183.
773. Hammami, M.B., et al., *Clinical course of COVID-19 in a liver transplant recipient on hemodialysis and response to tocilizumab therapy: A case report.* Am J Transplant, 2020. **20**(8): p. 2254-2259.
774. Bannwarth, B. and C. Richez, *Clinical safety of tocilizumab in rheumatoid arthritis.* Expert Opin Drug Saf, 2011. **10**(1): p. 123-31.
775. Brazdilova, K., et al., *Prevalence and risk factors for drug-induced liver injury among patients with rheumatic diseases treated with biological therapy: a single-center experience.* Physiol Res, 2019. **68**(Suppl 2): p. S157-S163.
776. Drepper, M., L. Rubbia-Brandt, and L. Spahr, *Tocilizumab-Induced Acute Liver Injury in Adult Onset Still's Disease.* Case Reports Hepatol, 2013. **2013**: p. 964828.

777. Nakamura, H., et al., *Acute Hypertriglyceridaemia Caused by Tocilizumab in a Patient with Severe COVID-19.* Intern Med, 2020. **59**(22): p. 2945-2949.
778. Jiang, S., et al., *Liver Injury in Critically Ill and Non-critically Ill COVID-19 Patients: A Multicenter, Retrospective, Observational Study.* Front Med (Lausanne), 2020. **7**: p. 347.
779. Ying, B., et al., *Ganciclovir inhibits human adenovirus replication and pathogenicity in permissive immunosuppressed Syrian hamsters.* Antimicrob Agents Chemother, 2014. **58**(12): p. 7171-81.
780. Toth, K., et al., *Valganciclovir inhibits human adenovirus replication and pathology in permissive immunosuppressed female and male Syrian hamsters.* Viruses, 2015. **7**(3): p. 1409-28.
781. Abdallah, E.A., B. Al-Helal, and R. Asad, *Ganciclovir-induced acute liver injury in a patient with lupus nephritis.* Saudi J Kidney Dis Transpl, 2014. **25**(5): p. 1084-5.
782. Shah, I., *Adverse effects of antiretroviral therapy in HIV-1 infected children.* J Trop Pediatr, 2006. **52**(4): p. 244-8.
783. Acosta, B.S. and E.W. Grimsley, *Zidovudine-associated type B lactic acidosis and hepatic steatosis in an HIV-infected patient.* South Med J, 1999. **92**(4): p. 421-3.
784. Lynx, M.D., A.T. Bentley, and E.E. McKee, *3'-Azido-3'-deoxythymidine (AZT) inhibits thymidine phosphorylation in isolated rat liver mitochondria: a possible mechanism of AZT hepatotoxicity.* Biochem Pharmacol, 2006. **71**(9): p. 1342-8.
785. Saavedra-Lozano, J., et al., *Salvage therapy with abacavir and other reverse transcriptase inhibitors for human immunodeficiency-associated encephalopathy.* Pediatr Infect Dis J, 2006. **25**(12): p. 1142-52.
786. Abers, M.S., W.X. Shandera, and J.S. Kass, *Neurological and psychiatric adverse effects of antiretroviral drugs.* CNS Drugs, 2014. **28**(2): p. 131-45.
787. Rasmussen, L.D., et al., *Risk of cerebrovascular events in persons with and without HIV: a Danish nationwide population-based cohort study.* AIDS, 2011. **25**(13): p. 1637-46.
788. Bedimo, R.J., et al., *Abacavir use and risk of acute myocardial infarction and cerebrovascular events in the highly active antiretroviral therapy era.* Clin Infect Dis, 2011. **53**(1): p. 84-91.
789. Cavalli, G. and C.A. Dinarello, *Anakinra Therapy for Non-cancer Inflammatory Diseases.* Front Pharmacol, 2018. **9**: p. 1157.
790. Jyonouchi, H. and L. Geng, *Intractable Epilepsy (IE) and Responses to Anakinra, a Human Recombinant IL-1 Receptor Agonist (IL-1ra): Case Reports.* Journal of Clinical & Cellular Immunology, 2016. **7**(5).
791. Dilena, R., et al., *Therapeutic effect of Anakinra in the relapsing chronic phase of febrile infection-related epilepsy syndrome.* Epilepsia Open, 2019. **4**(2): p. 344-350.
792. Divani, A.A., et al., *Coronavirus Disease 2019 and Stroke: Clinical Manifestations and Pathophysiological Insights.* J Stroke Cerebrovasc Dis, 2020. **29**(8): p. 104941.
793. Haschke, M., et al., *Pharmacokinetics and pharmacodynamics of recombinant human angiotensin-converting enzyme 2 in healthy human subjects.* Clin Pharmacokinet, 2013. **52**(9): p. 783-92.
794. Iwata, N., et al., *Protective Effects of Oral Administrated Ascorbic Acid against Oxidative Stress and Neuronal Damage after Cerebral Ishcemia/Reperfusion in Diabetic Rats.* Journal of Health Science, 2010. **56**(1): p. 20-30.
795. Yildirim, M., M. Ayyildiz, and E. Agar, *Endothelial nitric oxide synthase activity involves in the protective effect of ascorbic acid against penicillin-induced epileptiform activity.* Seizure, 2010. **19**(2): p. 102-8.
796. Zhang, B., et al., *Azithromycin drives alternative macrophage activation and improves recovery and tissue sparing in contusion spinal cord injury.* J Neuroinflammation, 2015. **12**: p. 218.
797. Farooq, O., et al., *Azithromycin-induced agitation and choreoathetosis.* Pediatr Neurol, 2011. **44**(4): p. 311-3.
798. Hayden, F.G. and N. Shindo, *Influenza virus polymerase inhibitors in clinical development.* Curr Opin Infect Dis, 2019. **32**(2): p. 176-186.
799. Sanchez, G.A.M., et al., *JAK1/2 inhibition with baricitinib in the treatment of autoinflammatory interferonopathies.* J Clin Invest, 2018. **128**(7): p. 3041-3052.
800. Gavegnano, C., et al., *Baricitinib reverses HIV-associated neurocognitive disorders in a SCID mouse model and reservoir seeding in vitro.* J Neuroinflammation, 2019. **16**(1): p. 182.

801. Chittick, G., et al., *Short-term clinical safety profile of brincidofovir: A favorable benefit-risk proposition in the treatment of smallpox.* Antiviral Res, 2017. **143**: p. 269-277.
802. Pavlovic, D., et al., *Progressive multifocal leukoencephalopathy: current treatment options and future perspectives.* Ther Adv Neurol Disord, 2015. **8**(6): p. 255-73.
803. Patel, A., J. Patel, and J. Ikwuagwu, *Treatment of progressive multifocal leukoencephalopathy and idiopathic CD4+ lymphocytopenia.* J Antimicrob Chemother, 2010. **65**(12): p. 2489-92.
804. Ishikura, H., et al., *The proteinase inhibitor camostat mesilate suppresses pancreatic pain in rodents.* Life Sci, 2007. **80**(21): p. 1999-2004.
805. Cui, C.M., et al., *Chloroquine exerts neuroprotection following traumatic brain injury via suppression of inflammation and neuronal autophagic death.* Mol Med Rep, 2015. **12**(2): p. 2323-8.
806. Sharma, O.P., *Effectiveness of chloroquine and hydroxychloroquine in treating selected patients with sarcoidosis with neurological involvement.* Arch Neurol, 1998. **55**(9): p. 1248-54.
807. Sato, K., et al., *Neuropsychiatric adverse events of chloroquine: a real-world pharmacovigilance study using the FDA Adverse Event Reporting System (FAERS) database.* Biosci Trends, 2020. **14**(2): p. 139-143.
808. Katsanos, A.H., et al., *Colchicine for stroke prevention in patients with coronary artery disease: a systematic review and meta-analysis.* Eur J Neurol, 2020. **27**(6): p. 1035-1038.
809. Gahm, C., et al., *Neuronal degeneration and iNOS expression in experimental brain contusion following treatment with colchicine, dexamethasone, tirilazad mesylate and nimodipine.* Acta Neurochir (Wien), 2005. **147**(10): p. 1071-84; discussion 1084.
810. Kuncl, R.W., et al., *Colchicine myopathy and neuropathy.* N Engl J Med, 1987. **316**(25): p. 1562-8.
811. Soto, O. and E.T. Hedley-Whyte, *Case records of the Massachusetts General Hospital. Weekly clinicopathological exercises. Case 33-2003. A 37-year-old man with a history of alcohol and drug abuse and sudden onset of leg weakness.* N Engl J Med, 2003. **349**(17): p. 1656-63.
812. Palopoli, J.J. and J. Waxman, *Colchicine neuropathy or vitamin B12 deficiency neuropathy?* N Engl J Med, 1987. **317**(20): p. 1290-1.
813. Pugin, D., et al., *COVID-19-related encephalopathy responsive to high-dose glucocorticoids.* Neurology, 2020. **95**(12): p. 543-546.
814. Ciriaco, M., et al., *Corticosteroid-related central nervous system side effects.* J Pharmacol Pharmacother, 2013. **4**(Suppl 1): p. S94-8.
815. Sugimura, Y., et al., *Protective effect of dexamethasone on osmotic-induced demyelination in rats.* Exp Neurol, 2005. **192**(1): p. 178-83.
816. Rock, R.B., et al., *Mycobacterium tuberculosis-induced cytokine and chemokine expression by human microglia and astrocytes: effects of dexamethasone.* J Infect Dis, 2005. **192**(12): p. 2054-8.
817. Warrell, D.A., et al., *Dexamethasone proves deleterious in cerebral malaria. A double-blind trial in 100 comatose patients.* N Engl J Med, 1982. **306**(6): p. 313-9.
818. Yeh, T.F., et al., *Early dexamethasone therapy in preterm infants: a follow-up study.* Pediatrics, 1998. **101**(5): p. E7.
819. Filosto, M., et al., *Disulfiram neuropathy: two cases of distal axonopathy.* Clin Toxicol (Phila), 2008. **46**(4): p. 314-6.
820. Enghusen Poulsen, H., et al., *Disulfiram therapy--adverse drug reactions and interactions.* Acta Psychiatr Scand Suppl, 1992. **369**: p. 59-65; discussion 65-6.
821. Mocco, J., et al., *O-desulfated heparin improves outcome after rat cerebral ischemia/reperfusion injury.* Neurosurgery, 2007. **61**(6): p. 1297-303; discussion 1303-4.
822. Nagata, K., et al., *Early low-anticoagulant desulfated heparin after traumatic brain injury: Reduced brain edema and leukocyte mobilization is associated with improved watermaze learning ability weeks after injury.* J Trauma Acute Care Surg, 2018. **84**(5): p. 727-735.
823. Xu, P., et al., *Combined Medication of Antiretroviral Drugs Tenofovir Disoproxil Fumarate, Emtricitabine, and Raltegravir Reduces Neural Progenitor Cell Proliferation In Vivo and In Vitro.* J Neuroimmune Pharmacol, 2017. **12**(4): p. 682-692.

824. Wynn, H.E., R.C. Brundage, and C.V. Fletcher, *Clinical implications of CNS penetration of antiretroviral drugs.* CNS Drugs, 2002. **16**(9): p. 595-609.
825. Song, J.H., et al., *[A Case of Severe Peripheral Polyneuropathy Occurring after Entecavir Treatment in a Hepatitis B Patient].* Korean J Gastroenterol, 2016. **67**(4): p. 216-219.
826. Koskinen, L.O., et al., *Prostacyclin treatment normalises the MCA flow velocity in nimodipine-resistant cerebral vasospasm after aneurysmal subarachnoid haemorrhage: a pilot study.* Acta Neurochir (Wien), 2009. **151**(6): p. 595-9; discussion 599.
827. Takahashi, C., et al., *Prostacyclin promotes oligodendrocyte precursor recruitment and remyelination after spinal cord demyelination.* Cell Death Dis, 2013. **4**: p. e795.
828. Dannon, P.N., et al., *Famotidine: a supplemental drug for the treatment of schizophrenia.* European Psychiatry, 1997. **12**(5): p. 263-264.
829. Janowitz, T., et al., *Famotidine use and quantitative symptom tracking for COVID-19 in non-hospitalised patients: a case series.* Gut, 2020. **69**(9): p. 1592-1597.
830. von Einsiedel, R.W., et al., *H(2)-histamine antagonist (famotidine) induced adverse CNS reactions with long-standing secondary mania and epileptic seizures.* Pharmacopsychiatry, 2002. **35**(4): p. 152-4.
831. Yoshimoto, K., et al., *Famotidine-associated central nervous system reactions and plasma and cerebrospinal drug concentrations in neurosurgical patients with renal failure.* Clin Pharmacol Ther, 1994. **55**(6): p. 693-700.
832. Świąder, M.J. and S.J. Czuczwar, *Interaction of famotidine, an H2 histamine receptor antagonist, with conventional antiepileptic drugs in mice.* Pharmacol Rep, 2014. **66**(3): p. 485-91.
833. Yamada, K., et al., *Reevaluation of the efficacy of favipiravir against rabies virus using in vivo imaging analysis.* Antiviral Res, 2019. **172**: p. 104641.
834. Wang, C.-C., et al., *Care for Patients with Stroke During the COVID-19 Pandemic: Physical Therapy and Rehabilitation Suggestions for Preventing Secondary Stroke*. 2020, Journal of Stroke and Cerebrovascular Diseases.
835. Koch, M.W., et al., *Hydroxychloroquine reduces microglial activity and attenuates experimental autoimmune encephalomyelitis.* J Neurol Sci, 2015. **358**(1-2): p. 131-7.
836. Gevers, S., et al., *Safety considerations for chloroquine and hydroxychloroquine in the treatment of COVID-19.* Clin Microbiol Infect, 2020. **26**(9): p. 1276-1277.
837. Sundelin, S.P. and A. Terman, *Different effects of chloroquine and hydroxychloroquine on lysosomal function in cultured retinal pigment epithelial cells.* APMIS, 2002. **110**(6): p. 481-9.
838. Melles, R.B. and M.F. Marmor, *The risk of toxic retinopathy in patients on long-term hydroxychloroquine therapy.* JAMA Ophthalmol, 2014. **132**(12): p. 1453-60.
839. Low, P.C., et al., *PI3Kδ inhibition reduces TNF secretion and neuroinflammation in a mouse cerebral stroke model.* Nat Commun, 2014. **5**: p. 3450.
840. Barbieux, S., et al., *Acute polyradiculopathy secondary to idelalisib in Relapsed Classical Hodgkin's lymphoma.* Curr Res Transl Med, 2018. **66**(3): p. 87-89.
841. Pavol, M.A., et al., *Pattern of neurobehavioral deficits associated with interferon alfa therapy for leukemia.* Neurology, 1995. **45**(5): p. 947-50.
842. Haroon, E., et al., *IFN-alpha-induced cortical and subcortical glutamate changes assessed by magnetic resonance spectroscopy.* Neuropsychopharmacology, 2014. **39**(7): p. 1777-85.
843. Valentine, A.D., et al., *Mood and cognitive side effects of interferon-alpha therapy.* Semin Oncol, 1998. **25**(1 Suppl 1): p. 39-47.
844. Mackenzie, C.D., T.G. Geary, and J.A. Gerlach, *Possible pathogenic pathways in the adverse clinical events seen following ivermectin administration to onchocerciasis patients.* Filaria J, 2003. **2 Suppl 1**: p. S5.
845. Chandler, R., *Serious Neurological Adverse Events after Ivermectin - Do They Occur beyond the Indication of Onchocerciasis?* American Journal of Tropical Medicine and Hygiene, 2018. **98**(2): p. 382-388.
846. Volpi-Abadie, J., A.M. Kaye, and A.D. Kaye, *Serotonin syndrome.* Ochsner J, 2013. **13**(4): p. 533-40.

847. DeSilva, K.E., et al., *Serotonin syndrome in HIV-infected individuals receiving antiretroviral therapy and fluoxetine.* AIDS, 2001. **15**(10): p. 1281-5.
848. Mas Serrano, M., et al., *Serotonin syndrome in two COVID-19 patients treated with lopinavir/ritonavir.* J Neurol Sci, 2020. **415**: p. 116944.
849. Stern, A.L., et al., *Differential Effects of Antiretroviral Drugs on Neurons In Vitro: Roles for Oxidative Stress and Integrated Stress Response.* J Neuroimmune Pharmacol, 2018. **13**(1): p. 64-76.
850. Gelati, M., et al., *High-dose methylprednisolone reduces cytokine-induced adhesion molecules on human brain endothelium.* Can J Neurol Sci, 2000. **27**(3): p. 241-4.
851. Cayli, S.R., et al., *Effect of combined treatment with melatonin and methylprednisolone on neurological recovery after experimental spinal cord injury.* Eur Spine J, 2004. **13**(8): p. 724-32.
852. Ghali, G.Z. and M.G.Z. Ghali, *Nafamostat mesylate attenuates the pathophysiologic sequelae of neurovascular ischemia.* Neural Regen Res, 2020. **15**(12): p. 2217-2234.
853. Ai, N., et al., *Niclosamide is a Negative Allosteric Modulator of Group I Metabotropic Glutamate Receptors: Implications for Neuropathic Pain.* Pharm Res, 2016. **33**(12): p. 3044-3056.
854. Brunaugh, A., et al., *Broad-Spectrum, Patient-Adaptable Inhaled Niclosamide-Lysozyme Particles are Efficacious Against Coronaviruses in Lethal Murine Infection Models.* bioRxiv, 2020.
855. Basu, P., et al. *A pilot study using nitazoxanide for hepatic encephalopathy in chronic liver failure [abstract].* in *59th Annual Meeting of the American Association for the Study of Liver Diseases*. 2008. San Francisco, CA.
856. Dirikolu, L., J.H. Foreman, and T. Tobin, *Current therapeutic approaches to equine protozoal myeloencephalitis.* J Am Vet Med Assoc, 2013. **242**(4): p. 482-91.
857. Rossignol, J.F., et al., *Effect of nitazoxanide in persistent diarrhea and enteritis associated with Blastocystis hominis.* Clin Gastroenterol Hepatol, 2005. **3**(10): p. 987-91.
858. Chen, Y., et al., *Astrocytes protect neurons from nitric oxide toxicity by a glutathione-dependent mechanism.* J Neurochem, 2001. **77**(6): p. 1601-10.
859. Gulati, G. and B.D. Kelly, *Does remdesivir have any neuropsychiatric adverse effects?* Ir J Psychol Med, 2020: p. 1-2.
860. Liang, C., et al., *A promising antiviral candidate drug for the COVID-19 pandemic: A mini-review of remdesivir.* Eur J Med Chem, 2020. **201**: p. 112527.
861. Haile, W.B., et al., *The Janus kinase inhibitor ruxolitinib reduces HIV replication in human macrophages and ameliorates HIV encephalitis in a murine model.* Neurobiol Dis, 2016. **92**(Pt B): p. 137-43.
862. Genovese, M.C., et al., *Long-term safety and efficacy of sarilumab plus methotrexate on disease activity, physical function and radiographic progression: 5 years of sarilumab plus methotrexate treatment.* RMD Open, 2019. **5**(2): p. e000887.
863. Burmester, G.R., et al., *Safety and efficacy of switching from adalimumab to sarilumab in patients with rheumatoid arthritis in the ongoing MONARCH open-label extension.* RMD Open, 2019. **5**(2): p. e001017.
864. Zeng, L.H., et al., *Rapamycin prevents epilepsy in a mouse model of tuberous sclerosis complex.* Ann Neurol, 2008. **63**(4): p. 444-53.
865. Lam, C., et al., *Rapamycin (sirolimus) in tuberous sclerosis associated pediatric central nervous system tumors.* Pediatr Blood Cancer, 2010. **54**(3): p. 476-9.
866. Mesci, P., et al., *Sofosbuvir protects human brain organoids against SARS-CoV-2.* bioRxiv, 2020.
867. Lashen, S.A. and M.I. Sanhoury, *Syncope Following Sofosbuvir Therapy for Chronic Hepatitis C: A Report of Two Cases.* Journal of Liver Research, Disorders & Therapy, 2016. **2**(4): p. 102-103.
868. Bhatia, H.K., et al., *Sofosbuvir: A novel treatment option for chronic hepatitis C infection.* J Pharmacol Pharmacother, 2014. **5**(4): p. 278-84.
869. Ifergan, I., et al., *Statins reduce human blood-brain barrier permeability and restrict leukocyte migration: relevance to multiple sclerosis.* Ann Neurol, 2006. **60**(1): p. 45-55.
870. Foudraine, N.A., et al., *Cerebrospinal-fluid HIV-1 RNA and drug concentrations after treatment with lamivudine plus zidovudine or stavudine.* The Lancet, 1998. **351**(9115): p. 1547-1551.

871. Schweinsburg, B.C., et al., *Brain mitochondrial injury in human immunodeficiency virus-seropositive (HIV+) individuals taking nucleoside reverse transcriptase inhibitors.* J Neurovirol, 2005. **11**(4): p. 356-64.
872. Scarsella, A., et al., *Stavudine-associated peripheral neuropathy in zidovudine-naïve patients: effect of stavudine exposure and antiretroviral experience.* Adv Ther, 2002. **19**(1): p. 1-8.
873. Patel, K., et al., *Impact of HAART and CNS-penetrating antiretroviral regimens on HIV encephalopathy among perinatally infected children and adolescents.* AIDS, 2009. **23**(14): p. 1893-901.
874. Ferrer, K. and N. Rakhmanina, *Neuropsychiatric effects of tenofovir in comparison with other antiretroviral drugs.* Neurobehavioral HIV Medicine, 2013. **5**(1): p. 1-10.
875. Pillay, P., et al., *Clinical diagnosis of sensory neuropathy in HIV patients treated with tenofovir: A 6-month follow-up study.* J Peripher Nerv Syst, 2019. **24**(4): p. 304-313.
876. Abdul Basit, S., et al., *Tenofovir alafenamide for the treatment of chronic hepatitis B virus infection.* Expert Rev Clin Pharmacol, 2017. **10**(7): p. 707-716.
877. Wang, J., et al., *Tetrandrine alleviates cerebral ischemia/reperfusion injury by suppressing NLRP3 inflammasome activation via Sirt-1.* PeerJ, 2020. **8**.
878. Jin, Q., et al., *Tetrandrine cytotoxicity and its dual effect on oxidative stress-induced apoptosis through modulating cellular redox states in Neuro 2a mouse neuroblastoma cells.* Life Sci, 2002. **71**(17): p. 2053-66.
879. Wulff, C.H., et al., *Development of polyneuropathy during thalidomide therapy.* Br J Dermatol, 1985. **112**(4): p. 475-80.
880. Clemmensen, O.J., P.Z. Olsen, and K.E. Andersen, *Thalidomide neurotoxicity.* Arch Dermatol, 1984. **120**(3): p. 338-41.
881. Briani, C., et al., *Thalidomide neurotoxicity: prospective study in patients with lupus erythematosus.* Neurology, 2004. **62**(12): p. 2288-90.
882. Offidani, M., et al., *Common and rare side-effects of low-dose thalidomide in multiple myeloma: focus on the dose-minimizing peripheral neuropathy.* Eur J Haematol, 2004. **72**(6): p. 403-9.
883. Tosi, P., et al., *Neurological toxicity of long-term (>1 yr) thalidomide therapy in patients with multiple myeloma.* Eur J Haematol, 2005. **74**(3): p. 212-6.
884. Cantarín-Extremera, V., et al., *Tocilizumab in pediatric refractory status epilepticus and acute epilepsy: Experience in two patients.* J Neuroimmunol, 2020. **340**: p. 577142.
885. Mizwicki, M.T., et al., *Tocilizumab attenuates inflammation in ALS patients through inhibition of IL6 receptor signaling.* Am J Neurodegener Dis, 2012. **1**(3): p. 305-15.
886. Kotch, C., D. Barrett, and D.T. Teachey, *Tocilizumab for the treatment of chimeric antigen receptor T cell-induced cytokine release syndrome.* Expert Rev Clin Immunol, 2019. **15**(8): p. 813-822.
887. Corominas, H., et al., *Correlation of fatigue with other disease related and psychosocial factors in patients with rheumatoid arthritis treated with tocilizumab: ACT-AXIS study.* Medicine (Baltimore), 2019. **98**(26): p. e15947.
888. Dastan, F., et al., *Promising effects of tocilizumab in COVID-19: A non-controlled, prospective clinical trial.* Int Immunopharmacol, 2020. **88**: p. 106869.
889. Jewell, P., et al., *Tocilizumab-associated multifocal cerebral thrombotic microangiopathy.* Neurol Clin Pract, 2016. **6**(3): p. e24-e26.
890. Haviernik, J., et al., *Arbidol (Umifenovir): A Broad-Spectrum Antiviral Drug That Inhibits Medically Important Arthropod-Borne Flaviviruses.* Viruses, 2018. **10**(4).
891. Bookstaver, P.B., et al., *Management of Viral Central Nervous System Infections: A Primer for Clinicians.* J Cent Nerv Syst Dis, 2017. **9**: p. 1179573517703342.
892. Curran, M. and S. Noble, *Valganciclovir.* Drugs, 2001. **61**(8): p. 1145-50 ; discussion 1151-2.
893. Simpson, D.M., *Human immunodeficiency virus-associated dementia: review of pathogenesis, prophylaxis, and treatment studies of zidovudine therapy.* Clin Infect Dis, 1999. **29**(1): p. 19-34.
